# Osteoarthritis: Epidemiology, Diagnosis, and Treatment

**DOI:** 10.1002/mco2.70834

**Published:** 2026-07-01

**Authors:** Qin Ru, Dongliang Yuan, Shide Jiang, Xiaoxuan Zhao, Xu Liu, Yusheng Li, Shuguang Liu, Yuxiang Wu

**Affiliations:** ^1^ Institute of Intelligent Sport and Proactive Health, Department of Health and Physical Education Jianghan University Wuhan Hubei China; ^2^ Deparment of Orthopedics Xiangya Hospital Central South University Changsha Hunan China; ^3^ National Clinical Research Center for Geriatric Disorders Xiangya Hospital Central South University Changsha Hunan China; ^4^ Department of Orthopedics The Central Hospital of Yongzhou Yongzhou Hunan China; ^5^ Xiangya School of Medicine Central South University Changsha Hunan China; ^6^ Department of Joint Surgery Honghui Hospital Xi'an Jiaotong University Xi'an Shaanxi China

**Keywords:** biomarker, imaging marker, osteoarthritis, treatment

## Abstract

Osteoarthritis (OA) is a highly prevalent, heterogeneous whole‐joint disorder that represents a leading cause of chronic pain, physical disability, and socioeconomic burden globally. It has emerged as a major public health challenge affecting millions of individuals. The pathogenesis of OA involves joint degeneration, biomechanical stress, systemic inflammation, metabolic disorder, subchondral bone remodeling, and cartilage matrix imbalance. Conventional diagnostic approaches fail to identify early‐stage pathological changes, while current clinical interventions primarily offer symptomatic relief with limited disease‐modifying efficacy. However, effective early biomarkers, clear stratification mechanisms, and precision targeted therapies for OA remain largely insufficient. Therefore, it is particularly imperative to elucidate the advances in the pathophysiological mechanisms of OA and explore novel biomarkers as well as targeted therapeutic strategies. This review systematically updates the latest epidemiological features of OA, assesses imaging and fluid‐derived biomarkers for early and accurate diagnosis, and reorganizes evidence‐based pharmacological, nonpharmacological, interventional, surgical, and emerging therapies. By clarifying the key pathophysiological characteristics of OA and conducting an in‐depth analysis of current diagnostic and therapeutic strategies, this review establishes a theoretical foundation for OA precision medicine, effectively accelerates the clinical translation of novel biomarkers and targeted therapies, and ultimately alleviates the global socioeconomic and disease burden attributable to OA.

## Graphical abstract

Osteoarthritis (OA) is a multifactorial degenerative joint disease and a leading cause of disability worldwide. This graphical abstract summarizes the epidemiology, diagnostic strategies, and therapeutic approaches of OA. OA development is driven by aging, metabolic dysregulation, and mechanical stress, leading to progressive joint degeneration. Diagnosis integrates clinical assessment, advanced imaging techniques, and emerging biomarkers. Current therapeutic strategies include nonpharmacological interventions, pharmacological treatments, and surgical management, highlighting the need for a comprehensive and multidisciplinary framework for OA management.

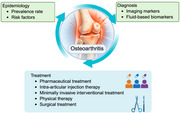

## 1 Introduction

Osteoarthritis (OA) stands as one of the most prevalent musculoskeletal disorders globally, imposing an escalating burden on individual well‐being, healthcare systems, and societal productivity [1]. As a chronic, progressive joint disease, OA differs fundamentally from autoimmune inflammatory arthropathies such as rheumatoid arthritis (RA) and is characterized by progressive articular cartilage deterioration accompanied by secondary pathological changes including subchondral bone sclerosis, osteophyte formation, synovial inflammation, and joint capsule stiffness [2, 3]. These pathological processes manifest as hallmark clinical symptoms such as chronic joint pain particularly with movement or weight‐bearing, reduced range of motion (ROM), morning stiffness, and in advanced stages, irreversible functional impairment that limits daily activities [4]. Beyond physical discomfort, OA contributes significantly to psychological distress, including anxiety and depression, further eroding quality of life (QoL) in affected individuals, particularly in those aged over 65 years [5]. With global prevalence rise alongside aging populations and obesity, OA has become a critical public health priority, necessitating continued research into its epidemiology, diagnosis, and therapeutics [6].

Over the past few decades, research into OA has evolved substantially and shifted from early studies focusing mainly on clinical manifestations and conventional radiological findings toward in‐depth investigations of pathogenic mechanisms, subtype stratification, early diagnosis, and targeted intervention [7]. OA is now recognized as a heterogeneous disorder with distinct subtypes based on etiology, anatomical location, and patient characteristics. Primary OA predominantly affects weight‐bearing joints such as the knees and hips, as well as small hand joints including the distal interphalangeal (DIP) joints, with prevalence increasing exponentially after the age of 50 years [8]. Secondary OA arises from triggers like joint trauma, uric acid metabolism disorders, chronic overuse, and obesity‐related biomechanical stress. Moreover, OA phenotypes vary significantly across joints due to differences in biomechanics, load distribution, and tissue architecture [9]. Notably, despite the recognition of OA heterogeneity, there remains an unmet clinical need for personalized intervention strategies based on patient subtypes, as current interventions are mostly generalized and fail to target the specific pathological characteristics of different OA subtypes. Current diagnosis relies primarily on traditional clinical assessment including detailed symptom history, physical examination, and conventional imaging [10]. With the development of molecular medicine and advanced imaging, the integration of advanced imaging modalities and soluble biomarkers has substantially improved detection accuracy and enabled earlier identification of disease [11, 12]. Nevertheless, prognostic models that effectively integrate clinical, imaging, and biomarker data remain underdeveloped, limiting the ability to identify high‐risk patients with rapid progression and to facilitate truly personalized interventions. More importantly, there is a lack of highly specific biomarkers for early OA diagnosis; existing biomarkers often lack sufficient specificity to distinguish early OA from other joint disorders or age‐related joint changes, leading to delayed diagnosis and missed opportunities for early intervention.

Currently, the management of OA adheres to stratified care principle, tailoring interventions to disease severity, age, comorbidities, and functional goals [13, 14]. Although available treatments can relieve pain and improve function, few can halt or reverse cartilage degeneration, and therapeutic responses vary widely among individuals. Pharmacological options such as nonsteroidal anti‐inflammatory drugs and intra‐articular corticosteroid (IACS) injections offer temporary pain relief but fail to modify disease trajectory. Surgical interventions, including joint replacement, remain the definitive treatment for end‐stage disease but are invasive, costly, and unsuitable for younger or multimorbid patients. Consequently, there exists an urgent and unmet need to advance targeted therapeutics, optimize drug bioavailability within the joint microenvironment, and develop approaches that promote cartilage regeneration, all these are essential to achieving personalized OA management and improving long‐term patient outcomes.

Despite significant advances in elucidating OA pathogenesis and refining clinical management, critical knowledge gaps continue to impede progress in the field. These include the pressing need for disease‐modifying drugs that can halt or reverse cartilage degeneration, the identification of more accurate and accessible biomarkers for early diagnosis and prognosis, and the development of robust predictive models to stratify patients according to their risk of rapid progression. Among these, the lack of highly specific early diagnostic biomarkers and the absence of personalized intervention strategies are the core unmet clinical needs in current OA management. Furthermore, the translation of preclinical discoveries into effective clinical therapies remains frustratingly slow, partly due to the complexity of OA pathophysiology and the lack of standardized outcome measures across trials. Given this context, this review was conducted to consolidate up‐to‐date evidence and address key clinical and scientific challenges in OA research, by systematically summarizing the latest findings in epidemiology, diagnosis, and treatment, and to clarify unresolved challenges that limit clinical progress.

This review first summarizes the latest epidemiological data across different OA subtypes, integrating global epidemic trends, regional disparities, and burden projections in aging societies. Then, cutting‐edge diagnostic markers are critically reviewed, with particular emphasis on novel imaging biomarkers, including advanced MRI sequences and quantitative radiographic techniques and body fluid markers detectable in serum, synovial fluid, and urine. Emerging therapeutic strategies are systematically examined, ranging from small‐molecule inhibitors and biologics targeting specific inflammatory and catabolic pathways to innovative drug delivery systems and regenerative medicine approaches such as mesenchymal stem cell (MSC) therapy and tissue engineering. Finally, it summarizes current limitations, technical bottlenecks, and future directions to provide a comprehensive reference for OA clinical management. By integrating the latest advancements in the epidemiology, diagnosis, and treatment of OA, this review aims to inform clinical practice and guide future research toward more effective and personalized OA management.

## 2 Epidemiology of OA

OA is one of the most prevalent chronic degenerative joint disorders worldwide, and its epidemiological profile exhibits a multidimensional and highly complex nature. The distribution and progression of OA are influenced by a broad spectrum of determinants, including etiological background, affected joint sites, age structure, sex differences, geographic and ethnic diversity, lifestyle patterns, and genetic as well as metabolic factors [15]. With the accelerated pace of global population aging, the continuous rise in obesity rates, and the widespread adoption of sedentary behaviors, the prevalence, incidence, and overall disease burden of OA have increased substantially in recent decades [16, 17]. According to findings from the Global Burden of Disease (GBD) studies, both the number of individuals living with OA and the disability‐adjusted life years attributable to the disease have shown a sustained upward trajectory over the past two decades, establishing OA as one of the leading contributors to long‐term functional disability and diminished QoL worldwide [18, 19].

Beyond its direct impact on joint structure and function, OA significantly compromises patients’ mobility, work capacity, and social participation, thereby imposing a substantial and persistent socioeconomic burden on healthcare systems. Consequently, a systematic understanding of the epidemiological characteristics of OA is essential not only for elucidating the underlying mechanisms of disease onset and progression but also for informing the development of disease prediction models, guiding public health planning, optimizing prevention strategies, and identifying novel therapeutic targets.

### 2.1 Epidemiological Characteristics by Etiological Classification

#### 2.1.1 Primary OA

**FIGURE 1:**
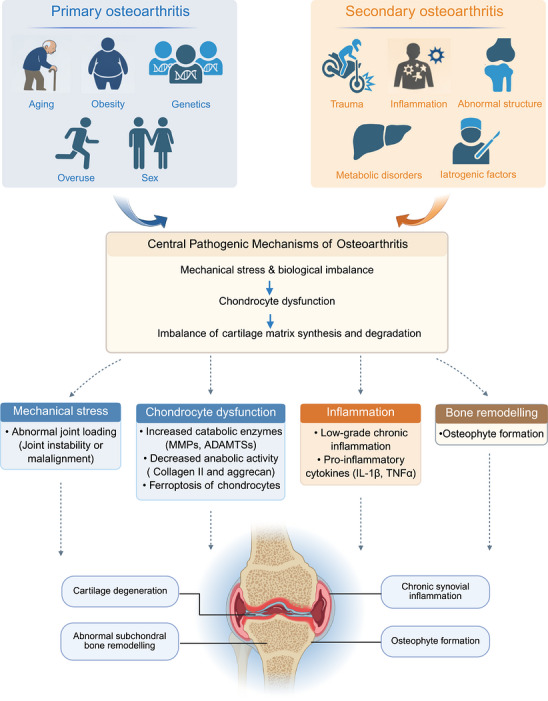
Risk factors and pathogenesis of osteoarthritis. Osteoarthritis is broadly classified into primary and secondary forms. Primary osteoarthritis is associated with aging, obesity, genetic predisposition, overuse, and sex‐related factors, whereas secondary osteoarthritis arises from identifiable causes, including trauma, inflammatory diseases, abnormal joint structure, metabolic disorders, and iatrogenic factors. These risk factors converge to induce mechanical stress and biological imbalance within the joint, leading to chondrocyte dysfunction and an imbalance between cartilage matrix synthesis and degradation. Key pathogenic mechanisms include abnormal joint loading, increased catabolic enzyme activity (MMPs and ADAMTSs), reduced anabolic activity of cartilage matrix components (collagen II and aggrecan), ferroptosis of chondrocytes, low‐grade chronic inflammation mediated by proinflammatory cytokines (e.g., IL‐1β and TNF‐α), and aberrant subchondral bone remodeling. Collectively, these processes result in characteristic pathological changes of osteoarthritis, including cartilage degeneration, chronic synovial inflammation, abnormal subchondral bone remodeling, and osteophyte formation. *Abbreviations*: MMPs: matrix metalloproteinases; ADAMTSs: a disintegrin and metalloproteinase with thrombospondin motifs, IL‐1β: interleukin‐1β; TNF‐α: tumor necrosis factor‐α.

Primary OA is the most prevalent subtype of the disease and is characterized by a complex and incompletely understood etiology. It is widely considered a multifactorial condition arising from the interplay of genetic predisposition [20], aging, sex differences [21, 22], obesity [23], chronic low‐grade inflammation [24], and long‐term alterations in joint biomechanical loading [25]. In women, the decline in estrogen levels after menopause impairs chondrocyte metabolic regulation and accelerates extracellular matrix (ECM) degradation, thereby markedly increasing the risk of OA [26]. Primary OA of the knee and small joints of the hand is particularly common in females, who also tend to exhibit more rapid disease progression than males (Figure 1).

Genetic factors have been consistently implicated in the development of primary OA. Kreitmaier et al. identified two methylation sites in low‐grade OA cartilage (cg17125990 and cg26736200) and one site in synovium (cg26736200) through colocalization and causal inference analyses, demonstrating their causal roles in tissue‐specific OA pathology [27]. Additionally, Steinberg and colleagues reported that 91 genes differentially expressed between low‐ and high‐grade cartilage are also associated with genetic susceptibility to OA [28].

Obesity is one of the most important and modifiable risk factors for primary OA. Its pathogenic effects are not limited to increased mechanical loading caused by excessive body weight, but are also closely associated with metabolic and inflammatory abnormalities mediated by adipose tissue. Adipose tissue secretes a variety of adipokines and proinflammatory cytokines, including interleukin‐6 (IL‐6), tumor necrosis factor‐ɑ (TNF‐ɑ), and leptin, which can disrupt cartilage homeostasis, promote synovial inflammation, and accelerate joint degeneration [29]. Recent studies have further demonstrated that higher visceral adiposity metabolic scores are significantly associated with an increased risk of OA, highlighting the important contribution of metabolic dysregulation to disease onset and progression [30]. Increasing evidence suggests that complex bidirectional interactions exist between OA and metabolic disorders. In addition to obesity, diabetes mellitus has also been recognized as an important metabolic comorbidity influencing OA progression. Chronic hyperglycemia may accelerate cartilage degeneration and joint inflammation through oxidative stress, mitochondrial dysfunction, and the accumulation of advanced glycation end‐products, all of which impair chondrocyte metabolism and ECM integrity. Moreover, the systemic low‐grade chronic inflammation associated with both obesity and diabetes may further amplify OA‐related pathological processes. Conversely, OA‐associated chronic pain and physical inactivity may lead to reduced mobility, thereby aggravating obesity, insulin resistance, and metabolic dysfunction, ultimately creating a vicious cycle between OA and metabolic diseases. These metabolic comorbidities not only influence OA onset and progression but also affect diagnostic evaluation and therapeutic outcomes. For instance, obesity may reduce the sensitivity of imaging assessments because of altered joint biomechanics and soft tissue interference, whereas diabetes is associated with delayed tissue healing, increased postoperative complications, and poorer functional recovery following joint replacement surgery. Therefore, multidisciplinary management strategies integrating metabolic control, weight management, physical rehabilitation, and inflammation modulation are essential for improving long‐term clinical outcomes in OA patients with metabolic comorbidities.

Collectively, the epidemiological profile of primary OA is characterized by high prevalence, marked female predominance, a steep age‐related increase in incidence, pronounced metabolic involvement, and clear familial and genetic susceptibility.

#### 2.1.2 Secondary OA

Secondary OA arises from identifiable structural abnormalities [31, 32], trauma [33], inflammatory arthritides [34], metabolic disorders, or crystal deposition diseases [35]. Compared with primary OA, secondary OA displays a clearer causal pathway and typically presents at a younger age [36]. Major subtypes include structural or congenital deformity‐related OA, metabolic and crystal deposition‐related OA, posttraumatic OA (PTOA), and inflammatory arthritis‐associated OA.

##### 2.1.2.1 Structural and Congenital Factors‐Related Secondary OA

A variety of structural joint abnormalities can alter local stress distribution and predispose individuals to OA, including developmental dysplasia of the hip (DDH), discoid meniscus of the knee [37], scoliosis [38], and congenital joint instability. In Asian populations, hip dysplasia is one of the most common causes of secondary hip OA in younger adults [39]. A multicenter cross‐sectional study in Japan reported that 73.8% of hip OA cases were attributable to DDH, making it the predominant etiology among newly admitted hospital cases [40]. Because structural deformities begin altering joint biomechanics early in life, symptom onset often occurs earlier than in primary OA, frequently between 30 and 40 years of age [41, 42].

These congenital structural abnormalities also exhibit sex‐specific patterns. For example, DDH is significantly more prevalent in women [39], whereas one study found that varus tibial alignment is more common in Asian men than in women [43]. OA resulting from structural deformities tends to progress rapidly, severely affecting QoL and often necessitating corrective osteotomy or joint replacement surgery [42].

##### 2.1.2.2 Metabolic and Crystal Deposition Diseases‐Related Secondary OA

Metabolic disorders constitute another major category of secondary OA, with gout‐related OA being the most representative example [44]. Gout is a metabolic disease characterized by deposition of monosodium urate crystals within joints [45]. Chronic hyperuricemia and recurrent gout flares lead to cartilage damage, bone erosion, and structural deformity, ultimately causing secondary OA. Gout‐related OA frequently involves multiple joints, including the first metatarsophalangeal (MTP1) joint, ankle, knee, elbow [46], and interphalangeal joints of the hand [47]. The MTP1 joint is particularly characteristic, often exhibiting coexisting cartilage destruction and osteophyte formation. Unlike primary OA, gout‐related OA commonly coexists with metabolic syndrome—including obesity, hypertension, hyperglycemia, and dyslipidemia—highlighting the strong influence of metabolic dysregulation on disease onset and severity [48].

The prevalence of gout has risen steadily over the past two decades [49] and displays clear geographic and sex disparities [50]. In Western countries, the overall prevalence is approximately 3–6% [44, 51], whereas in certain island populations such as Māori communities in New Zealand, it can reach 10–15% [52]. Although prevalence in Asian populations has historically been lower, recent increases in obesity, alcohol consumption, and high‐purine dietary patterns have contributed to a notable upward trend [53].

Gout‐related OA typically manifests earlier than primary OA. Men often develop symptoms between 40 and 50 years of age, whereas premenopausal women are relatively protected by estrogen‐mediated urate excretion and usually develop gout only after menopause. Female patients with gout are generally older and have a higher burden of comorbidities (hypertension, diabetes, kidney disease, obesity) than male patients. Moreover, risk factors differ by sex. Diuretic use is more common in women, whereas dietary triggers predominate in men [54]. With global aging and rising obesity rates, metabolic secondary OA is expected to account for an increasingly large proportion of the global OA burden.

##### 2.1.2.3 Posttraumatic OA

PTOA is one of the most common forms of secondary OA, with incidence ranging from 11 to 75% depending on the joint involved [55]. However, its incidence demonstrates substantial heterogeneity across different joints. Among these, the ankle joint exhibits the highest incidence, reaching approximately 70–75% following severe intra‐articular trauma. Knee PTOA is the second most common subtype, with reported incidence rates ranging from 11 to 50%, frequently occurring after anterior cruciate ligament (ACL) injury, meniscal injury (MI), or meniscectomy. In contrast, the incidence of PTOA following hip trauma is relatively lower. Posttraumatic ankle OA (AOA) is more common in men, which may be due to the fact that men are more prone to sports injuries, occupational traumas and military activities.

These interjoint differences are closely associated with variations in biomechanical characteristics, injury patterns, and tissue repair capacity. The ankle is a highly congruent, high‐load‐bearing joint in which the stress per unit articular surface area is substantially greater than that in the knee or hip. Consequently, fractures or ligament injuries [56] causing subtle joint incongruity may lead to marked focal stress concentration, thereby accelerating cartilage degeneration and joint deterioration [57, 58]. In addition, articular cartilage and the surrounding joint microenvironment exhibit distinct metabolic [59] and inflammatory [60] responses to trauma across different joints. The knee joint contains a more complex synovial environment and involves multiple intra‐articular structures, including the meniscus and cruciate ligaments; therefore, postinjury inflammatory responses and biomechanical imbalance tend to persist for prolonged periods. Even after ACL reconstruction partially restores joint stability, irreversible alterations in the intra‐articular biomechanical environment may remain. By comparison, ankle cartilage is relatively thin but may possess unique biochemical resistance to injury. Nevertheless, once mechanical stability is severely compromised, this protective characteristic is often insufficient to counteract the deleterious effects of abnormal joint loading. Furthermore, joint‐specific injury patterns also contribute to differences in PTOA risk. Knee PTOA commonly occurs after noncontact sports injuries in young individuals, particularly ACL rupture [61], whereas ankle PTOA is more frequently associated with high‐energy intra‐articular fractures, such as Pilon fractures, which often cause severe cartilage damage and articular surface incongruity at the time of initial injury. Therefore, the development of joint‐specific early intervention and long‐term follow‐up strategies is essential for reducing the risk of PTOA progression [62].

##### 2.1.2.4 Inflammatory Arthritis‐Associated Secondary OA

RA is a major cause of inflammatory secondary OA. Chronic synovitis in RA leads to cartilage erosion, bone destruction, and joint instability, and cumulative structural damage eventually results in degenerative changes resembling OA [63]. The prevalence of RA ranges from 0.5 to 1%, with incidence typically decreasing from northern to southern regions and from urban to rural areas. A positive family history increases RA risk by approximately threefold to fivefold, and increased concordance in twins further supports a substantial genetic contribution [64]. Because RA is more common in women, RA‐associated secondary OA also demonstrates a strong female predominance. Although the introduction of biologics and targeted DMARDs has significantly slowed structural deterioration in RA, these therapies cannot fully prevent secondary OA, particularly in patients with suboptimal inflammation control or metabolic comorbidities such as obesity and insulin resistance [65].

Overall, the epidemiological characteristics of secondary OA depend heavily on the underlying etiology. Compared with primary OA, secondary OA typically presents earlier, progresses more rapidly, imposes a greater functional burden, and exerts a more substantial socioeconomic impact, underscoring the need for heightened public health attention.

### 2.2 Epidemiological Characteristics by Anatomical Site

**FIGURE 2:**
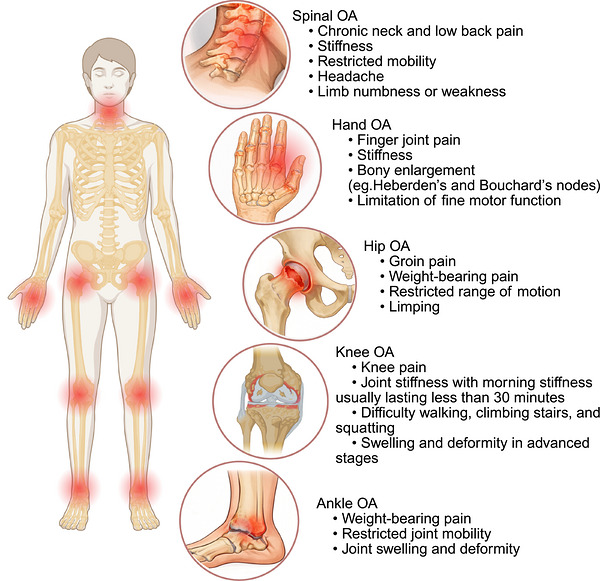
Major clinical manifestations of osteoarthritis at different anatomical sites. Osteoarthritis (OA) can affect multiple joints throughout the body and presents with site‐specific clinical features. Spinal osteoarthritis is characterized by chronic neck and low back pain, stiffness, restricted mobility, headache, and limb numbness or weakness. Hand osteoarthritis commonly manifests as finger joint pain, stiffness, bony enlargement (including Heberden's and Bouchard's nodes), and impairment of fine motor function. Hip osteoarthritis typically presents with groin pain, weight‐bearing pain, restricted range of motion, and limping. Knee osteoarthritis is characterized by activity‐ or weight‐bearing‐related knee pain, joint stiffness with morning stiffness usually lasting less than 30 min, difficulty walking, climbing stairs, and squatting, as well as swelling and deformity in advanced stages. Ankle osteoarthritis mainly presents with weight‐bearing pain, restricted joint mobility, and joint swelling or deformity. In hip osteoarthritis, weight‐bearing pain “primarily refers to deep groin or anterior thigh pain that is aggravated during prolonged standing or walking. In contrast, weight‐bearing pain” in ankle osteoarthritis mainly manifests as localized pain during gait initiation, stair climbing, or walking on uneven surfaces.

OA exhibits heterogeneity in its epidemiological profiles across different anatomical sites, with variations in global prevalence, demographic distribution, and associated disease burden being the most prominent features (Figure 2). These site‐specific differences not only reflect the unique biomechanical stresses and physiological characteristics of each joint but also have critical implications for targeted prevention, clinical management, and public health strategy formulation.

#### 2.2.1 Knee OA

Knee OA (KOA) is the most prevalent OA subtype, accounting for approximately four‐fifths of all OA cases worldwide [66]. According to the GBD study, the global prevalence of KOA in 2019 was approximately 4.9%, with a higher prevalence in women (6%) than in men. Among individuals aged ≥ 65 years, the prevalence increases sharply to 20–30%, making KOA a leading cause of disability in older adults. Significant geographic variation exists: the Western Pacific region exhibits the highest prevalence (7.64%; 9.83% in women and 5.45% in men), whereas the African region displays the lowest (1.82%; 1.99% in women and 1.63% in men). Higher‐income regions, including North America and Europe, generally report higher prevalence rates, partly due to increased obesity, population aging, changes in physical activity patterns, and improved diagnostic capabilities. Prevalence in Asian countries such as China and India is relatively lower but is rising rapidly due to accelerated aging and increasing obesity rates [67, 68]. Meta‐analyses have identified several risk factors for KOA, including prior knee injury, overweight or obesity, female sex, and age ≥ 40 years. Protective factors include regular moderate physical activity and higher educational attainment (high school or university level) [69, 70].

#### 2.2.2 Hip OA

Hip OA is among the most common and disabling degenerative joint diseases worldwide. Although its prevalence is lower than that of KOA, its functional impact and economic burden are often more profound [71, 72]. The etiology of Hip OA is multifactorial, involving genetic predisposition, advanced age, sex, obesity, abnormal mechanical loading, developmental hip abnormalities, and femoroacetabular impingement.

According to the 2020 GBD estimates, the global age‐standardized prevalence of Hip OA was approximately 417.7 cases per 100,000 population (95% uncertainty interval [UI] 314.7–532.7) [18], markedly lower than that of KOA but associated with a higher disability burden. Epidemiological studies consistently show that prevalence in Europe and North America is substantially higher than in Asian populations. China, Japan, and South Korea exhibit notably lower prevalence rates; however, rising trends have been documented due to lifestyle Westernization and increasing obesity rates [73]. Among African populations, hip OA appears to be the least prevalent globally, though data remain limited and may be influenced by disparities in healthcare access [74].

#### 2.2.3 Hand OA

Hand OA (HOA) is the most common peripheral form of arthritis worldwide. Based on GBD 2020 data, the age‐standardized prevalence of HOA was 2226.1 per 100,000 population (95% UI 1719.7–2802.8), second only to KOA [18]. HOA is a major cause of upper limb pain, functional impairment, and reduced QoL. Its prevalence increases markedly with age, rising sharply after 50 years and peaking in individuals aged ≥ 65 years. Clinically, HOA most commonly affects the DIP joints, proximal interphalangeal joints, and the first carpometacarpal joint, with DIP involvement frequently presenting as classic Heberden's nodes [75].

Globally, HOA displays a pronounced sex disparity, with prevalence rates two to four times higher in women than in men, particularly after menopause. This pattern is strongly associated with declining estrogen levels, altered hormonal metabolism, and reduced cartilage regenerative capacity. The marked increase in incidence between ages 55 and 75 years among women is well documented, with greater symptom severity and more pronounced inflammatory features. Genetic predisposition also plays a significant role; women with a family history of HOA demonstrate substantially elevated risk [76].

Occupational and biomechanical factors contribute significantly to HOA risk. Individuals engaged in repetitive gripping, pinching, or fine‐motor tasks—such as textile workers or pianists—exhibit increased incidence [77]. Repetitive microtrauma, mechanical overload, and joint instability further accelerate disease onset and progression. Although the hand is a nonweight‐bearing joint, obesity is an independent risk factor for HOA, mediated by systemic low‐grade inflammation triggered by adipokines such as IL‐6 and TNF‐α. Coexisting metabolic syndrome further amplifies this inflammatory milieu, promoting an inflammatory OA phenotype [78].

#### 2.2.4 OA at Other Anatomical Sites

Ankle OA (AOA) is relatively uncommon compared with knee and hip OA. Unlike KOA and hip OA—which are predominantly primary—approximately 70–80% of AOA cases are secondary to trauma, particularly fractures and severe ligament injuries [79]. Additional risk factors include obesity, malalignment of the ankle joint, chronic ankle instability, and structural abnormalities such as flatfoot or cavus foot deformities [57].

Temporomandibular joint (TMJ) OA (TMJ‐OA) is closely associated with TMJ disorders, trauma, and dental malocclusion. TMJ‐OA often presents with pain, joint sounds, and restricted mandibular motion and is more common in individuals with occlusal imbalance or parafunctional habits [80].

## 3 Diagnosis of OA

OA diagnosis is primarily based on clinical symptoms, supplemented by imaging examinations, and combined with laboratory tests to exclude the interference of similar diseases including RA, gout, infectious arthritis, and other diseases. Diagnostic criteria formulated by authoritative medical institutions, such as the American College of Rheumatology (ACR), European League Against Rheumatism (EULAR), and Osteoarthritis Research Society International (OARSI), vary slightly across countries or regions, with further differences tailored to the anatomical characteristics of affected joints (Table 1).

**TABLE 1:** Diagnostic criteria for osteoarthritis of different regions.

Guideline	Knee osteoarthritis	Hip osteoarthritis	Interphalangeal osteoarthritis	Shoulder osteoarthritis	Ankle osteoarthritis
Chinese guideline	1. Recurrent knee pain in past month 2. Age >50 years 3. Morning stiffness ≤30 min 4. Joint crepitus 5. Weight‐bearing X‑ray: joint space narrowing, subchondral sclerosis/cyst, osteophytes Diagnosis criteria: 1 + any 2 of 2–5 [81]	1. Recurrent hip pain in past month 2. Erythrocyte sedimentation rate (ESR) < 20 mm/h 3. X‐ray: osteophytes or acetabular hyperplasia 4. X‐ray: joint space narrowing Diagnosis criteria: 1+2+3 or 1+3+4 [81]	1. Recurrent interphalangeal pain/stiffness 2. Bony enlargement in >2 of 10 selected joints 3. Bony enlargement in >2 distal interphalangeal (DIP) joints 4. Swelling in <3 metacarpophalangeal (MCP) joints 5. Deformity in >1 of 10 selected joints 10 selected joints: bilateral index/middle finger DIP, proximal interphalangeal (PIP), and first carpometacarpal (CMC) joints Diagnosis criteria: 1 + any 3 of 2–5 [81]	1. Shoulder pain 2. Morning stiffness <30 min 3. Limited shoulder movement (especially external rotation) 4. Abduction/weight‐bearing X‐ray: joint space narrowing, osteophyte formation Diagnosis criteria: all criteria, exclude other diseases [81]	1. Weight‐bearing ankle pain 2. Limited dorsiflexion/plantar flexion 3. Joint swelling/deformity 4. Standing X‐ray shows subchondral bone sclerosis, osteophyte formation, joint space narrowing, or even deformity Diagnosis criteria: all criteria, exclude other diseases [81]
American College of Rheumatology (ACR) guideline	Clinical criteria, Knee pain + any 3 of 6: 1. Age >50 years 2. Stiffness <30 min 3. Joint crepitus 4. Bony tenderness 5. Bony enlargement 6. No palpable warmth Clinical plus radiographic, knee pain + any of 1 of 3: 1. Age >50 years 2. Stiffness <30 min 3. Osteophytes Clinical plus laboratory, knee pain + any 5 of 9: 1. Age >50 years 2. Stiffness <30 min 3. Joint crepitus 4. Bony tenderness 5. Bony enlargement 6. No palpable warmth 7. ESR <40 mm/h 8. Rheumatoid factors (RF) < 1:40 9. Synovial fluid (SF) consistent with osteoarthritis (clear, viscous, white blood cell count <2000/mm^3^) [82]	Clinical criteria, hip pain + any 1 of 2: 1. Hip internal rotation ≥ 15° with pain on rotation, morning stiffness ≤ 60 min, age > 50 years 2. Hip internal rotation < 15° + ESR ≤ 45 mm/h (or hip flexion ≤ 115° if ESR not available) Clinical plus radiographic: hip pain + any 2 of 3: 1. Osteophytes (femoral/acetabular) 2. Joint space narrowing (superior/axial/medial) 3. ESR < 20 mm/h [82]	Clinical plus radiographic, pain, aching, or stiffness + any 2 of 3: 1. Hard tissue enlargement in 10 selected joints 2. Swelling in <3 MCP joints 3. Hard tissue enlargement in ≥2 DIP joints OR < 2 enlarged DIP joints + deformity in ≥1 of 10 selected joints [83]	No specific ACR criteria	No specific ACR criteria
European League Against Rheumatism (EULAR) guideline	Clinical symptoms duration ≥3 months, with at least 1 of 3: 1. Persistent pain 2. Morning stiffness ≤30 min 3. Impaired knee function Clinical signs at least 1 of 3: 1. Crepitus 2. Limited joint movement (reduced active/passive range of motion, knee flexion < 110° or extension > 5°) 3. Bony enlargement (palpable hyperplasia of tibial plateau/femoral condyle, nonsoft tissue swelling) Radiographic (recommended, not mandatory) at least 1 of 3: 1. Joint space narrowing (medial > lateral) 2. Marginal osteophytes (tibial intercondylar spine, femoral condyle edge) 3. Subchondral bone sclerosis/cystic changes [84]	No specific EULAR criteria	Clinical symptoms: 1. Pain, aching, and/or stiffness in at least one target joint on most days of the previous 6 weeks 2. Joint symptoms are not be better explained by other injury or disease Overall hand osteoarthritis: ≥9 points (0–15 scale) Interphalangeal osteoarthritis and thumb base osteoarthritis: ≥8 points [85]	No specific EULAR criteria	No specific EULAR criteria

Imaging plays a pivotal role in OA diagnosis, encompassing radiography (X‐ray), computed tomography (CT), magnetic resonance imaging (MRI), and ultrasound. Among them, X‐ray remains the “gold standard,” characterized by narrowed joint space, subchondral bone hardening and/or cystic changes, and marginal osteophyte formation. CT with imaging features similar to X‐ray is predominantly used for differential diagnosis. MRI, by contrast, enables visualization of articular cartilage, intra‐ and peri‐articular soft tissues, facilitating early OA detection. Ultrasound, boasting high sensitivity and specificity for detecting synovitis and cartilage surface irregularities, is more cost effective and accessible than MRI, making it widely used in Europe and the United States for OA diagnosis and progression assessment. Laboratory tests, including blood routine, C‐reactive protein (CRP), rheumatoid factor, anticyclic citrullinated peptide antibody, joint fluid analysis, aid in excluding alternative arthropathies, and evaluating inflammatory severity, thereby supporting definitive OA diagnosis.

### 3.1 Imaging Markers

Imaging markers are core tools for quantifying joint structural degeneration, assessing inflammation activity, and predicting disease progression. Their classification and characteristics vary depending on the resolution and tissue specificity of the detection techniques, mainly including X‐ray, MRI, ultrasound, and CT‐derived markers. Among them, quantitative MRI (qMRI) techniques exhibit superior early diagnostic potential, and T2 mapping can detect abnormal cartilage hydration prior to morphological changes, while delayed gadolinium‐enhanced MRI of cartilage sensitively identifies proteoglycan loss 2–3 years before structural cartilage damage is observable. Ultrasound markers enable convenient visualization of cartilage surface irregularities and enhanced synovial blood flow, supporting early screening. Dual‐energy CT (DECT) further facilitates the detection of intra‐articular calcium crystals and early bony structural abnormalities, expanding the utility of imaging in OA assessment.

#### 3.1.1 X‐Ray Markers

X‐ray is a classic imaging modality for detecting bony structural changes in joints. Due to its high specificity, wide availability, and low cost, X‐ray is recognized as the gold standard for OA diagnosing recommended by EULAR and ACR. Core radiological indicators of OA include joint space narrowing (JSN), osteophyte formation, subchondral sclerosis, bone remodeling, and subluxation. The Kellgren–Lawrence (K–L) system is widely used to grade OA severity based on these X‐ray features and divided into five grades from 0 to IV. Grade 0 indicates completely normal radiographic performance of the joint without any osteoarthritic manifestations such as osteophytes, JSN, subchondral sclerosis, and joint malformation. Grade I refers to suspected OA, characterized only by tiny suspicious osteophytes and suspected JSN, without obvious bony sclerosis or joint deformity. Grade II represents mild OA with definite marginal osteophytes, minimal JSN, and no significant subchondral sclerosis or joint malformation. Grade III is moderate OA, manifested as moderate osteophytes, definite JSN, partial subchondral bone sclerosis, and possible bone cystic degeneration and mild joint malalignment. Grade IV is severe OA, presenting with massive huge osteophytes, significantly narrowed or even obliterated joint space, severe subchondral sclerosis and multiple cystic lesions, accompanied by obvious joint deformity, malalignment, or subluxation [86, 87]. Although the traditional K–L grading system can effectively reflect the overall severity of OA, it relies mainly on qualitative visual evaluation and lacks sufficient sensitivity to capture early subtle pathological changes. Therefore, exploring novel quantitative radiographic biomarkers is of great significance for the early detection, accurate evaluation and dynamic monitoring of OA.

##### 3.1.1.1 Joint Space Narrowing

JSN is a commonly used X‐ray markers, reflecting cartilage wear via reduced distance between weight‐bearing joint surfaces such as the knee–tibiofemeal joint and the hip–acetabular–femoral joint. It serves as a core basis for the K–L classification system. Santana et al. evaluated JSN progression in patients following meniscectomy and found that surgical patients exhibited faster JSN rates compared with those with meniscal tears managed nonoperatively, confirming JSN as a reliable indicator of PTOA progression [88]. Consistent with this, Lee et al. used JSN to evaluate OA progression during follow‐up, with results correlating closely with subsequent MRI findings [89], validating its utility for monitoring disease advancement.

##### 3.1.1.2 Osteophytes

Osteophytes are bony protrusions at joint margins or centers and pathognomonic bony markers of OA, formed by bone remodeling as compensation after cartilage degeneration. Their size can be used for semi‐quantitative grading of OA. Pan et al. developed an X‐ray‐based hierarchical classification model for automatic KOA severity grading, demonstrating that a geometric model integrating joint space parameters and osteophytes achieved 98.50% accuracy for K–L Grades 0–II and 81.65% for K–L Grades III–IV [90], highlighting osteophytes’ value in objective OA stratification.

##### 3.1.1.3 Subchondral Sclerosis

Subchondral sclerosis, characterized by increased subchondral bone density manifesting as reduced local bone radiolucency on X‐rays, is a direct consequence of abnormal bone remodeling and correlates positively with cartilage damage severity. It is most commonly observed in the medial tibial plateau (knee) and femoral head weight‐bearing area (hip). Han et al. performed X‐ray assessments in OA patients undergoing total knee arthroplasty (TKA) and identified abnormal remodeling and microstructural hardening of subchondral trabecular bone (STB). Moreover, STB bone volume fraction correlated with cartilage OARSI scores, indicating that subchondral sclerosis disrupts cartilage‐to‐STB load transmission, accelerating KOA progression [91]. Using advanced microfocus small‐angle X‐ray scattering with a spatial resolution of 5 µm, Finnila et al. further revealed increased mineral crystal thickness in subchondral bone of the lateral knee compartment in OA patients compared with healthy controls [92], providing mechanistic insights into sclerosis‐related OA pathogenesis. A key limitation of conventional X‐ray‐based sclerosis diagnosis is interobserver variability, which compromises result objectivity and reliability. To address this, Jin et al. developed an artificial intelligence (AI)‐enabled system for automatic grading of knee subchondral sclerosis using 4019 X‐ray images, achieving 84.70 ± 0.98% accuracy and 92.46 ± 0.49% specificity [93]. This confirms AI's potential to enhance the precision of sclerosis assessment. Additionally, single‐marker detection is prone to false positives due to suboptimal specificity/sensitivity, whereas multimarker combinations offer greater clinical utility. An AI‐based computer‐aided assessment system analyzing JSN, osteophytes, and sclerosis on 124 single‐knee X‐rays improved diagnostic consistency and overall accuracy among senior residents, while moderately enhancing diagnostic sensitivity for KOA [94].

#### 3.1.2 Ultrasonic Marker

Ultrasound enables visualization of tissue‐specific morphological changes prior to irreversible structural damage, making it pivotal for the early detection and assessment of OA. Due to its safety, cost effectiveness, and ability to simultaneously identify multiple joint abnormalities, ultrasound has emerged as a preferred imaging modality for many orthopedic clinicians in OA evaluation and monitoring. Ultrasound assess both soft tissue abnormalities such as joint effusion, synovial hypertrophy, Baker cysts, and bony structural changes, including reduced cartilage thickness, meniscus protrusion, and osteophyte formation. Beyond diagnostic utility in KOA, ultrasound also serve as imaging means with predictive value for long‐term disease progression [95]. EULAR developed guidelines for bone ultrasound in 2001 [96], and ACR released recommendations for the use of ultrasound in clinical practice in 2012 [97]. In OA diagnosis, gray‐scale and power Doppler ultrasound effectively detect synovial inflammation/hypertrophy, meniscus protrusion, osteophytes, and superficial articular cartilage alterations.

##### 3.1.2.1 Cartilaginous Changes

Ultrasound is used to quantify articular cartilage thickness and identifies surface/internal abnormalities. Early OA cartilage is characterized by indistinct contours and heterogeneous matrix echogenicity, while advanced disease presents with asymmetric narrowing of the cartilage band [98, 99]. Previous studies have confirmed strong correlations between ultrasound‐based cartilage grading and histological grades [100, 101]. A semi‐quantitative ultrasound scoring system for femoral condylar cartilage erosion severity correlates positively with clinical symptom and functional scores, including the Visual Analogue Scale (VAS), Lequesne Index, Western Ontario, and McMaster Universities Arthritis Index (WOMAC) scales, and pain subscales [102]. Thus, noninvasive knee joint ultrasound technology is a promising technique for screening and evaluating degenerative changes in articular cartilage.

##### 3.1.2.2 Bony Changes

Osteophyte formation is an early OA feature, detectable by ultrasound as hyperechoic signals at the bone margins attached to the joint capsule; in severe advanced disease, osteophyte bony contours are visualized. Ultrasound‐detected osteophytes show moderate‐to‐high consistency with X‐ray findings [103, 104]. A community‐based cross‐sectional study identified medial osteophytes among knee ultrasound‐detectable changes as closely associated with knee symptoms, while medial meniscal extrusion (MME) and medial osteophytes correlated significantly with knee scoring system (KSS) questionnaire symptom scores [105], confirming osteophytes as reliable predictors of symptomatic early KOA. Koski et al. developed a new ultrasound atlas for tibiofemoral osteophyte evaluation, comparing its consistency with conventional X‐ray. The atlas demonstrated repeatability comparable to X‐ray for detecting knee osteophytes, with higher sensitivity; notably, ultrasound‐detected osteophytes correlated significantly with arthroscopic medial knee cartilage changes, a relationship not observed with X‐ray‐detected osteophytes [106]. Another study also reported that ultrasound findings of femoral and acetabular osteophytes were consistent with X‐ray, while ultrasound outperformed X‐ray in detecting femoral head deformities [107]. In early KOA, ultrasound grades of medial/lateral femoral osteophytes correlate significantly with radiological grades, and ultrasound can detect osteophytes even in regions undetectable by X‐ray [108]. These findings highlight ultrasound's superior sensitivity for identifying cartilage damage and subtle osteophytes, particularly in early KOA. Riecke et al. developed a standardized musculoskeletal ultrasound procedure (MUS) to separately evaluate morphological changes, inflammatory manifestations, and joint effusion in medial/lateral knee compartments for KOA detection, and it was verified in a small cohort [109]. Results showed the MUS score had higher reliability and validity than standing knee X‐rays [109], though larger‐scale studies are needed to confirm its robustness. While X‐ray is the primary modality for evaluating joint space width (JSW), it has low sensitivity for longitudinal changes. A large multicenter study demonstrated ultrasound feasibility for knee JSW measurement, with JSW positively correlated with height and negatively correlated with age and body mass index (BMI) [110].

##### 3.1.2.3 Soft Tissue Markers

The synovium plays a more critical role in OA pathogenesis than previously recognized, with mild‐to‐moderate synovial effusion commonly detected in KOA patients [111]. Increased intra‐articular synovial fluid, known as joint effusion, has been reported to be associated with knee pain and OA severity [112]. A multicenter cross‐sectional study involving 600 symptomatic KOA patients found 41.7% patients had joint effusion [113], with synovial fluid volume positively correlated with ultrasound effusion grades and negatively correlated with ultrasound OA grades. Ultrasound outperforms clinical examination in effusion detection, supporting effusion volume as a potential OA diagnostic reference indicator [114]. Superior femoral fossa effusion and Baker's cyst are closely related to the pain manifestations of patients with primary KOA, serving as pain risk factors [115, 116]. Ultrasound findings indicate effusion and synovial hypertrophy are more prevalent in asymptomatic radiological OA (ROA) and symptomatic ROA (SROA) patients, with severity correlating with radiological grade; SROA patients exhibit more severe effusion and synovial hypertrophy than ROA patients [117]. A multicenter study revealed correlations between synovial hyperplasia, supine/upright MME, and Knee Injury and Osteoarthritis Outcome Score (KOOS) subscales; joint effusion was associated with KOOS scores, while suprapatellar recess hyperplasia and upright MME were independent predictors [118]. Ultrasound‐detected synovitis is also linked to KOA pain and predicts future medial cartilage loss, indicating its potential to forecast structural progression before overt cartilage degeneration [119]. Beyond diagnosis, MUS evaluates conservative treatment efficacy, demonstrating high diagnostic value in KOA [120]. Molyneux et al. developed a semi‐quantitative ultrasound imaging atlas to assess joint effusion, synovial hyperplasia, synovitis, osteophytes, JSW narrowing, and cartilage thickness in MTP1 joint OA. Interobserver consistency among six assessors exceeded 96%, highlighting the atlas's potential to detect early OA prognostic markers and inform clinical management [121].

#### 3.1.3 MRI‐Based Markers

MRI offers exceptional soft tissue resolution, enabling simultaneous assessment of lesions across multiple joint tissues such as cartilage, bone, synovium, and meniscus. Therefore, it is the preferred technique for the early diagnosis and precise research of OA. A growing array of MRI markers has demonstrated potential for early OA detection, progression prediction, and risk stratification for surgical intervention.

##### 3.1.3.1 MRI Markers for Early Diagnosis

Innovative MRI‐based techniques and analytical approaches have expanded the toolkit for early OA diagnosis, addressing the critical need for predictive biomarkers to assess disease onset. Beyond its widespread application in OA pathogenesis research, MRI‐derived methods, including dynamic/static contrast‐enhanced MRI, T2 mapping sequences, and three‐dimensional (3D) bone shape analysis, have shown robust diagnostic and prognostic potential [122, 123]. The narrative review by Daniela Herrera et al. highlighted semi‐quantitative metrics such as osteophyte scores as predictors of OA incidence. The MEAS prediction model, which integrates quantitative data such as volume, area, and distance of medial/lateral menisci, femoral/tibial cartilage and imaging markers including meniscal extrusion distance, infrapatellar fat pad (IPFP), and anatomical/spatial signal changes, also demonstrated high accuracy for OA incidence prediction [124, 125].

Convolutional neural networks (CNNs) are the latest technology for automatically evaluating KOA based on medical imaging data. Tack used CNNs to segment six knee anatomical structures (femur/tibia, femoral/tibial cartilage, bilateral menisci) and calculate quantitative features (cartilage volume, meniscal displacement, tibial coverage). The model achieved 99% balanced accuracy for differentiating nonarthritic and severe OA, and 84% for early KOA, validating its potential as an early diagnostic biomarker [126]. Using the 3D V‐Net deep learning architecture, T2 relaxation times of deep/superficial cartilage layers (lateral femur, lateral tibia, medial femur, medial tibia, and patella) effectively predicted KOA progression through the sagittal 2D multilayer multiecho spin echo sequence. T2 relaxation times showed strong predictive performance for rapid progression (within 12 months) but limited utility for slow progression (within 48 months). Notably, medial femoral cartilage T2 correlated most strongly with KOOS pain scores, and subjects in the top 25th percentile of tibial/femoral cartilage T2 values had a fivefold higher risk of radiographic OA within 2 years [127]. Yu et al. utilized the U‐Net architecture to automatically segment IPFP in weighted 2D turbo spin‐echo sequence, identifying radiomic features (first‐order intensity statistics, texture metrics, shape parameters) that predicted incident radiographic KOA (iRKOA) 1 year prior to clinical diagnosis [128]. These findings position IPFP radiomics as a promising early quantitative biomarker for iRKOA.

##### 3.1.3.2 MRI Markers for Predicting Progression

MRI features are increasingly recognized as reliable predictors of KOA progression, with semi‐qMRI scoring systems serving as cornerstones for multitissue joint assessment. Collins et al. analyzed 24‐month changes in semi‐qMRI biomarkers and their association with 48‐month radiological/pain progression in mild‐to‐moderate KOA [129]. They identified independent associations between progression and changes in cartilage area/thickness, Hoffa‐synovitis, effusion‐synovitis, and meniscal morphology, confirming these markers as both progression indicators and potential efficacy endpoints in clinical trials.

Nomograms based on 3D‐MRI bone shape changes facilitate prediction of radiological progression in mild‐to‐moderate OA within 24 months, doubling as monitoring tools for treatment efficacy [130, 131]. For AOA, calcaneofibular ligament (CFL) injuries disrupt talocrural joint compensation via abnormal stress distribution and osteophyte formation, leading to increased joint instability. MRI oblique views demonstrate a positive correlation between CFL injury and AOA severity, supporting CFL injury as a potential AOA progression marker [132]. Logistic regression analysis identified nomograms integrating MRI cartilage changes (thickness, volume, subchondral bone exposure area) as useful for predicting radiological and pain progression in mild‐to‐moderate OA. Among these, nomograms incorporating baseline and 24‐month MRI cartilage parameters (Nomogram 0 and Δ24) showed superior performance for radiological progression prediction compared with pain [133]. Artificial neural network analysis further confirmed that cartilage volume in the lateral femur, medial tibia, and lateral tibia predicts medial compartment joint space loss (JSL) [134]. Consistent with these findings, MacKay et al. reported that baseline and 12–18‐month MRI subchondral bone texture scores correlated significantly with 36‐month tibiofemoral radiographic JSL, with 12–18‐month scores showing better predictive performance for cartilage texture changes [135]. These results highlight subchondral bone morphology/texture analysis as a promising imaging biomarker for KOA. Additionally, MRI‐derived signal changes in femoral head cartilage can predict symptomatic KOA progression in asymptomatic individuals, serving as an early imaging biomarker [136].

Attur et al. performed routine X‐rays and 3T knee MRI in symptomatic KOA patients, finding that osteophyte scores predicted radiographic JSN. Combining osteophyte scores, MRI bone marrow lesions, and peripheral blood leukocyte inflammatory gene expression enhanced predictive value beyond any single biomarker [137], underscoring the utility of multimodal biomarkers for identifying high‐progression patients and guiding personalized care. Joseph et al. developed a risk calculator integrating baseline clinical data, X‐rays, and MRI to predict moderate/severe KOA progression in subjects with no/mild radiographic OA and minimal symptoms. Inclusion of X‐ray/MRI structural biomarkers and cartilage T2 relaxation time yielded acceptable performance for predicting radiographic (K‐L ≥ III) or symptomatic (WOMAC ≥ 5) progression [138]. Lin et al. similarly developed a nomogram combining MRI subchondral bone radiomic features and clinical characteristics to predict OA pain progression, successfully validating its utility for assessing vitamin D‐induced pain improvement. This radiomics‐based tool demonstrated strong predictive accuracy for differentiating pain response, offering a promising method to evaluate drug intervention efficacy [139]. Hunter et al. identified optimal combinations of imaging and biochemical biomarkers for KOA progression prediction, including MRI‐derived quantitative cartilage thickness/volume and semi‐quantitative bone shape/area markers, plus serum/urine biochemical markers such as matrix metalloproteinase‐3 (MMP‐3), Procollagen type IIA N‐terminal peptide (PIIANP), and C‐telopeptide of Type II collagen (CTX ‐ II). At 24 months, a panel comprising semi‐qMRI markers such as cartilage damage, meniscal morphology), qMRI markers including medial femoral central cartilage thickness, medial tibial cartilage volume, patellofemoral lateral bone area, and urinary N‐telopeptide of Type I collagen most effectively predicted progression [140]. These studies demonstrate that integrating multimodal biomarkers, encompassing imaging (MRI/X‐ray), clinical characteristics, biochemical indicators, and radiomic features, consistently enhances the accuracy of predicting OA progression, pain response, and treatment efficacy, highlighting the pivotal role of comprehensive biomarker panels in stratifying high‐risk patients and guiding precision OA management.

##### 3.1.3.3 MRI Markers for TKA Risk Prediction

With no clinically approved disease‐modifying OA drugs (DMOADs), most OA patients experience gradual progression over decades, with TKA as the definitive treatment for severe disease. MRI‐based biomarkers enable identification of TKA high‐risk population, particularly in early OA, facilitating timely interventions to slow progression and delay surgery. Wu et al. analyzed 839 early KOA patients (≥108 months follow‐up) from an OA program database, identifying 98 TKA cases. R‐based multivariate logistic regression revealed that glucocorticoid injection history, bone marrow lesions, meniscal displacement, cartilage defect area, extensor muscle strength, and contralateral KOA were independent predictors of rapid OA progression [141], providing a simplified framework for identifying TKA candidates. Kwoh et al. conducted a 7‐year follow‐up study on OA patients with K–L Grade Ⅱ or III and a medial minimum JSW of ≥2.5 mm. Among the 627 participants, 107 underwent TKA during the follow‐up period. TKA patients exhibited significantly greater total tibiofemoral/medial tibiofemoral cartilage thickness loss and medial JSW reduction compared with non‐TKA patients [142], confirming qMRI cartilage thickness and X‐ray JSW as predictors of knee disease progression. Medial femoral cartilage T2 relaxation time was significantly associated with 5‐year TKA incidence, serving as a TKA prediction marker; no such association was observed for lateral femoral, tibial, or patellar cartilage T2 values [127]. Tolpadi et al. developed a deep learning model integrating MRI, clinical, and demographic data, achieving 78.5 ± 0.134% accuracy, 81.8 ± 0.643% sensitivity, and 78.4 ± 0.138% specificity for TKA prediction. 3D qMRI outperformed 2D X‐rays in non‐OA and severe OA patients, demonstrating superior sensitivity/specificity for OA stratification, especially in non‐OA individuals [143]. Another study utilized a deep learning risk assessment model to predict the progression of KOA patients to TKA over a 108‐month follow‐up period. Compared with the traditional model that uses baseline clinical risk factors and the deep model that uses baseline knee X‐ray images, the deep learning model using baseline MRI has higher diagnostic performance in predicting TKA. Furthermore, the sensitivity and specificity of the TKA risk prediction model combining MRI and X‐ray were significantly higher than those of the models using X‐ray or MRI alone, and also superior to the traditional models based on baseline clinical risk factors [144].

#### 3.1.4 CT Makers

Due to the inability to depict soft tissue abnormalities, the application of CT in the assessment of OA is currently limited. However, advances in CT‐based quantitative analysis have expanded its utility: joint space mapping (JSM) on CT images enables 3D quantification of JSW, showing promise as an OA diagnostic indicator. A retrospective study measured 3D knee JSW via weight‐bearing CT (WB‐CT) and correlated results with X‐ray K–L grades, demonstrating the feasibility of WB‐CT for knee JSM and confirming associations between 3D JSW distribution and structural joint pathology [145]. Another study compared 3D tibiofemoral JSW analysis via WB‐CT, nonweight‐bearing CT (non‐WB‐CT), and WB‐radiography, finding WB‐CT combined with 3D analysis exhibited higher sensitivity for tibiofemoral JSW detection [146]. Additionally, recent research has validated CT for osteophyte volume quantification, with CT‐measured osteophyte volume directly correlating with OA severity [147]. DECT further enhances OA assessment. The DECT virtual noncalcification (VNCa) technique achieved >83.7% sensitivity and 99.5% specificity for detecting edematous bone marrow signal intensity (ELMSI) in KOA, with excellent interobserver consistency, supporting the utility of VNCa for qualitative/quantitative ELMSI analysis [148]. DECT also performs well in detecting bone marrow lesions (BMLs) in KOA, with VNCa‐derived BMLs severity positively correlated with patient pain levels [149]. Despite limitations in soft tissue visualization, WB‐CT and DECT have expanded the role of CT in OA evaluation, offering sensitive, quantitative insights into bony structural changes and pain‐related lesions.

#### 3.1.5 Dual‐Energy X‐Ray Absorptiometry Markers

Dual‐energy X‐ray absorptiometry (DXA)‐derived imaging markers have emerged as promising tools for OA risk stratification, leveraging large‐scale population data. Beynon et al. constructed a statistical shape model using 37,843 United Kingdom (UK) Biobank participants, identifying a DXA‐based knee shape marker associated with KOA progression. When combined with osteophyte data and demographics, this marker may facilitate identification of high‐risk individuals for TKA, enabling targeted interventions [150]. Another study based on UK Biobank statistical shape modeling to analyze correlations between DXA‐extracted knee shape patterns (KSMs) and radiographic OA grades; after adjusting for confounding factors, multiple KSMs were associated with OA grades, with stronger correlations observed in higher‐grade disease [151]. DXA‐derived knee shape markers, analyzed via statistical modeling, exhibit robust associations with KOA progression and OA severity, highlighting their potential for identifying high‐risk populations and guiding personalized OA management.

### 3.2 Fluid‐Based Biomarkers for OA

Current early OA diagnosis and staging rely on X‐ray examinations and clinical manifestations, with the K–L classification as the most widely used joint degeneration assessment tool [152]. While MRI can detect early joint abnormalities and injuries, its high cost and time‐consuming nature limit routine application [153, 154]. Notably, OA is typically diagnosed only after the onset of clinical symptoms, by which point irreversible structural damage has already occurred [155]. Further complicating diagnosis, overlapping clinical symptoms and pathological features make it difficult to distinguish OA from other arthritic disorders [156]. Therefore, early diagnosis is critical for effective OA management; however, the lack of highly sensitive and specific biomarkers for early, accurate detection highlights the urgent need to identify disease‐specific fluid‐based markers [157].

This review systematically summarizes the latest research progress on OA biomarkers, focusing on cytokines, adipokines, microRNAs (miRNAs), and metabolic biomarkers isolated from blood, urine, and synovial fluid, which offer noninvasive potential for early detection and differential diagnosis. Different fluid‐derived biomarkers have distinct application scenarios. Blood biomarkers are suitable for large‐scale screening, urine biomarkers are applicable for noninvasive long‐term monitoring, and synovial fluid biomarkers are used for the assessment of the local joint microenvironment.

#### 3.2.1 Cytokines

Cytokines are important molecules that regulate the immune response of the body and are closely related to the occurrence and development of OA, with ILs being the most extensively studied subgroup [158, 159]. Their diverse roles in mediating inflammation, cartilage catabolism, and bone remodeling make them promising candidates for OA biomarkers and therapeutic targets.

A clinical observational study identified significantly elevated plasma levels of IL‐2, IL‐4, and IL‐6 in KOA patients relative to healthy controls; notably, plasma IL‐4 and IL‐6 concentrations correlated positively with X‐ray‐assessed OA severity, indicating their potential as biomarkers for quantifying disease progression [160]. Complementarily, Silvestri et al. reported elevated serum soluble IL‐4 receptor levels in OA patients, with higher concentrations in large‐joint (knee/hip) OA compared with small‐joint (hand) OA; this suggests a compensatory mechanism to reduce IL‐4 bioavailability and mitigate its detrimental effects on chondrocyte functions [161]. However, this finding conflicts with Shan et al.’s observation of no correlation between IL‐4 and OA [162], highlighting unresolved inconsistencies that require validation in larger cohorts. IL‐6 is a cytokine with multiple functions and participates in the differentiation of osteoblasts and osteoclasts [163]. Detection of IL‐6 in synovial fluid of joints is helpful for the assessment of arthritis [164]. Doss et al. detected the IL‐6 content in the synovial fluid of patients with end‐stage OA who underwent TKA and found that the IL‐6 level in the synovial fluid of 16% of the patients was elevated [165]. A pilot randomized trial of patients with acute ACL injury found that PTOA and postreconstruction pain caused by ACL injury were positively correlated with the concentration of IL‐6 in synovial fluid [166]. These findings collectively position IL‐6 as a plasma/synovial fluid biomarker with potential for both severity grading and OA risk stratification.

Erosive HOA (eHOA) is a subtype of HOA, characterized clinically by hand pain and dysfunction, accompanied by inflammation and central joint erosion. Studies have shown that elevated IL‐7 levels are specifically associated with disease onset, suggesting IL‐7 mediates eHOA‐related inflammation and tissue damage [167]. IL‐7 emerges as a subtype‐specific biomarker for eHOA, offering potential for differential diagnosis of eHOA versus non‐eHOA.

IL‐17, a proinflammatory cytokine that upregulates other inflammatory mediators and adipokines, shows promise as both a serum and synovial fluid marker. Serum IL‐17 concentrations are higher in KOA patients [168]. The function of synovial fluid, as a key connection point between the systemic circulation and local tissues (including articular cartilage, subchondral bone, synovial membrane and infrapatellar lipid pad), is not merely to lubricate the joint. During OA, changes in the joint environment directly affect the expression of synovial genes, and the components of synovial fluid are influenced by disease drivers that cause symptoms (pain) and joint‐related pathologies. Therefore, synovial fluid is the most suitable biological fluid for studying the progression of OA [169]. Besides possibly serving as a potential serum marker for OA, Liu et al. found that the concentration of IL‐17 in the synovial fluid of OA patients was also significantly increased, and it was negatively correlated with the severity of OA [170]. Chen et al. confirmed that the concentrations of IL‐17 in the synovial fluid of patients with OA were significantly higher than those in the control group. Moreover, the concentration of IL‐17 in the synovial fluid within the joints of patients increased significantly with the K–L grade and was significantly positively correlated with the Lequesne index [168]. The median concentrations of IL‐6, leptin, resistin, C–C motif chemokine ligand 7 (CCL7), and nerve growth factor (NGF) levels in patients with higher IL‐17 levels in synovial fluid were significantly higher [171]. These findings indicate that IL‐17 in the serum and synovial fluid may play an important role in the pathogenesis of OA and has the potential to serve as a diagnostic marker for OA.

Shan et al. reported significantly higher serum IL‐21, IL‐17, and interferon‐γ levels in OA patients across disease grades compared with healthy individuals; notably, IL‐21^+^ follicular helper T cell expression correlated positively with OA grades, CRP levels, and WOMAC scores, indicating the potential of IL‐21 as an OA progression marker [162]. Another study reported elevated serum IL‐18 and IL‐20 in KOA patients, with IL‐18 concentrations positively correlating with cartilage catabolic markers such as MMP‐3 and cartilage glycoprotein 39 (YKL‐40) [172], further linking these ILs to OA‐related tissue degradation.

Beyond ILs, TNF‐α, a proinflammatory cytokine secreted by monocytes and macrophages, mediates inflammation, immune regulation, and cell proliferation via membrane receptor binding and nuclear signaling. Cross‐sectional studies demonstrate that higher TNF‐α concentrations are associated with increased OA risk, positioning it as a pathogenic biomarker [173]. Min et al. confirmed elevated serum TNF‐α in KOA patients, with receiver operating characteristic (ROC) curve analysis revealing 74.1% sensitivity and 76.0% specificity for OA diagnosis; univariate analysis further identified TNF‐α as an independent predictor of severe KOA [174]. TNF‐α exhibits robust potential as a diagnostic and prognostic biomarker, with high sensitivity and specificity to distinguish OA patients and predict severe disease.

Colony‐stimulating factor‐1 (CSF‐1) is a key mediator of macrophage activation, with synovial fluid CSF‐1 levels significantly higher in advanced KOA compared with early‐stage disease, correlating closely with inflammation and disease severity. Compared with healthy volunteers, serum CSF‐1, alongside IL‐6 and IL‐1β, is significantly elevated in KOA patients [175]. Mechanistically, CSF‐1 stimulates macrophage expression of colony‐stimulating factor‐1 receptor (CSF ‐ 1R), IL‐6, TNF‐α, IL‐1β, hypoxia‐inducible factor‐1α (HIF ‐ 1α), and MMP‐3, establishing it as a potential risk factor for OA progression to advanced stages [176]. Therefore, CSF‐1, particularly in synovial fluid, serves as a marker of advanced OA and a mediator of inflammatory cascades, offering value for identifying high‐risk patients.

Multicytokine panels outperform single‐marker detection in OA diagnosis due to enhanced specificity and sensitivity. Wang et al. found that serum IL‐6, TNF‐α, and leptin were significantly elevated in PTOA patients, with levels increasing proportionally with KL grade; their combination achieved an area under the curve (AUC) of 97% and sensitivity of 61%, comparable to X‐ray results [177]. Clinically, synovial fluid IL‐6, TNF‐α, and IL‐1β levels are significantly reduced following high tibial osteotomy (HTO), reflecting therapeutic response [166]. Additionally, synovial fluid IL‐40 is elevated in OA, correlates with synovial white blood cell count, and dose‐dependently stimulates chondrocyte secretion of proinflammatory factors IL‐6 and IL‐8 and MMP‐1, MMP‐3, and MMP‐13 [178]. The preclinical studies further demonstrated the potential of multicytokine combined detection as biomarkers. For instance, Castrogiovanni et al. observed elevated synovial fluid IL‐1, IL‐6, and TNF‐α in rats with ACL transection (ACLT)‐induced OA, with moderate exercise reducing these levels alongside disease improvement [179]. Ding et al. distinguished KOA from MI via synovial fluid cytokines, and OA patients exhibited higher IL‐6 and lower IL‐10 compared with MI patients [180], supporting cytokine‐based differential diagnosis. Multicytokine panels may enhance diagnostic accuracy and therapeutic response monitoring.

Cytokines, encompassing IL‐6, IL‐17, IL21, TNF‐α, and CSF‐1, demonstrate substantial potential as OA diagnostic, prognostic, and severity biomarkers, with multimarker panels offering superior performance. However, inconsistencies in correlative patterns and conflicting findings highlight the need for standardized, heterogeneous cohort studies to confirm their clinical utility.

#### 3.2.2 Adipokines

Adipose tissue functions not only as an energy reservoir but also as an active endocrine organ, secreting proinflammatory cytokines, hormones, and signaling molecules collectively termed adipokines (adipose‐derived) or adipocytokines (predominantly from adipocytes, with contributions from other cell types) [181]. Notably, adipokines are also synthesized by intra‐articular cells, including chondrocytes, synoviocytes, osteoblasts, stromal cells, macrophages, and immune cells [182], enabling both systemic and local regulation of OA pathogenesis. These molecules modulate key metabolic and immune processes, and in OA, they promote synovial inflammation, MMP production, cartilage degeneration, and bone remodeling. The expression of adipokine receptors on diverse joint cells further forms a complex adipose‐joint crosstalk network, making adipokines a focal point of recent OA research.

Leptin is a 16 kDa nonglycosylated protein, mainly synthesized and secreted by mature adipocytes in white adipose tissue. Studies have shown that serum/plasma leptin concentrations are higher in OA patients than healthy individuals [183], and synovial fluid leptin levels are similarly increased [184]. Furthermore, synovial fluid leptin upregulates lysyl oxidase like protein 3 expression in chondrocytes, induces chondrocyte apoptosis, and promotes the secretion of IL‐6 and IL‐8 [184]. The increase in leptin levels in synovial fluid also stimulates the secretion of MMP‐1 and MMP‐3, accelerating the degradation of ECM [185]. Leptin is elevated in both systemic and local joint fluids of OA patients, mediating chondrocyte damage and ECM degradation, thereby emerging as a potential biomarker and pathogenic mediator of OA.

Adiponectin is the most abundant adipokine synthesized in adipose tissue, which exerts anti‐inflammatory effects by shifting macrophages from a proinflammatory M1 phenotype to an anti‐inflammatory M2 phenotype [186, 187]. In contrast to leptin, OA patients exhibit reduced plasma adiponectin levels [188, 189]. A clinical study of female KOA patients confirmed decreased synovial fluid adiponectin, which correlated significantly with cartilage matrix degradation markers aggrecan, Aggrecan 1 (AGG1), AGG22 [190], indicating that adiponectin in synovial fluid may be involved in the regulation of cartilage matrix degradation in OA and may become a synovial marker of OA. Notably, a cross‐sectional study of 115 women with primary KOA found that synovial fluid adiponectin correlated more strongly with WOMAC pain/function scores and synovial IL‐6 levels than leptin after adjusting for confounders, highlighting its superior association with clinical severity and local inflammation [191]. Reduced adiponectin in plasma and synovial fluid of OA patients links to cartilage degradation and clinical severity, positioning it as a promising biomarker for OA‐related joint damage.

Resistin, also known as adipose‐specific secretory factor, contributes to OA pathogenesis by regulating production of proinflammatory cytokines such as TNF‐α, IL‐6, and IL‐12 in macrophages through NF‐kB and toll‐like receptor 4 (TLR4) pathway [192, 193, 194]. In KOA patients, synovial fluid resistin concentrations correlate positively with WOMAC pain/function/total scores, K–L grade, Noyes score, and Type II collagen C‐terminal peptide (a bone resorption marker), supporting its potential as a biomarker for OA severity and cartilage degeneration [195]. Calvet et al. further confirmed that synovial fluid resistin associates with joint dysfunction and stiffness, while adiponectin correlates with pain [196]. However, elevated synovial fluid resistin is also observed in RA [197, 198], limiting its specificity for OA. Therefore, lack of disease‐specificity necessitates large‐scale, standardized studies to validate utility of resistin as an OA biomarker.

Chemerin, also known as retinoic acid receptor response protein 2, is primarily expressed in white adipose tissue [199]. Clinical research has found that the data on the relationship between chemerin and OA is limited and there is some controversy. Vasileva linked serum chemerin to inflammatory marker changes in KOA [200], while Ma et al. reported elevated synovial fluid and synovial chemerin in OA patients, correlating with serum high‐sensitivity (HS) CRP (HS‐CRP), Outerbridge score, and Ayral score [201]. Huang et al. found that the levels of chemerin in patients with KOA were positively correlated with the severity of KOA [202]. Simsek Kaya et al. confirmed positive correlations between chemerin levels and OA severity, pain scores, and disease progression [203]. Contradicting these findings: Valcamonica found no difference in synovial fluid chemerin between immune‐mediated inflammatory arthritis and OA [204]. Eisinger et al. reported similar synovial fluid chemerin concentrations across OA, RA, and psoriatic arthritis (PsA)—with no induction of IL‐6 or MMP‐2/9 activity [205]. Chemerin shows potential as an OA severity biomarker, but inconsistent findings across arthritic conditions highlight the need for further research to clarify its specificity and pathogenic role.

The adipokine visfatin, also known as pre‐B‐cell colony‐enhancing factor 1, is expressed in synovial tissue, chondrocytes, osteophytes, osteoblasts, and osteoclasts [206, 207]. Patients with OA show higher levels of visfatin than healthy individuals [208]. Calvet et al. found that visfatin, similar to resistin, is closely related to the dysfunction of OA [196]. Fioravanti et al. observed higher serum visfatin in HOA, with significantly elevated levels in eHOA versus non‐eHOA [209]. Chen et al. confirmed elevated serum and synovial fluid visfatin in OA with synovial levels higher than serum, identifying the IPFP, synovium, and osteophytes as key local sources [210]. Critically, synovial fluid visfatin correlates positively with cartilage degradation biomarkers such as CTX‐II [210], suggesting involvement in degradation of cartilage matrix. Visfatin is elevated in systemic and local OA fluids, correlates with cartilage damage and erosive phenotypes, and holds promise as a biomarker for OA progression.

Vaspin is an abdominal fat‐derived serine protease inhibitor, and the level of vaspin in the blood or synovial fluid can reflect the expression level of the vaspin gene in joint tissues such as cartilage, synovial membrane, meniscus, subpatellar fat pad, and osteophytes in patients with OA. Most studies report reduced serum and synovial fluid vaspin in OA patients with synovial levels lower than serum [211]. Low vaspin levels mitigate IL‐1β‐induced catabolic molecule MMP‐2, MMP‐9, cyclooxygenase‐2, prostaglandin E_2_, and inflammatory factor expression, indicating anticatabolic and anti‐inflammatory properties [212]. In addition, vaspin may enhance the chondrogenic differentiation of bone marrow MSCs (BM‐MSCs) by activating the protein kinase B (PKB, AKT) pathway and stimulate the proliferation of chondrocytes and the production of ECM, thereby protecting chondrocytes under inflammatory conditions [213]. Vaspin can also regulate cartilage cholesterol metabolism through miR155/LXRalpha and participate in the occurrence and development of OA in rats [214]. However, conflicting evidence exists: Jeon et al. reported upregulated vaspin in OA cartilage, with vaspin knockout reducing OA development in mice and overexpression promoting cartilage destruction via p38/c‐Jun N‐terminal kinase (JNK) pathway activation and AP‐1‐driven catabolic factor production [215]. The role of vaspin in OA remains controversial, with evidence supporting both protective and pathogenic effects, necessitating further studies to resolve inconsistencies.

Fatty acid‐binding protein 4 (FABP4, adipocyte FABP/aP2) is an adipokine mainly expressed in adipocytes and macrophages, functioning as a lipid chaperone to facilitate intracellular lipid transport [216, 217]. Zhang et al. measured the concentration of FABP4 in patients with primary KOA at different stages and found significantly elevated plasma and synovial fluid FABP4 compared with controls [218]. Plasma FABP4 correlates closely with KOA severity [218], indicating systemic and local upregulation and potential as a promising OA biomarker.

Adipokines such as leptin, adiponectin, resistin, chemerin, visfatin, and FABP4 exhibit consistent associations with OA severity, cartilage damage, and clinical outcomes, supporting their utility as biomarkers. However, inconsistencies and limited disease‐specificity highlight the need for standardized, large‐scale studies to validate their clinical application.

#### 3.2.3 ECM‐Derived Markers

ECM is the structural foundation of articular cartilage, and its degradation, synthesis imbalance, or functional impairment directly drives the pathological progression of OA. ECM‐derived markers, which reflect dynamic changes in cartilage synthesis, breakdown, and protective functions, have emerged as critical tools for OA diagnosis, severity grading, and progression prediction. Below is a systematic summary of key ECM markers and their clinical potential in OA.

Fibrin‐3, also known as EFEMP1, is a secretory extracellular glycoprotein expressed in connective tissues, including blood and cartilage [219]. Fibrin‐3 regulates chondrocyte differentiation and ECM degradation by activating MMPs [220]. Functional studies confirm that fibulin‐3 inhibition upregulates chondrogenic markers SOX9, Type II collagen, and aggrecan in human articular chondrocytes, while its overexpression suppresses chondrogenesis in BM‐MSCs and ATDC5 chondrogenic cells [221]. Fibrin‐3 is specifically expressed in the superficial area of knee cartilage in humans and mice, and its expression level decreases with age [222, 223]. A 30‐month follow‐up study in elderly overweight women found that baseline serum levels of three fibulin‐3 epitopes (especially Fib3‐3) were significantly higher in OA incident cases than non‐OA controls, identifying fibulin‐3 as a predictive marker for OA onset [224]. Animal studies showed elevated serum Fib3‐3 after high‐fat diet‐induced cartilage injury, correlating with joint and cartilage degeneration [225]. A cross‐sectional study of 209 KOA patients and 165 controls confirmed increased serum and synovial fluid fibulin‐3, with multivariate logistic regression linking its levels to K–L grade [226]. Nassif found that the levels of fibrin‐3 in the serum and urine of female patients with primary KOA were significantly higher than those in the control group [227]. The levels of fibrin‐3 in serum and urine are positively correlated with the severity of pain in patients and negatively correlated with the Knee Outcome Survey Activities of Daily Living scale score and the K–L imaging grade [227]. These results indicate that fibrin‐3 can also serve as a biomarker for the severity of KOA, used to predict the progression of the disease. Compared with serum and synovial fluid, the detection of fibrin‐3 in urine is a more convenient method for tracking disease progression.

Lubricin, also known as supemcial zone protrin (SZP) or proteoglycan 4 (PRG4), is a viscous glycoprotein secreted primarily by chondrocytes and synoviocytes, localized to the synovium and synovial fluid [228, 229]. Lubricin protects chondrocytes by reducing joint friction, with expression downregulated after joint injury, consistent with its established role in OA pathogenesis [230, 231]. Clinical studies show reduced lubricin expression in the meniscus and synovial fluid of OA patients [232], while combined physical activity and olive oil‐rich diet not only improves OA but also upregulates the expression of lubricin in OA in synovial fluid [233]. Ogawa et al. found synovial fluid lubricin concentration correlated with knee joint anteroposterior mobility, total flexion angle, and ROM, but not with KL grade, suggesting a link to joint flexibility rather than clinical symptoms or disease severity [234]. The friction‐reducing function of lubricin depends on its glycosylation process. This glycosylation process can promote its interaction with lectin‐3 and maintain the stability of the cartilage lubrication layer [235]. Afshari et al. used glycoomics and glycoproteomics to characterize the glycoprotein forms of lubricants in synovial fluid and plasma of OA patients [236]. Sixty‐two types of mucoproteins were identified from the synovial fluid of OA patients, and 55 types were isolated from the plasma samples of OA patients [236]. Among them, lubricin was the most abundant mucoprotein in the synovial fluid of OA patients, accounting for 50% of the total strength of all mucoproteins, while in the plasma of OA, lubricin was the sixth most abundant mucoprotein, accounting for 6% of the total strength of all mucoproteins [236]. Notably, the longest lubricin splice variant is exclusive to synovial fluid, while shorter variants are plasma‐specific [236], indicating tissue‐specific glycosylation differences. Lubricin is downregulated in OA synovial fluid, with its levels linked to joint flexibility rather than severity; glycosylation differences between synovial fluid and plasma highlight its tissue‐specific roles in OA.

Collagen is the core structural component of cartilage, and imbalances in collagen synthesis/degradation underpin OA pathogenesis. The phenotypic changes of chondrocytes toward hypertrophy may be the basis for the onset of OA [237]. Among them, Type X collagen (ColX, also termed Col10) is upregulated during chondrocyte hypertrophy and is a potential marker of OA [238]. Clinical studies link serum ColX levels to KL grade, HS‐CRP, and cartilage degradation marker C2M in patients with OA [239]. In an ACLT‐induced OA dog model, serum ColX was significantly elevated at 8‐ and 12‐weeks postsurgery [240]. Additionally, urine ColX levels are higher in OA patients, with further elevation in those with higher K–L grades [241]. These data support ColX elevation in serum and urine correlates with OA severity and chondrocyte hypertrophy, supporting its potential as a diagnostic and progression marker.

Type II collagen is synthesized by chondrocytes and is the most abundant fibrous collagen in cartilage, forming the ECM framework that confers elasticity and compressive resistance [242]. Chondrocytes synthesize procollagen II, a precursor composed of three α1 (II) chains. After procollagen II is secreted outside the cell, its N‐terminal and C‐terminal propeptides are cleaved by specific enzymes. Ultimately, mature Type II collagen fibers (PIINP and PIICP) are formed and assembled into the cartilage matrix. Among them, PIINP is currently the most commonly used marker for Type II collagen synthesis and is released into the blood/body fluids after being cut [243]. Selective splicing of the first to third exons of PIINP generates two splicing variants, PIIANP and PIIBNP. Clinical studies have shown that baseline serum PIIANP correlates negatively with 2‐year radiological progression; serum PIIANP/PIIBNP levels are lower in high KL‐grade KOA patients [244]. Rousseau et al. also found that the reduced serum PIIANP increases radiological progression risk 3.4‐fold [245]. Patients with low levels of serum PIIANP and PIIBNP are 3.4 times more likely to develop radiological progression. This indicates that the low cartilage repair ability represented by the downregulation of serum PIIANP and PIIBNP is a risk factor for OA [244]. The mature Type II collagen is degraded by MMPs, forming two new peptide segments, whose lengths are 3/4 and 1/4 of the original mature Type II collagen, respectively. Clinical studies have significantly increased the content of C1 and 2C (also known as COL2‐3/4Cshort) in cartilage of patients with OA [246]. CTX‐II is also a characteristic fragment produced after the degradation of mature Type II collagen. When cartilage is damaged (such as in OA), MMPs and cysteine proteases specifically enzymatize the helical region of Type II collagen, and CTX‐II will be released into the blood, synovial fluid or urine. Therefore, an elevated level of CTX‐II indicates intensified destruction of cartilage matrix. CTX‐II can be used as a core marker reflecting the degree of cartilage destruction and is often employed for the monitoring of OA. Garnero et al. found that the level of CTX‐II in the urine of patients with hip OA was higher than that of the control group, and the average level of CTX‐II in the urine of patients with rapidly progressive OA was significantly higher than that of patients with slowly progressive OA. This indicates that CTX‐II may be helpful in identifying high‐risk patients with rapid progression of joint injury among those with hip OA [247]. Multiple meta‐analyses have shown that the level of CTX‐II in the urine of patients with KOA is significantly higher than that of the control group, and the level of CTX‐II in the urine of patients with severe KOA with K–L Grades III–IV is even higher [248, 249], indicating that the level of CTX‐II in urine may serve as a biomarker for KOA. Type II collagen‐derived markers (PIINP/PIIBNP, C1‐2C/CTX‐II) reflect cartilage metabolic imbalance, with CTX‐II and PIINP showing particular utility for assessing destruction and repair capacity, respectively.

Cartilage oligomeric matrix protein (COMP) is a noncollagen ECM protein that mainly exists in cartilage tissue [250, 251]. Elevated COMP levels were observed in both the serum and synovial fluid of patients with RA [252, 253]. Serum and synovial fluid COMP levels are also significantly elevated in patients with KOA, especially in the advanced stage of the disease. Moreover, the serum COMP level was significantly correlated with the severity of radiology as well as the measurement results of physical manifestations, knee pain and disability [254, 255]. Clinical retrospective comparative studies have also shown that the incidence of early cartilage lesions in patients with ACL deficiency is approximately 30%, and serum COMP increase in ACL‐deficient patients with early cartilage lesions [256], suggesting that serum COMP can be used to detect early cartilage changes. A meta‐analysis of nine studies also confirmed higher serum COMP in KOA, with levels increasing with disease severity [257]. These demonstrate the value of serum COMP as a diagnostic biomarker for KOA. Matejova et al. further linked synovial fluid COMP to OA pathological grade [258]. COMP is elevated in OA serum and synovial fluid, correlating with disease stage and early cartilage damage, making it a valuable marker for early diagnosis and prognosis.

A critical limitation of single ECM markers is their inability to distinguish primary OA from other inflammatory arthropathies causing cartilage degeneration. Ostojic et al. conducted a systematic review of blood and urine diagnostic biomarkers for early KOA and found that all biomarkers were studied only once in the selected papers. A set of biomarkers rather than a single one may be needed to distinguish patients with early‐stage knee arthritis from healthy controls [259]. A meta‐analysis confirmed serum COMP and urinary CTX‐II can distinguish knee/hip OA from controls, with serum COMP predicting progression [260]. Keter et al. used a dual biomarker (COMP and IL‐8) algorithm to distinguish primary OA from inflammatory arthritis. Based on 171 human knee joint synovial fluid samples, Keter et al. established the clinical decision limit of the ratio of COMP concentration to IL‐8 and verified it. They found that the clinical sensitivity and specificity of the dual biomarker algorithm in diagnosing OA were 87.0 and 88.9%, respectively [261]. Garnero et al. showed that combined baseline serum PIIANP (low) and CTX‐II (high) better predicts accelerated joint injury than either marker alone [262]. These results further support the concept of combined detection of OA biomarkers, indicating that the combination of multiple biomarkers may be more helpful for the diagnosis of OA. Further research requires strict research design and a larger sample size to verify the clinical application value of these markers. Beyond biochemical panels, Wang et al. developed a radiomics plus biochemical markers and clinical variables model (LBTRBC‐M) integrating 2922 MRI radiomics features, 17 biochemical biomarkers, and seven clinical variables to predict KOA progression [263]. LBTRBC‐M model achieved 70.1% accuracy for JSN and pain progression and improved resident physicians’ prognostic accuracy from 44.7–49.0 to 64.4–66.5% [263]. Combined biomarker panels and multimodal models overcome the limitations of single markers, significantly enhancing OA diagnostic specificity and prognostic accuracy.

ECM‐derived markers robustly reflect OA‐related cartilage synthesis, degradation, and functional changes. While single markers show promise for severity grading and progression monitoring, their clinical utility is optimized when combined with other biomarkers or multimodal data. Future research requires large‐scale, standardized studies to validate these markers and models for routine clinical application.

#### 3.2.4 miRNAs

miRNAs are noncoding RNA molecules that regulate gene expression levels by targeting the 3′UTR of mRNA to induce mRNA degradation or inhibit translation [264]. Their expression profiles dynamically change with the functional status of bone‐joint tissues and OA disease progression [265, 266], making them promising diagnostic, prognostic, and therapeutic response biomarkers for OA.

Serum miRNAs, as noninvasive biomarkers, have been extensively studied for OA risk stratification, diagnosis, and treatment monitoring due to their stability and easy accessibility. Ntoumou et al. performed miRNA array analysis on serum from OA patients and healthy controls, identifying 279 differentially expressed miRNAs; among these, hsa‐miR‐140‐3p, hsa‐miR‐33b‐3p, and hsa‐miR‐671‐3p were significantly downregulated, showing potential for evaluating OA risk and progression [267]. Liu et al. confirmed 17 downregulated miRNAs in 12 paired OA serum samples, with miR‐1202, miR‐33b‐3p, and miR‐940 proposed to regulate OA development via histone acetylation and cellular senescence pathways [268]. Notably, both studies reported reduced serum miR‐33b‐3p in OA, consistent with prior findings that miR‐33b inhibits chondrocyte monocyte chemoattractant protein‐1 (MCP‐1/CCL2) expression and regulates macrophage infiltration in OA [269]. In contrast, miR‐33‐5p was upregulated under aging conditions, and intra‐articular injection (IAI) of agomiR‐33‐5p induced cartilage loss and OA‐like changes [270], suggesting miR‐33 family members may exert stage‐ or age‐dependent roles in OA.

Kong et al. conducted miRNA microarray analysis on plasma from eight age‐ and gender‐matched KOA patients and controls, identifying 41 upregulated and 29 downregulated miRNAs; validation in 100 paired samples confirmed significant elevation of miR‐19b‐3p, miR‐122‐5p, miR‐320b, miR‐486‐5p, and miR‐92a‐3p in OA [271]. Subsequently, 100 patients with knee arthritis and 100 healthy controls matched by age and gender were recruited for further validation, and univariate and multivariate logistic analyses identified miR‐19b‐3p, miR‐122‐5p, and miR‐486‐5p as independent OA risk factors, with miR‐19b‐3p and miR‐486‐5p correlating positively with OA severity [271]. Case–control studies conducted by Yadav et al. further validated serum miR‐122‐5p as a KOA biomarker [272].

Subtype‐specific and differential diagnostic miRNAs have also been identified. Pertusa et al. reported upregulated miR‐497/miR‐423 and downregulated miR‐155/miR‐365 in hip OA serum, with miR‐497 showing superior diagnostic potential [273]. The differential diagnosis between eHOA and PsA is challenging, and specific diagnostic biomarkers are still lacking at present. Serum miR‐155 was significantly upregulated in both, with higher levels in eHOA than PsA, and combining miR‐155 with CRP enhanced differential diagnostic accuracy [274], further supporting the potential of miR‐155 as a diagnostic biomarker.

Functional studies link serum miRNAs to OA pathogenesis and severity. Compared with the normal group, serum miR‐130a levels was decreased in patients with OA, inducing chondrocyte apoptosis via upregulating Bax and caspase‐3/9, increasing inflammatory factors and inhibiting the phosphatase and tensin homolog (PTEN)/phosphatidylinositol 3‐kinase (PI3K) /AKT signaling pathway [275]. miR‐130a may serve as an important regulatory factor and potential biomarker for OA. Serum miR‐300 level is positively correlated with K–L grade, WOMAC pain score and functional score [276]. Therefore, the level of serum miR‐300 can reflect the severity of KOA and the degree of cartilage damage, and can be used as a potential biomarker to screen patients with OA. Rousseau et al. found serum miR‐146a‐5p was significantly increased in patients with KOA [277], while a 4‐year follow‐up of healthy individuals identified baseline serum miR‐186‐5p as a predictor of incident KOA [278], suggesting that miR‐146a‐5p and miR‐186‐5p were significantly associated with prevalent and incident OA, respectively. Wan et al. reported reduced plasma miR‐136 in patients with KOA, inversely proportional to the severity of the disease. As a direct target gene of miR‐136, the serum level of IL‐17 was significantly increased, which was consistent with the previous results. miR‐136 is involved in cartilage destruction by targeting IL‐17 [279].

Sclerostin (SOST) is a glycoprotein secreted by bone cells that affects the balance of bone metabolism by inhibiting the bone formation pathway. Serum miR‐153‐3p level in patients with KOA was significantly increased, and it was positively correlated with the levels of CRP and erythrocyte sedimentation rate (ESR), distinguishing OA from healthy controls via receiver operating characteristic curve analysis [280]. miR‐153‐3p promotes the progression of KOA by inhibiting SOST.

Beyond miRNA, circulating RNAs has also been proven to affect the pathological process of OA and show diagnostic potential. Serum Runx2‐derived hsa_circ_0005526 (circ_RUNX2) was significantly highly expressed in patients with OA, regulating the ECM of chondrocytes and mainly through sponging multiple miRNAs (miR‐361‐3p, miR‐498, miR‐665, and miR‐924), with ROC analysis supporting its diagnostic value [281].

Serum noncoding RNA can also be used to predict drug sensitivity. Dong et al. investigated the changes in the expression profiles of circulating miRNAs in the serum of patients with KOA before and after celecoxib treatment, screened out 45 upregulated and 48 downregulated miRNAs, and qPCR confirmed the increased levels of miR‐126‐5p and miR‐320a, and reduced miR‐155‐5p/miR‐146a‐5p [282]. Among them, the expression of miR‐126‐5p in patients with better clinical efficacy increased much more than that in nonclinical responders, and the level of miRNA‐320a decreased in nonclinical responders but increased in patients with better clinical efficacy, while the level of miRNA‐146a‐5p is inversely proportional to the clinical efficacy [282]. These results suggest that miR‐126‐5p, miR‐320a, and miR‐146a‐5p may be related to the therapeutic response of celecoxib and can be used as markers of drug treatment sensitivity. Metformin alleviates pain and improves OA while increasing the expression level of serum miR‐451 and reducing the levels of serum B‐cell lymphoma‐2 (BCL ‐ 2) and C‐X‐C motif chemokine ligand 16 (CXCL16) in patients. This suggests that metformin may alleviate pain and inflammation in patients with OA by regulating miR‐451/CXCL16 and BCL‐2. The level of serum miR‐451 may also serve as a marker for indirectly evaluating the efficacy of metformin in the treatment of OA [283].

Serum miRNAs such as miR‐33b‐3p, miR‐19b‐3p, miR‐155, and circ_RUNX2 exhibit robust potential for OA diagnosis, subtype differentiation, severity grading, and drug sensitivity assessment, with noninvasive detection facilitating clinical translation.

Synovial miRNAs, derived from synovial fluid, synovial tissue, or synovial extracellular vesicles, directly reflect local joint microenvironment changes, offering high specificity for OA assessment. Murata et al. analyzed miRNA expression in the synovial fluid and plasma of patients with OA and healthy controls and found that plasma miR‐16 and miR‐132 was significantly decreased. Synovial fluid miR‐16 can distinguish OA from RA [284]. Therefore, miR‐16 in synovial fluid and miR‐132 in plasma have the potential to serve as diagnostic biomarkers for RA and OA. Li et al. identified differential expression of miR‐23a‐3p, miR‐24‐3p, miR‐27a‐3p, miR‐27b‐3p, miR‐29c‐3p, miR‐34a‐5p, and miR‐186‐5p in synovial fluid between early and advanced KOA [285]. Tavallaee et al. observed that the expression of miR‐27b‐3p increased in the synovium of patients with KOA and OA mice. IAI of miR‐27b‐3p mimics induced synovial fibrosis‐like phenotype, with increased the synovitis score and the expression of COL1A1 and α‐smooth muscle actin (α‐SMA). Further studies have shown that miR‐27b‐3p may be involved in the pathogenesis of OA by regulating the ECM related to articular cartilage fibrosis through the PPARG/ADAMTS8 signaling [286]. miR‐29b‐3p was reported to be highly expressed in the synovial fluid of OA patients, and was positively correlated with age, BMI, and K–L line classification [287], suggesting that miR‐29b‐3p in synovial fluid may serve as a potential biomarker for OA.

Rizzi et al. detected significant differences in synovial fluid miR‐30b‐5p/miR‐140‐5p between OA and ACL tear patients, with miR‐30b‐5p differing specifically in males [288]. Compared with the control group, Yang et al. found that synovial fluid miR‐140 was significantly decreased in OA patients and negatively correlated with the severity of OA. Six months after arthroscopic clearance, the expression of miR‐140 in the synovial fluid of the patients returned to the normal level [289]. Yin et al. further confirmed that the expressions of miR‐140‐3p and miR‐140‐5p were both downregulated in the synovial fluid of OA patients and negatively correlated with disease severity [290], supporting their utility for diagnosis and clinical management.

HTO is a commonly used therapy for treating medial compartment KOA. Synovial miRNAs also predict treatment response. Ri et al. conducted a prospective study to investigate the differentially expressed miRNAs in the synovial fluid of patients with medial compartment KOA before and after HTO and found that miR‐29a‐3p, miR‐30a‐5p, and miR‐30c‐5p were differentially expressed. These results were verified in the synovial fluid derived from 22 patients [291]. Among them, miR‐30c‐5p is associated with postoperative pain relief and has the potential to serve as a prognostic biomarker [291].

Xie et al. evaluated the expression level of miR‐210 in joint synovial fluid samples from 10 healthy volunteers and 20 patients with early OA and 20 patients with advanced OA through real‐time PCR. They found that compared with healthy individuals, miR‐210 was significantly upregulated in both early OA and advanced OA patients [292], suggesting that the upregulation of miR‐210 in synovial fluid may occur in the early stage of OA and can serve as a useful biomarker for the early diagnosis of OA. In addition, miR‐210 is positively correlated with the level of vascular endothelial growth factor (‌VEGF), indicating that miR‐210 may promote the development of OA by promoting ‌VEGF expression and angiogenesis. Chen et al. screened out nine differential miRNAs from extracellular vesicles in synovial fluid of healthy individuals and OA patients using the GSE126677 dataset and verified them in synovial fluid of patients with early and advanced KOA. Finally, two upregulated miRNAs (miR‐130b‐3p and miR‐1271‐5p) and two downregulated miRNAs (miR‐3126‐5p and miR‐3976) were screened out [293]. miR‐130‐3p regulates macrophage polarization and inflammatory responses, while silencing miR‐1271‐5p inhibits cartilage damage in OA [293]. These data suggest that miR‐130b‐3p and miR‐1271‐5p may play a key role in the OA microenvironment and serve as detection markers for OA. In addition, the expression of miR‐182‐5p in extracellular vesicles of synovial fluid in patients with OA is significantly reduced, correlating with inflammatory indicators. miR‐182‐5p derived from exosomes derived from joint synovial fluid downregulates TNFAIP8 and promotes autophagy to alleviate OA [294].

Gender‐specific miRNAs explain sex differences in OA prevalence. The prevalence of OA is higher in women than in men, and this risk increases after menopause. Kohle et al. identified 45 upregulated/69 downregulated miRNAs in male OA synovial extracellular vesicles and 53 upregulated/91 downregulated in females [295], indicating that there is gender‐specific differences in the content of miRNA in extracellular vesicles of OA cells. Among them, miR‐504‐3p is the only miRNA that is upregulated in both genders. Female‐specific miRNAs are estrogen‐reactive and target the TLR signaling pathway, partially explaining higher OA prevalence in women [295].

Jamshidi developed a miRNA prognostic model using integrated machine/deep learning tools to identify adventitious/nonadventitious knee joint OA structures. Their study included 152 participants, including 91 progressiers and 61 nonprogressiers, and used seven machine learning tools to identify key miRNAs and OA influencing factors (age, gender, BMI, race). The optimized prediction model based on artificial neural network screened out four miRNAs (hsa‐miR‐556‐3p, hsa‐miR‐3157‐5p, hsa‐miR‐200a‐5p, hsa‐miR‐141‐3p) with the potential to predict the progression of OA, which is expected to provide assistance for future personalized treatment monitoring [296].

miRNAs in serum and synovial fluid (including extracellular vesicles) exhibit diverse roles in OA pathogenesis, with robust potential for noninvasive diagnosis, subtype differentiation, severity grading, treatment response assessment, and personalized prognosis. While individual miRNAs show promise, future studies should validate multi‐miRNA panels and integrate them with clinical/imaging data to enhance clinical utility.

#### 3.2.5 Metabolic Biomarker

Systemic metabolic disorders play a significant role in the pathogenesis of OA [297]. Identifying characteristic molecular metabolites prior to irreversible joint degeneration is critical for optimizing clinical diagnostic decisions and delaying OA progression [298]. Lin et al. investigated OA metabolic biomarkers via serum metabolomics analysis using ultra‐performance liquid chromatography–tandem quadrupole time‐of‐flight mass spectrometry, combined with multivariate pattern recognition and univariate statistical methods [299]. Their results identified five key metabolites for distinguishing OA from healthy controls: phosphatidylcholine (18:0/22:6), p‐cresol sulfate (p‐CS), 12‐hydroperoxy‐5,8,10,14‐eicosatetraenoic acid, cis‐vaccenic acid, and perfluorooctane sulfonic acid. Additionally, phosphatidylcholine (18:0/18:0) and phenylalanine emerged as markers for differentiating OA from other arthritic disorders [299].

OA is characterized by metabolic pathway dysfunction, notably acetyl‐CoA accumulation. Enhanced CoA‐catalyzed glycolysis contributes to cartilage degradation and joint pain. For instance, in senescent chondrocytes with Acot12 knockout (A12KO) and NudT7 knockout (N7KO), distinct changes in acetyl‐CoA and CoA concentrations are hallmark features of OA and cartilage degeneration, supporting CoA as a potential marker for early OA diagnosis and progression monitoring [300, 301, 302]. However, conventional CoA detection methods (liquid chromatography, colorimetry, fluorescence) suffer from instability, low sensitivity, and complex procedures. To address this, Robby et al. developed a CoA‐sensitive electrochemical nanogel sensor. CoA abundance in OA triggers the breakdown of polyallylamine–manganese oxide–polymer‐coated MnO_2_ on the sensor electrode, altering conductivity and fluorescence [303]. Integration of this sensor with wireless monitoring devices enables direct transmission of sensing data to smartphones, highlighting its potential for noninvasive early OA diagnosis and prognostic assessment.

Metabolomics studies have identified specific serum metabolites for OA diagnosis and differential diagnosis, while CoA serves as a functionally relevant metabolic marker; novel electrochemical sensors address limitations of traditional CoA detection, facilitating clinical translation for early OA assessment.

#### 3.2.6 Others Biomarkers

Other promising OA biomarkers involve in hypoxia response, oxidative stress, inflammatory–nutritional balance, and tissue remodeling.

OA is characterized by chondrocytes degeneration and bone hyperplasia, which can cause hypoxia of synovial fluid. HIF‐1α, a transcription factor that promotes gene transcription and chondrocyte survival under hypoxic conditions, has emerged as a potential OA biomarker [304, 305, 306]. Chu et al. conducted a cross‐sectional study on 278 KOA patients and 203 healthy controls, and found that HIF‐1α level in the synovial fluid of OA patients was significantly higher than that of the control group [307]. Serum and synovial fluid HIF‐1α levels correlated with disease severity and radiological progression, supporting its utility as a prognostic marker for KOA [307]. Qing et al. further confirmed elevated synovial fluid HIF‐1α in OA patients compared with the healthy control, with levels correlating with disease severity and articular cartilage HIF‐1α expression [308]. These findings collectively link synovial fluid and articular cartilage HIF‐1α to joint injury progression, positioning it as a potential marker for OA severity assessment and progression prediction.

NADPH oxidase (NOX) generates reactive oxygen species (ROS) and oxidative stress induces chondrocyte apoptosis. Therefore, NOX may be involved in the pathogenesis of OA [309, 310]. The cross‐sectional study conducted by Kim et al. confirmed significantly increased serum age‐related NOX (arNOX) activity in severe OA patients, consistent with arNOX changes in cartilage, indicating arNOX overactivity correlates with cartilage degeneration and OA severity [311]. Subsequent animal experiments validated NOX's pathogenic role. Upregulating NOX expression accelerates cartilage degeneration during the process of OA, while inhibiting NOX activity can delay the progression of OA [312, 313, 314, 315]. However, clinical research on NOX activity in body fluids remains limited, necessitating further studies to validate its potential as an OA clinical marker.

The CRP–albumin–lymphocyte index (CALLYI) is an inflammatory nutritional indicator proposed by Iida et al. It is calculated as albumin × lymphocyte count ÷ (CRP × 10), integrating assessments of nutritional status, immune function, and inflammatory activity [316]. Clinical studies have shown that CALLYI is practical in predicting the correlation and prognosis of various diseases, especially cancer [317, 318, 319, 320, 321]. Geng et al. constructed three weighted multiple regression models to study the correlation between CALLYI and OA in the National Health and Nutrition Examination Survey (NHANES) samples [322]. Multivariate logistic regression models revealed a negative correlation between higher CALLYI and OA. Restricted cubic splines (RCS) confirmed a significant nonlinear relationship with OA prevalence, and decision curve analysis validated clinical utility [322]. This study the first to establish a nonlinear negative correlation between CALLYI and OA using NHANES data, supports CALLYI as a potential biomarker for OA risk prediction. However, larger multicenter prospective cohort studies are needed to address limitations of the cross‐sectional design.

Bradykinin is an endogenous proinflammatory factor and, has been linked to OA. Bradykinin receptor B1 and B2 antagonists improve pain tolerance and alleviate OA symptoms [323, 324]. Belluci et al. found synovial fluid bradykinin levels in KOA patients correlated positively with cartilage degradation and inflammatory marker concentrations [325]. Notably, oral hyaluronic acid (HA) not only improves pain and function in obese KOA patients but also reduces synovial fluid bradykinin levels [326], further supporting bradykinin's role as a functional marker of OA inflammation and treatment response.

Thrombospondin‐4 (TSP‐4), a secretory glycoprotein belonging to the thrombospondin family, regulates tissue repair, inflammation, and disease progression via modulating cell adhesion, proliferation, and ECM remodeling [327]. In human synovial tissue, TSP‐4 levels are positively correlated with the degree of tissue fibrosis [328]. PCR, immunohistochemistry, and Western blotting analyses confirmed increased TSP‐4 expression in OA, with levels correlating with disease severity [329]. Abundant TSP‐4 specific degradation products could also be detected in the serum of OA patients, revealing that TSP‐4 degradation products may serve as OA‐specific biomarkers [329]. In female OA patients, synovial fluid TSP‐4 concentrations correlated with pain intensity. Further analysis of synovial fluid and serum samples from larger cohorts is required to validate TSP‐4 as a biomarker for OA‐related pain.

HIF‐1α, NOX, CALLYI, bradykinin, and TSP‐4 reflect distinct OA pathogenic pathways such as hypoxia, oxidative stress, inflammatory–nutritional balance, inflammation, and tissue remodeling. While preclinical and preliminary clinical evidence supports their potential, larger‐scale, standardized studies are needed to confirm their clinical utility for OA diagnosis, prognosis, and treatment monitoring.

## 4 Clinical Conservative Treatments of OA

Owing to the limited regenerative capacity of articular cartilage, irreversible disease progression, and complex heterogeneous etiologies, current medical interventions primarily aim to alleviate symptoms and delay disease advancement rather than achieve complete repair of damaged tissues or elimination of all pathogenic factors. The core therapeutic goals of OA are to relieve pain, preserve or improve joint function, and protect residual joint structures. As the first‐line management strategy for OA, conservative treatment is mainly applied in the early or mild stage of the disease, following a stage‐specific and individualized approach. It focuses on restorative and symptomatic relief measures, integrating pharmacological and nonpharmacological interventions to slow down disease progression and avoid or delay the need for invasive treatments. Specifically, conservative treatment includes cartilage repair drugs and physical therapy for analgesic and functional improvement, all of which are safe, accessible, and tailored to patients with mild to moderate OA.

Clinical management follows a stage‐specific approach, integrating standardized protocols with individualized adjustments based on disease severity. In the early or mild stage of OA, clinical intervention mainly focuses on restorative treatment, such as cartilage repair drugs and joint injections (hormone drugs, platelet‐rich plasma [PRP], stem cells). At the moderate stage, analgesic treatment is the main approach, such as nonsteroidal anti‐inflammatory drug therapy, minimally invasive interventional treatment (radiofrequency and joint cavity lavage), physical therapy, joint debridement, and osteotomy. Patients with severe OA mainly receive reconstructive treatment such as TKA as the gold‐standard surgical option.

### 4.1 Pharmaceutical Treatment of OA

#### 4.1.1 Drugs in Clinical Use

Clinically approved OA drugs primarily target symptom relief and functional improvement, with distinct roles based on disease stage and patient comorbidities. Nonsteroidal anti‐inflammatory drugs (NSAIDs) are the first‐line pharmacotherapy for pain and inflammation, including oral formulations ibuprofen, naproxen, and celecoxib and topical preparations diclofenac gel and ketoprofen patch. Topical NSAIDs are preferred for localized symptoms to minimize systemic adverse effects (gastrointestinal irritation, cardiovascular risks) associated with oral administration. For patients with NSAID contraindications or refractory pain, weak opioids tramadol may be prescribed short‐term, though long‐term use is limited by risks of dependence and adverse effects. Intra‐articular injectables play a key role in localized intervention. Glucocorticoids triamcinolone acetonide provide rapid anti‐inflammatory effects for acute symptom flares, while HA enhances joint lubrication, reduces friction, and alleviates pain in mild‐to‐moderate OA. Additionally, oral glucosamine sulfate (GAS) and chondroitin sulfate, are widely used to slow cartilage degradation, though their efficacy remains variable across patients.

#### 4.1.2 Drugs in Clinical Trials

Given the critical unmet need for DMOADs and the limitations of current symptom‐focused therapies, ongoing clinical trials are advancing a diverse pipeline of targeted agents and cell‐based therapies, spanning inflammation modulation, cartilage repair, bone metabolism regulation, pain management, and tissue regeneration (Table 2).

**TABLE 2:** Ongoing pharmacological interventions trials for osteoarthritis.

Condition	Intervention	ClinicalTrials ID	Phase	Enrollment	Key findings	References
Osteoarthritis, knee osteoarthritis	Diacerein, celecoxib	NCT02688400, DAR‐INT‐14‐01, 2015‐002933‐23	Phase III	380	Diacerein is not inferior to celecoxib in alleviating pain, improving physical function, and has good safety.	[330]
Osteoarthritis	LY3857210	NCT05620563, 18388, H0P‐MC‐OA05	Phase II	147	LY3857210 reduces pain, improves the Western Ontario and McMaster Universities Arthritis Index (WOMAC) and quality‐of‐life scores.	
Osteoarthritis	LY3016859	NCT04456686, 17513, H0P‐MC‐OA01	Phase II	117	Fepixnebart (LY3016859) relieves pain and improves WOMAC scores.	[331]
Osteoarthritis, knee osteoarthritis	LY3526318	NCT05080660, 17592, H0P‐MC‐OA02	Phase II	160	LY3526318 shows pain improvement trend but no significant difference compared with placebo.	
Knee osteoarthritis	SM04690	NCT03570554, SM04690‐OA‐04	Phase II	700	Adavivint (SM04690) effectively reduces pain and improves functional scores.	
Osteoarthritis	SM04690	NCT02536833, SM04690‐OA‐02	Phase II	455	SM04690 is generally safe and well‐tolerated, with significant improvements in WOMAC pain scores.	[332, 333, 334]
Osteoarthritis	SM04690	NCT02951026, SM04690‐OA‐05	Phase II Phase III	703	A single SM04690 injection sustains improvements in WOMAC pain and function for at least 12 months.	[332, 333, 334]
Osteoarthritis	Ropivacaine, methylprednisolone	NCT02576249, 15–003120	Phase IV	29	Ropivacaine plus methylprednisolone improves pain scores, with better efficacy than methylprednisolone alone.	
Osteoarthritis	GLPG1972	NCT03595618, GLPG1972‐CL‐201, 2017‐004581‐10, U1111‐1205‐0321	Phase II	932	GLPG1972 (S201086) does not significantly reduce cartilage loss or improve symptoms in symptomatic knee osteoarthritis adults.	[335, 336, 337]
Osteoarthritis	Theraflex (BAY 874017)	NCT03330288, 19649	Phase IV	1102	BAY 874017 improves pain and joint function, and enhances quality‐of‐life scores.	
Osteoarthritis	Otilimab (GSK3196165)	NCT02683785, 204851, 2015‐003089‐96	Phase II	44	GSK3196165 shows no significant difference from placebo in improving joint swelling and pain, with no serious adverse events.	[338]
Knee osteoarthritis, hip osteoarthritis	MT‐5547	NCT03245008, MT‐5547‐J01	Phase II Phase III	626	MT‐5547 significantly improves the WOMAC pain and physical function score.	
Hip osteoarthritis, knee osteoarthritis	Fasinumab	NCT02683239, R475‐PN‐1523, 2015‐003783‐36	Phase III	5331	Fasinumab has good long‐term tolerance and no serious adverse reactions compared with placebo.	[339]
Knee osteoarthritis, hip osteoarthritis	Fasinumab, diclofenac, celecoxib	NCT03304379, R475‐OA‐1688, 2017‐001702‐15	Phase III	1650	Fasinumab significantly improves WOMAC pain and physical function scores within 24 weeks.	[340]
Knee osteoarthritis, hip osteoarthritis	Fasinumab, naproxen	NCT03161093, R475‐OA‐1611, 2016‐005020‐29	Phase III	3307	Fasinumab improves WOMAC pain and physical function scores.	[340]
Knee osteoarthritis, hip osteoarthritis	Fasinumab	NCT03691974, R475‐OA‐1758, 2017‐004921‐33	Phase II	180	Fasinumab increases peroneal motor nerve conduction velocity and action potential amplitude, significantly alleviates pain, and improves motor ability.	
Knee osteoarthritis	FX006 (Zilretta), triamcinolone acetonide	NCT02637323, FX006‐2015‐009	Phase II	81	FX006 achieves sustained release for up to 20 weeks after a single 32 mg/5 mL intra‐articular injection.	
Knee osteoarthritis	FX006, triamcinolone acetonide	NCT02762370, FX006‐2015‐010	Phase II	33	FX006 minimizes blood glucose fluctuations in patients with Type 2 diabetes.	[341]
Knee osteoarthritis	FX006, triamcinolone acetonide	NCT03046446, FX006‐2016‐011	Phase III	208	Repeated FX006 administration is well‐tolerated and improves WOMAC pain, stiffness, and function scores.	[342]
Bilateral knee osteoarthritis	FX006, triamcinolone acetonide	NCT03378076, FX006‐2017‐012	Phase II	24	Bilateral intra‐articular FX006 injection is well‐tolerated, with lower peak plasma betamethasone concentration than bilateral triamcinolone acetonide injection.	[343]
Knee osteoarthritis	FX006, triamcinolone acetonide	NCT03529942, FX006‐2017‐014	Phase III	129	A single intra‐articular FX006 injection reduces synovial volume.	
Hip osteoarthritis	FX006, triamcinolone acetonide	NCT04129944, FX006‐2019‐017	Phase III	16	FX006 improves pain while maintaining stable blood glucose levels.	
Knee osteoarthritis	FX006	NCT03895840, STUDY00142926	Phase IV	70	Bilateral intra‐articular FX006 injection improves patient motor ability.	
Knee osteoarthritis	Capsaicin	NCT03124407, VZU‐00022	Phase IV	120	Capsaicin topical solution significantly increases the percentage of subjects with ≥50% pain reduction at 24 h, 8d, and 28d.	[344]
Knee osteoarthritis	KA34	NCT03133676, CBR‐KA34‐3001	Phase I	60	KA34 is well tolerated with no serious adverse reactions.	
Knee osteoarthritis	AMZ001 (3.06% diclofenac gel)	NCT03691844	Phase II Phase III	444	AMZ001 significantly alleviates pain.	
Knee osteoarthritis	CNTX‐4975	NCT03660943, CNTX‐4975i‐OA‐301, CNTX‐4975i‐OA‐304	Phase III	332	CNTX‐4975 effectively alleviates pain in patients with chronic moderate‐to‐severe knee osteoarthritis.	
Knee osteoarthritis	4P‐004 (liraglutide)	NCT05419856	Phase I	34	Single‐dose intra‐articular 4P‐004 is safe and well‐tolerated.	
Knee osteoarthritis	Cingal, triamcinolone hexacetonide	NCT04231318, Cingal 19‐01	Phase III	231	Cingal significantly improves WOMAC pain scores.	
Knee osteoarthritis	Colchicine	NCT03913442, 16–01796	Phase IV	120	Colchicine improves visual analog scale (VAS) pain scores, Knee Injury and Osteoarthritis Outcome Score (KOOS) pain scores, stiffness scores, and physical function scores.	
Knee osteoarthritis	TLC599	NCT03005873, TLC599A2003	Phase II	76	TLC599 is well tolerated and relieves pain/improves function for up to 24 weeks at 12 mg.	[345]
Knee osteoarthritis	TLC599, dexamethasone sodium phosphate (DSP)	NCT04123561, TLC599A3005	Phase III	504	TLC599 outperforms nonliposomal DSP in reducing average daily pain (ADP). 52‐week follow‐up shows multiple uses better improve ADP and WOMAC pain scores.	
Hip osteoarthritis, knee osteoarthritis	CR845	NCT02944448, CR845‐CLIN2002‐PO	Phase II	761	CR845 reduces joint pain at 5.0 mg but shows no significant difference compared with placebo.	
Chronic pain, hip osteoarthritis, Knee osteoarthritis	Tanezumab, naproxen, celecoxib, diclofenac	NCT02528188, A4091058, 2012‐003721‐22	Phase II	3021	Tanezumab and NSAIDs provide early and sustained efficacy up to 56 weeks.	[346, 347]
Knee osteoarthritis, hip osteoarthritis	Tanezumab	NCT02697773, A4091056, 2013‐002222‐23	Phase III	698	Subcutaneous tanezumab significantly improves pain, physical function assessment scores, and Patient Global Assessment of Osteoarthritis scores in patients with moderate‐to‐severe knee/hip osteoarthritis.	[348, 349]
Hip osteoarthritis, knee osteoarthritis	Tanezumab	NCT02709486, A4091057, 2013‐004508‐21	Phase III	849	Subcutaneous tanezumab reduces pain within the first week and improves pain/function within 24 weeks.	[350, 351]
Knee osteoarthritis	EP‐104IAR	NCT04120402, EP‐104IAR‐201	Phase II	318	EP‐104IAR relieves OA pain for up to 14 weeks with a single injection.	[352]
Knee osteoarthritis	MIV‐711	NCT02705625, MIV‐711‐201	Phase II	244	MIV‐711 increases cartilage thickness and improves WOMAC pain and stiffness scores.	
Knee osteoarthritis	MIV‐711	NCT03037489, MIV‐711‐202	Phase II	50	MIV‐711 is well tolerated with no serious adverse reactions.	
Knee osteoarthritis	Diclofenac sodium gel, 1%, Voltaren Gel	NCT02596451, NCT03172780, NCT02913521, GLK‐1402, P130021, MYL‐1601N‐3002	Phase III	3318	Diclofenac sodium gel and Voltaren Gel have clinical bioequivalence in treating knee osteoarthritis.	
Hand osteoarthritis	Denosumab (AMC‐162)	NCT02771860, AGO/2015/008, 2015‐003223‐53	Phase II	100	Denosumab exerts significant structural modification effects compared with the placebo, reducing erosion progression at 24 weeks and enhancing efficacy at 48 weeks.	
Knee osteoarthritis	MM‐II	NCT04506463	Phase II	397	MM‐II is well‐tolerated, with an optimal effective dose of 3 mL for knee osteoarthritis pain.	[353]
Knee osteoarthritis	CGS‐200‐1 (1% capsaicin), CGS‐200‐5 (5% capsaicin)	NCT03528369, VZU00025	Phase II	122	CGS‐200 significantly alleviates pain and improves WOMAC pain, stiffness, and function scores.	
Knee osteoarthritis	Sustained acoustic device with 2.5% diclofenac patch, 1% diclofenac topical gel	NCT0505044, HF‐01‐2021	Phase I Phase II	60	Compared with 1% diclofenac topical gel, the sustained acoustic device + 2.5% diclofenac patch (5 days/week for 8 weeks) significantly reduces WOMAC scores with no time dependence.	
Knee osteoarthritis	Semaglutide	NCT05064735	Phase III	407	Weekly semaglutide significantly improves weight and joint pain in obese patients with moderate‐to‐severe knee osteoarthritis.	[9]
Knee osteoarthritis	GSK3858279	NCT03485365, 207804, 2017‐004809‐41	Phase I	97	GSK3858279 significantly improves average/worst knee pain, and WOMAC pain/function scores, with good safety and tolerability.	
Osteoarthritis	Bone marrow derived MSCs, adipose‐derived MSCs, umbilical cord tissue MSCs, Corticosteroid	NCT03818737, IRB00108046	Phase II	475	One year after injection, the three biological therapies are not superior to corticosteroid injections in terms of KOOS and VAS pain scores, with no significant differences among three therapies.	[354, 355, 356]
Knee Osteoarthritis	Lipogems micro‐fragmented adipose tissue	NCT03714659	Phase II	20	Ultrasound‐guided direct injection of micro‐fragmented adipose tissue into the knee joint improves Numeric Pain Scale (NPS) and KOOS scores in knee osteoarthritis patients.	[357]

Neurotrophic factor inhibitors remain a focal point for refractory OA pain. Fasinumab and tanezumab, both monoclonal antibodies targeting NGF, are being evaluated in late‐stage trials for moderate‐to‐severe OA pain, offering an alternative to opioids and NSAIDs by blocking pain signaling pathways [339]. CNTX‐4975‐05, a synthetic trans‐capsaicin analog targeting transient receptor potential vanilloid 1 channel, is undergoing trials for localized pain relief, leveraging its ability to desensitize nociceptive neurons without systemic adverse effects.

Several small‐molecule drugs aim to halt or reverse cartilage degradation. SM04690, a Wnt signaling pathway modulator, is being tested in Phase III trials for KOA, as it suppresses catabolic pathways and promotes chondrocyte survival to preserve cartilage integrity [332, 333, 334]. LY3857210, LY3556050, and LY3016859 are under investigation for their roles in regulating cartilage metabolism, with preliminary data suggesting they inhibit MMPs or enhance anabolic pathways to slow JSN [331]. MT‐5547, a selective inhibitor of aggrecanase (ADAMTS‐4/5), targets the breakdown of cartilage proteoglycans, a key driver of OA progression, and is currently in mid‐stage trials.

Agents targeting subchondral bone remodeling, a critical contributor to OA pathogenesis, include denosumab, a monoclonal antibody against receptor activator of nuclear factor kappa‐B ligand (RANKL), which inhibits osteoclast activity to reduce subchondral bone sclerosis and inflammation, and trials are exploring its efficacy in slowing structural progression in KOA. MIV‐711, a selective cathepsin K inhibitor, regulates bone resorption and collagen degradation, with trials evaluating its potential to protect both cartilage and subchondral bone in OA.

Localized interventions aim to enhance bioavailability while minimizing systemic effects. FX006, a long‐acting triamcinolone acetonide sustained‐release, is being tested in Phase III trials for KOA, designed to prolong joint retention and improve lubrication beyond conventional triamcinolone acetonide injections [341].

MSC‐based therapies are emerging as promising regenerative strategies, leveraging their immunomodulatory and trophic effects to promote tissue repair: bone marrow‐derived MSCs, adipose‐derived MSCs, and umbilical cord tissue‐derived MSCs are all under evaluation in clinical trials for mild‐to‐moderate OA [354, 355, 356]. These cell products target local inflammation, secrete paracrine factors that stimulate cartilage regeneration, and modulate immune cell activity, and trials are focusing on optimizing dosing, delivery routes and patient stratification to maximize therapeutic efficacy.

Collectively, these clinical trial candidates represent a paradigm shift from symptomatic management to disease modification, with the potential to address OA's heterogeneous pathogenesis and improve long‐term joint health outcomes.

#### 4.1.3 Preclinical Drugs

Traditional therapeutic drugs for OA are plagued by limitations such as poor stability, short half‐life, low bioavailability, and high systemic toxicity, restricting their clinical efficacy. Nanocarrier systems, characterized by large specific surface areas and modifiable surfaces, address these drawbacks by enhancing drug‐loading capacity, enabling targeted delivery to specific tissues/organs, prolonging in vivo circulation, improving stability, and increasing bioavailability [358, 359, 360]. These advantages have driven extensive research into nanoscale drug delivery systems for OA therapy, where they enhance therapeutic effects while minimizing off‐target side effects and systemic toxicity compared with free drug molecules.

##### 4.1.3.1 Nanogels

Hydrogels are hydrophilic 3D network structure gel that rapidly swell in water while maintaining structural integrity. As a new type of biomaterial, hydrogels mainly improve OA symptoms through lubricating joints, delivering drugs slowly, or promoting cartilage repair, and also serve as drug carriers or tissue engineering scaffolds. Nanogels are polymeric hydrogel polymers formed through physical or chemical cross‐linking, exhibit hydrophilicity or amphiphilicity and have a strong water retention capacity [361]. Nanogels combine the advantages of hydrogels and nanoparticles (NPs). They not only have excellent biocompatibility and biodegradability, but also feature high drug loading capacity, strong permeability, and slow release. These characteristics make it easier for the nanogels to reach the damaged areas and prolong the retention time of the drugs in the joints [362]. A multifunctional anti‐inflammatory drug (CPHs) of a peptide dendritic nanogel specifically targets activated macrophages through folic acid (FA)‐modified HA. By rapidly releasing a large amount of carbon monoxide (CO), the activation of the p38 MAPK and NF‐kB pathways and the secretion of their downstream IL‐1β, IL‐6, and TNF‐α were inhibited in vitro. CPHs could significantly clear ROS in the OA joint, and effectively inhibit the degradation of articular cartilage and its ECM in vivo [363]. Li et al. prepared glycerol chitosan (GC)/fucoidan nanogels loaded with anti‐inflammatory peptide KAFAK (GC/Fu@KAFAK NGs) based on chlorogenic acid cross‐linking method and electrostatic interaction [364]. GC/Fu@KAFAK NGs demonstrated excellent biocompatibility via enhancing the expression of cartilage marker such as SRY‐box transcription factor 9 (SOX ‐ 9), Type II collagen, and aggrecan, and inhibiting the secretion of inflammatory factors [364]. Nedunchezian et al. combined HA methacrylate (HAMA)/collagen methacrylate (GelMA) with acrylic functionalized nano‐silica (AFnSi) crosslinking agent to construct a hybrid hydrogel capable of loading adipose‐derived stem cells (ADSCs), which not only supports the differentiation of ADSCs, but also significantly increase the expression of SOX‐9, aggrecan, and Type II collagen [365].

Advanced nanogel designs further optimize targeting and phase‐specific effects. Artificial M2 macrophages (AM2M), composed of a macrophage membranes “shell” and inflammation‐responsive nanogels “yolk,” achieves pulsed release during the acute inflammatory phase to downregulate inflammation and continuous release during the low inflammatory activity phase to repair cartilage via the ionic and hydrogen bonds between collagen and chondroitin sulfate. Furthermore, AM2M exhibits targeting and long‐term retention in the inflammatory area and prevents the immune stimulation of macrophages by chondroitin sulfate [366]. The injectable nanogel (RGD‐Nanogel/siRNA Cd61) developed by Dai et al. provided the continuous release of siRNA Cd61 targeting integrins. RGD‐Nanogel/siRNA Cd61 demonstrated excellent biocompatibility and targeting ability and alleviated cartilage degeneration in OA mice via localized injection [367]. The complex microenvironment and limited space of the joints lead to the rapid clearance of drugs. Sun et al. functionalized kartogenin (KGN)‐loaded zeolitic imidazolate framework‐8 with HA (KZIF@HA) [368]. KZIF@HA released KGN continuously for 1 month in the OA microenvironment, enhanced cartilage permeability, promoted hyaline cartilage regeneration via ECM secretion, and modulated JNK/extracellular signal‐regulated kinase (ERK) pathways and M2 macrophage polarization [368]. These results show that the nanogels system constructed by combining nanogels with various functional components can achieve the treatment of OA by reducing inflammation, promoting cartilage regeneration or improving joint function.

Functionalized nanogels also leverage environmental responsiveness to alleviate inflammation and the generation of catabolic markers by loading type an endothelin receptor antagonist (BQ‐123‐CHI) and type B1 retarder receptor antagonist (R‐954‐HA), and have good tolerance [369, 370]. Temperature‐responsive poly (N‐vinyl caprolactone) nanogels can be used for the delivery of local anti‐inflammatory drugs such as diclofenac [371]. pH/REDOX responsive nanogels regulated geraniol release and effectively inhibited the degradation of cartilage matrix by increasing the levels of nuclear factor erythroid 2‐related factor 2 (Nrf2) and heme oxygenase‐1 (HO ‐ 1) [372]. Another inflammation‐responsive nanogel, LDH@TAGel, protected chondrocytes from inflammatory, oxidative stress, and apoptosis through Nrf2/Keap1 and PI3K/AKT pathways [373]. TAGel directly cleared ROS and showed antioxidant properties, while the loaded Layered double hydroxide (LDH) could restore ECM secretion function of damaged chondrocytes and downregulate the expression of MMPs, thereby alleviating ACLT‐induced cartilage degeneration and damage [373].

Due to its biocompatibility and ability to support cell growth and encapsulate stem cells to differentiate into chondrocytes, HA performs well in providing growth factor binding sites and can be considered capable of serving as an ideal intra‐articular delivery system. Nah et al. designed o‐phenylene diamine (o‐PD) derivatives with good nitric oxide (NO)‐scavenging capability (NSc), which self‐assemble with HA into NPs (HA‐NSc NPs) under water conditions. HA‐NSc NPs effectively reduced intracellular NO concentration and inflammatory cytokines, significantly alleviated pain and reduced cartilage damage [374]. Compared with free high molecular weight HA, self‐assembled HA‐NPs was resistant to hyaluronidase digestion in vitro and exhibited longer knee joint retention ability in vivo. IAI of HA‐NPs inhibited CD44‐mediated NF‐κB activation in chondrocytes, protecting articular cartilage in the OA mouse model [375].

HA‐based hydrogels are widely used to deliver cells and biomolecules to OA joints, with modified platforms enhancing joint retention and controlled release. Jahanbekam et al. designed an injectable, ultrasound‐triggered thermosensitive hydrogel (pluronic F‐127/HA/gelatin) loaded with hydrocortisone, which showed unique thermal response characteristics, prolonged drug release, and had the potential to treat OA [376]. Additionally, an HA/PRP hydrogel (HA/PRP/BM) containing MnO_2_ nanozyme significantly inhibited the degradation of cartilage matrix. Through the synergistic effects of mechanical dissipation, inflammation suppression, and cartilage repair promotion, HA/PRP/BM suppressed the development of OA and thus holds significant application prospects in the treatment of OA [377]. HA‐based nanomaterials, leveraging HA's inherent biocompatibility and modifiability, enhance drug retention, regulate inflammation, and promote repair, emerging as versatile platforms for OA preclinical therapy.

Ozone (O_3_) exhibits strong anti‐inflammatory properties but suffers from high reactivity and short half‐life, limiting its OA therapeutic efficacy. Wu et al. addressed this with an O_3_‐enriched thermosensitive nanocomposite hydrogel (O_3_ NPs@MHPCH) [378]. In this hydrogel, O_3_ is encapsulated in NPs composed of perfluorobutylamine and fluorinated HA to enhance its stability. O3 NPs@MHPCH treatment significantly reduced VEGF and inflammatory levels, decreased the levels of inflammatory factors such as IL‐1β, IL‐6, and TNF‐α in vitro, thereby promoting the expression of Type II collagen and chondroitin and stimulating chondrocyte proliferation. Additionally, O3 NPs@MHPCH significantly alleviated OA by reducing synovial inflammation, cartilage destruction, and subchondral bone remodeling in vivo, providing a promising option for improving the stability of O_3_ and enhancing the efficacy of O_3_ therapy.

The combination of nanomaterials and near‐infrared light shows great potential for wide application in the field of OA treatment. Wang et al. evaluated MXene hydrogels (Mo_2_Ti_2_C_3_‐loaded chitosan hydrogels) with near‐infrared (NIR) irradiation (808 nm laser) in mice. The combined treatment reduced the expression of M1 macrophages‐associated inflammatory factors (IL‐1β and IL‐6) in the cartilage, increased the expression of M2 macrophages‐associated transforming growth factor (TGF)‐β and IL‐10, increased glycosaminoglycans (GAGs), aggrecan, and Type II collagen, thereby mitigating cartilage damage and osteophytes formation, and delaying OA progression [379]. MXene hydrogels may provide a new strategy for the treatment of OA.

Excessive ROS in joints can lead to the degradation of ECM and the apoptosis of chondrocytes, thereby promoting the occurrence and development of OA. Polydopamine (PDA) NPs loaded with ultra‐small palladium (PDA‐Pd NPs) effectively reduced intracellular ROS levels and exhibited efficient antioxidant and anti‐inflammatory capabilities as well as good biocompatibility in chondrocytes stimulated by IL‐1β [380]. Notably, with the assistance of NIR irradiation, its therapeutic effect has been further enhanced [380]. Mitochondrial ROS (mROS) play a crucial role in the development of OA and may be a promising therapeutic target. Li et al. developed an NIR‐responsive nanozyme (Mn_3_O_4_@PDA@Pd‐SS31) targeting mROS [381]. Nanozyme could accelerate the release of Pd and Mn_3_O_4_ under NIR irradiation, demonstrating enhanced superoxide dismutase (SOD)‐like and catalase‐like enzyme activities, thereby effectively reversing mitochondrial dysfunction and promoting mitochondrial autophagy, ultimately eliminating mROS and inhibiting the inflammatory response in chondrocytes. In vivo experiments confirmed that Mn_3_O_4_@PDA@Pd‐SS31 is excreted through the intestinal metabolic pathway, has good biocompatibility, effectively reduces inflammatory responses, and alleviates the degeneration of articular cartilage in OA joints, which holds great promise for the clinical treatment of OA. NIR‐responsive nanomaterials enhance anti‐inflammatory and cartilage‐protective effects via spatiotemporal control, offering a novel strategy for targeted OA therapy. HA‐based nanogels, O_3_‐enriched hydrogels, and NIR‐responsive nanomaterials address limitations of traditional drugs by enabling targeted, sustained delivery and multimodal action (anti‐inflammation, ROS scavenging, cartilage regeneration).

Despite these promising preclinical results, no nanogel‐based drugs have entered clinical use, requiring further research into biocompatibility, scalability, and long‐term safety to validate efficacy, safety, and clinical translation potential [382, 383].

##### 4.1.3.2 Liposomes

Liposomes are spherical vesicles composed of phospholipid bilayers, uniquely suited for lipophilic drug delivery. By fusing with recipient cell membranes, they concentrate drugs at lesion sites, enhance the stability of the drug, improve the therapeutic effect of the drug, and minimize toxicity [384].

Liposomes loaded with the anti‐inflammatory drug GAS achieved sustained drug release while providing joint lubrication; they could accelerate primary mouse chondrocyte proliferation and exert anti‐inflammatory and chondroprotective effects by downregulating pain‐related genes, proinflammatory cytokines, and catabolic proteases [385]. Resolvins are a type of highly efficient molecules that play a significant role in inflammation resolution and tissue repair. However, the use of resolvin D1 is limited by rapid lymphatic clearance or oxidative inactivation. Nanopoliposomes loaded with RvD1 (Lipo‐RvD1) have a good sustained‐release effect and improve cartilage injury in OA mice induced by surgery by weakening the proinflammatory activity of synovial macrophages and recruiting more M2 macrophages at the inflammatory site [386]. Lipo‐RvD1 also has an improving effect on obesity‐induced OA via targeting macrophage infiltration in synovial tissue [387, 388].

NSAIDs and glucocorticoids benefit from liposomal delivery. Meloxicam (MLX), a clinically widely used NSAID with poor solubility and high lipophilicity, was formulated into MLX‐Ca(AC)_2_Lipo using dexglucose anhydride to enhance water solubility and Ca^2^^+^ to increase encapsulation efficiency to 98.4% [389]. MLX‐Ca(AC)2 Lipo was stable in nature, which not only prolonged the duration of drug efficacy but also improved bioavailability [389]. IAI of MLX‐Ca(AC)2 Lipo increased lubrication, protected cartilage from progressive wear, and downregulated the synthesis of prostaglandin E2, thereby reducing chondrocyte apoptosis and ECM degeneration [389]. Dexamethasone liposome alleviated OA pain by promoting macrophage M2 polarization and inhibiting inflammatory cytokine expression [390]. Liposomes loaded with triamcinolone acetonide (TA) and camouflaged neutrophil membranes (TA‐NM@Lip) reduced the levels of chemokines, proinflammatory factors and chondrodegrading enzymes in the joints of rodents associated with OA pain, while a single IAI of TA‐NM@Lip provides long‐lasting pain relief for synovitis [391].

Cationic liposomes Lnxc‐CL/miR‐140 loaded loxoprofen and miR‐140 through membrane dispersion and electrostatic interaction, effectively treating OA by eliminating joint inflammation and repairing damaged chondrocytes [392]. Teriparatide‐loaded liposomes encapsulated in injectable hydrogels enable controlled release, promoting chondrocyte proliferation and protecting ATDC5 cells from IL‐1β‐induced damage via PI3K/AKT pathway regulation [393]. Antioxidant and targeted liposomes further optimize efficacy. ChsMA@Lipo, a degradable dual‐antioxidant platform loaded with glycyrrhetinic acid (LQ), combined enzyme‐degradable methacryloylated chondroitin sulfate (ChsMA), which cleared ROS via monomer release with sustained LQ release via lipid‐hydrogel dual barriers [394]. ChsMA@Lipo not only inhibited the polarization of M1 macrophages and the activation of inflammatosomes, but also weakened IL‐1β‐induced ECM degradation in chondrocytes, slowing the progression of OA [394]. FA‐modified astaxanthin (AST)‐loaded liposomes (AST@Lip‐FA) targeted macrophage folate receptors, enabling rapid uptake and preferential accumulation in inflamed joints, thereby exerting anti‐inflammatory, antioxidant, and chondroprotective effects [395].

Mitochondrial dysfunction is a key OA pathogenic factor, driving chondrocyte energy metabolism disorders and apoptosis. Selective removal of dysfunctional mitochondria at the subcellular level can provide energy for chondrocytes, thereby preventing cartilage degeneration and treating OA. Chen et al. developed a chondrocyte‐targeted system (HM@WY‐Lip/UA) by integrating urolithin A (UA, mitophagy activator)‐loaded, chondroaffinity peptide WYRGRL (WY)‐modified liposomes into HA methacrylate hydrogel microspheres (HM) via microfluidic technology [396]. This system efficiently targeted chondrocytes and selectively removed dysfunctional mitochondria, and IAI of HM@WY‐Lip/UA can improve the degradation of cartilage matrix and subchondral osteosclerosis, and has a good protective effect against cartilage degeneration [396]. This mitochondrial‐oriented strategy of has great potential in the subcellular therapy of OA. Therefore, researchers are exploring the direct delivery of normal mitochondria into chondrocytes to treat OA. This treatment may reduce inflammation‐mediated cell death and enhance autophagy. Kim et al. designed fused mitochondrial capsules (FMCs) composed of neutral lipids (PE), cationic lipids (DOTAP), aromatic lipids (Liss Rhod PE), and liposomes, which delivered functional mitochondria to chondrocytes via membrane fusion. FMCs reduced inflammatory cytokines and MMP13 expression, and enhanced the expression of ECM components, thereby promoting cartilage regeneration [397]. These findings suggest that FMCs may be an efficient strategy for delivering mitochondria.

Liposomes, via diverse drug loading and structural modifications, achieve multimodal OA therapy such as anti‐inflammation, cartilage repair, and mitochondrial function regulation, demonstrating significant preclinical potential; however, clinical translation requires further optimization of biocompatibility and delivery efficiency.

##### 4.1.3.3 Extracellular Vesicles

Extracellular vesicles are bioactive nanostructures with diameters ranging from 30 to 150 nm, mediating intercellular communication via transfer of lipids, proteins, and nucleic acids [398]. Their natural biocompatibility, targeting ability, and cargo‐carrying capacity make them ideal OA therapeutic carriers. For instance, small extracellular vesicles derived from M2 macrophages protected articular cartilage and improved gait abnormalities in OA mice by delivering miR‐26b‐5p to target TLR3 and COL10A1 [399]. Platelet‐derived extracellular vesicles (Plt‐exos) significantly promoted the proliferation and migration of chondrocytes in a dose‐dependent manner, enhancing cartilage regeneration and slowing OA progression [400]. The extracellular vesicle inherently express lipids such as phosphatidic acid and cell adhesion molecules, enabling targeted delivery via specific receptor–ligand interactions [401, 402].

MSCs therapy has shown great potential in the research of OA. MSC‐derived extracellular vesicles (MSC‐Exos) are the most extensively studied, with therapeutic efficacy attributed to paracrine mechanisms [403, 404, 405]. As an illustration, MSC‐Exos enhanced sulfated glycosaminoglycans (s ‐ GAG) synthesis inhibited by IL‐1β and suppress the production of NO and MMP13 via AKT/ERK/adenosine monophosphate‐activated protein kinase (AMPK) pathway activation [406]. MSC‐Exos also reduced inflammation and inhibited ferroptosis through the GOT1/CCR2/Nrf2/HO‐1 signaling pathway, thereby improving OA in mouse models [407]. Small extracellular vesicles derived from synovial MSCs activated yes‐associated protein (YAP) by carrying Wnt5a and Wnt5b, and enhanced the proliferation and migration of chondrocytes [408]. Small extracellular vesicles derived from IPFP MSCs (MSCIPFP‐Exos) significantly enhanced chondrocyte autophagy via partially inhibiting mTOR and protect articular cartilage from damage by maintaining cartilage homeostasis [409]. Extracellular vesicles derived from BM‐MSCs significantly reduced the inhibitory effect of IL‐1β on chondrocyte proliferation and migration, promoted ECM synthesis and cartilage repair, and alleviated knee pain in OA rats [410, 411], while lncRNA MEG‐3 may be the key molecule for improving OA [412]. Engineering extracellular vesicles derived from subcutaneous fat (SC) stromal cells (MSCs^SC^‐Exos) deliver miR‐199a‐3p into chondrocytes, exerting a protective effect on articular cartilage by regulating the mTOR–autophagy pathway [413]. Extracellular vesicles derived from umbilical cord MSCs can also upregulate the expression of Type II collagen, significantly downregulate the expression of MMP‐13 in chondrocytes stimulated by IL‐1β, and improve KOA [414]. The small extracellular vesicles secreted by human urine‐derived stem cells exerted similar effects via miR‐140‐5p‐mediated inhibition of osteoclast activity and enhancement of cartilage integrity [415]. These findings confirm that extracellular vesicles derived from MSCs can alleviate joint pathological damage by reducing cartilage destruction. Therefore, optimizing the yield and therapeutic effect of extracellular vesicles derived from MSCs is crucial for promoting their clinical transformation.

Pretreatment of MSCs enhances therapeutic potency by promoting the secretion of extracellular vesicles and enriching their functional cargo. Curcumin pretreatment increased miR‐143/miR‐124 levels in MSC‐Exos, downregulating chondrocyte NF‐κB and ROCK1 to slow OA progression [416]. Fucoglycan pretreated MSC‐Exos (F‐MSCs‐Exo) effectively inhibited the activation of TRAF6 through miR‐146b‐5p, thereby suppressing the inflammatory response and ECM degradation [417]. TNF‐α pretreatment significantly enhanced the PI3K/AKT signaling pathway in IPFP‐MSCs and increased the expression level of autophagy‐related protein 16‐1 (ATG16L1), promoting the secretion of extracellular vesicles (IPFP–MSC–EXOsTNF‐α) and increasing low‐density lipoprotein receptor‐associated protein 1 (LRP1) level in IPFP–MSC–EXOsTNF‐α. IAI of IPFP–MSC–EXOsTNF‐α showed better effects in improving the joint pathological changes and gait of OA mice [418]. Quercetin pretreatment also enhanced the cartilage protection and anti‐inflammatory effects of MSCs‐Exo [419].

Natural extracellular vesicles are limited in clinical applications due to insufficient targeting ability, short circulating half‐life, and uncertainty in the composition and content of functional contents. By using bioengineering techniques to modify natural extracellular vesicles and loading substances such as targeted peptides, exogenous molecules, drugs, proteins, lipids, and nucleic acids into the lumen or surface of exosomes, the targeting and stability of extracellular vesicles can be improved [420, 421]. Hydrogel has good biocompatibility and adjustable physicochemical properties, and can be used as an ideal carrier for extracellular vesicles delivery. Through hydrogels, the release of e extracellular vesicles can be controlled more precisely. Meanwhile, some hydrogels themselves have the function of regulating cell adhesion, proliferation, and differentiation. Therefore, the synergistic effect of hydrogels and extracellular vesicles can further enhance the therapeutic effect [422]. Thermosensitive hydrogels encapsulating PRP‐derived extracellular vesicles (Exo‐Gel) achieved sustained release for 28 days, promoting mouse BM‐MSCs (mBM‐MSCs) and chondrocyte proliferation, enhancing chondrogenic differentiation, and improving talus OA [423]. Thio‐HA microgels loaded with chondrocyte‐targeted extracellular vesicles derived from umbilical cord MSCs (UCMSC‐EXOs) improved therapeutic efficiency and retention time of UCMSC‐EXOs in vivo [424]. Wan et al. developed a novel photocross‐linked spherical collagenous methacrylate hydrogel (GelMA) embedded cartilage‐affinity WYRGRL peptide modified engineered extracellular vesicles loaded with small molecule inhibitor LRRK2‐IN‐1 (W‐Exo@GelMA) [425]. W‐Exo‐L@GelMA exhibited chondrocyte targeting, prolonged joint retention, and enhanced cartilage repair [425]. Yang et al. constructed magnetic polysaccharide hydrogel particles using modified natural polysaccharides, HA, and chondroitin sulfate (CSMA) as microcarriers to capture extracellular vesicles derived from stem cells, and encapsulated them together with the anti‐inflammatory drug diclofenac sodium (DS) in the microcarriers [426]. The DS and extracellular vesicles released in the microcarriers had a synergistic effect in promoting cartilage repair and alleviating the symptoms of OA [426]. Mitochondrial unfolded protein response, as an important component of the mitochondrial quality control system, is crucial for maintaining the homeostasis of chondrocytes. Activating transcription factor 5 (ATF5) plays a key role in regulating the mitochondrial unfolded protein response and promoting metabolic balance in articular cartilage ECM, and may be a promising therapeutic target for OA, but its local metabolism limits its application. Continuous release of ExmodAtf5 was achieved by encapsulating ATF5‐modified RNA (modAtf5) and engineered mBM‐MSCs‐derived extracellular vesicles (ExmodAtf5) in thermosensitive hydrogel for injection (Gel@ExmodAtf5) [427]. Gel@ExmodAtf5 enhanced chondrocyte autophagy via mTOR/Ulk1 pathway regulation, improving therapeutic efficacy and prolonging ExmodAtf5 release [427]. SOD3 has a protective effect on cartilage, and the synovium releases SOD3 through exocrine secretion to affect cartilage function. Hydrogel microspheres loaded with extracellular vesicles enriched with SOD3 (S‐EXOs) can effectively deliver SOD3 to cartilage, enhance the antioxidant capacity of chondrocytes, and maintain the stability of ECM metabolism [428].

Noncovalent modification techniques, such as multivalent electrostatic interactions, ligand–receptor interactions, hydrophobic interactions/membrane engineering, and surface modifications based on nucleic acid aptamers, enhance the targeting and activity of extracellular vesicles. Chondrocyte affinity peptide (CAP)–lysosome‐associated membrane glycoprotein 2b (Lamp2b, extracellular vesicles surface protein) fusion extracellular vesicles (hybrid Cap‐Exo) encapsulate CRISPR/Cas9 plasmids, delivering sgMMP‐13 to deep cartilage matrix and reducing ECM degradation by downregulating the expression of MMP‐13, thereby alleviating OA [429]. Chen et al. constructed CAP/FGF18–hyEXO by a similar method to effectively activate fibroblast growth factor 18 (FGF18) in OA chondrocytes, and CAP/FGF18–hyEXO was encapsulated in methacrylate anhydride modified HA (HAMA) hydrogel microspheres through microfluidic technology and photo‐polymerization to form an injectable microgel system (CAP/FGF18–hyEXO@HMs), which has a self‐renewing hydration layer and provides long‐lasting lubrication [430]. Liang et al. obtained CAP‐fused extracellular vesicles that could efficiently encapsulate miR‐140 and specifically target chondrocytes. CAP‐extracellular vesicles delivered miR‐140 to the deep cartilage region through the dense cartilage‐bone marrow, inhibited cartilage degradation and OA progression in rats [431].

Gene therapy has the potential to promote cartilage regeneration. However, dense, vascularless cartilage rich in aggrecan–GAGs carries a negative charge, hindering the effective transport of nucleic acids. Zhang et al. used the pH value of the buffer solution as a charge inversion switch to anchor the arginine‐rich cationic motif targeting cartilage into the lipid bilayer of extracellular vesicles, fabricating cationic extracellular vesicles with charge inversion, and effectively delivering the encapsulated eGFP mRNA to chondrocytes in the deep tissue [432].

Extracellular vesicles, as drug delivery carriers, can achieve efficient drug delivery, demonstrating their potential as nanoplatforms for biotherapy and drug delivery. While preclinical studies demonstrate robust anti‐inflammatory, chondroprotective, and regenerative effects, clinical translation is hindered by challenges such as scalability, standardized production, and long‐term safety [433]. Future research should focus on optimizing engineering strategies and conducting large‐scale clinical trials to validate their clinical utility.

### 4.2 Physical Therapy

Physical therapy for OA aims to alleviate pain, reduce joint swelling, inhibit chronic exudation in perilesional soft tissues, improve joint mobility and function, and delay disease progression. Common physical therapy modalities are summarized in Table 3.

**TABLE 3:** Nonpharmacological physical therapies for osteoarthritis.

Therapy	Main indications	Biological mechanisms	References
Acupuncture and electroacupuncture therapy	Knee osteoarthritis, hip osteoarthritis, hand osteoarthritis	As a conventional complementary therapy, acupuncture alleviates symptoms by stimulating specific acupoints (such as Zusanli, Yanglingquan, Qianjinquan), which improves local microcirculation, relieves pain, and modulates systemic immune function. Electroacupuncture, combining traditional acupuncture with microcurrent stimulation, further enhances local blood flow, reduces pain and joint stiffness.	[434, 435, 436, 437]
Electrical stimulation	Knee osteoarthritis, erosive hand osteoarthritis	Electrical stimulation exerts therapeutic effects by regulating neuromuscular function, improving regional blood circulation, and alleviating pain via stimulating nerve endings to promote endorphin release.	[438, 439, 440, 441, 442]
Infrared therapy	Temporomandibular, joint osteoarthritis, knee osteoarthritis	Infrared therapy (wavelength 760 nm–400 µm) is a noninvasive intervention that achieves anti‐inflammatory and analgesic effects via thermal, electromagnetic, and photochemical mechanisms.	[443, 444, 445]
High‐energy laser	Hand osteoarthritis, knee osteoarthritis	High‐energy laser (wavelength 650–1064 nm) integrates photothermal, photomechanical, and photocatalytic effects to enhances ATP metabolism and blood circulation, and suppresses inflammatory responses.	[446, 447, 448, 449]
Extracorporeal shock wave therapy	Knee osteoarthritis, thumb osteoarthritis	Extracorporeal shock wave therapy (ESWT) delivers pulsed mechanical waves to target areas, relieving tissue adhesions, fragmenting osteophytes, and exerting analgesic effects via substance P release. It also modulates cell membrane permeability, accelerates ion exchange, promotes metabolic waste clearance, and enhances vascular growth factor expression to support angiogenesis and tissue repair.	[450, 451, 452, 453, 454]
Ultrasound therapy	Knee osteoarthritis, hip osteoarthritis	Ultrasound therapy utilizes high‐frequency sound waves (0.8‐3 MHz) to to improve local blood circulation, inhibit nociceptive signal transmission to relieve pain, and promote the repair of damaged soft tissues and articular cartilage.	[455, 456, 457, 458, 459, 460]
Ultrashort wave therapy	Knee osteoarthritis	Ultrashort wave therapy (frequency 30–300 MHz) alleviates osteoarthritis symptoms through high‐frequency electromagnetic waves, reducing local inflammation, improving blood circulation, and accelerating tissue repair. Repeated sessions promote the repair of articular cartilage lesions, significantly decrease synovial sac thickness, and relieve knee pain in knee osteoarthritis patients.	[461]

As a supplementary and auxiliary form of physical therapy, exercise suppresses inflammation, prevent and improve cartilage degeneration. Accumulating evidence indicates that exercise effectively improves pain, joint stiffness, dysfunction, and muscle weakness in OA patients [462, 463]. Currently, OA‐specific exercise training encompasses aerobic exercise, strength training, neuromuscular exercise, balance/proprioception training, and traditional Chinese exercises. Meta‐analyses have shown that exercise exerts comparable effects on pain and function to oral NSAIDs and acetaminophen [464]. Given its superior safety under medical supervision, exercise should receive greater clinical attention, particularly in elderly patients at high risk of adverse drug events.

#### 4.2.1 Aerobic Exercise

Aerobic exercises, including walking, jogging, cycling, aerobics, increase cardiopulmonary function, reduce oxidative stress, promote adipose metabolism, and enhance systemic immunity [465]. Aerobic exercise alleviates pain, improves physical function, and enhances QoL in OA patients [466].

Clinical evidence supports its therapeutic value across populations. Patients with KOA have weakened immune function. In elderly women with KOA, 12 weeks of walking (3 sessions/week) improved QoL and physical performance, potentially via exercise‐induced leukocyte production and T‐cell activation to enhance immunity [467]. Consistently, six weeks of aerobic exercise relieved pain and functional impairment in postmenopausal women with KOA [468]. Among patients with KOA aged more than 50 years, walking reduced the frequency of knee pain, with a significantly lower risk of new‐onset frequent pain compared with nonexercisers [469]. Six weeks of retro walking significantly alleviated pain and dysfunction in patients with KOA and improved the strength and athletic performance of the quadriceps femoris [470]. Aquatic exercise also provided clinical benefits to patients with KOA, significantly alleviating pain [471]. Static cycling has been reported to relieve pain and improve motor function in patients with KOA, but had no significant effect on reducing stiffness [472]. For women with mild KOA, 12‐month of progressive high‐impact jumping increased the strength of the femoral neck without affecting the knee cartilage [473, 474], offering a potential strategy for preventing hip fractures in postmenopausal women.

Notably, the therapeutic effect of aerobic exercise is not directly proportional to the intensity. An 8‐week unsupervised home cycling program for middle‐aged and elderly KOA patients found that both moderate‐intensity continuous training and high‐intensity interval training (HIIT) significantly improved WOMAC scores, with HIIT yielding greater physical function benefits [475, 476]. However, in KOA patients with Type 2 diabetes, high‐intensity training significantly improved blood glucose control, but it was not superior to moderate‐intensity training in alleviating pain and improving function [477]. The conclusion that high‐intensity aerobic exercise does not benefit patients with KOA more has also been confirmed by other studies [478].

Aerobic exercise is suitable for most OA patients, with intensity and form adjusted according to disease severity, age, comorbidities, and exercise tolerance. Low‐intensity aerobic exercise may be more friendly to patients with severe KOA, while high‐intensity aerobic exercise is more suitable for those with mild KOA. HIIT may be preferred for OA patients with chronic metabolic diseases.

#### 4.2.2 Strength Training

Strength training is indispensable for restoring muscle strength in OA patients, alleviating pain and stiffness, improving physical function, enhancing lower limb muscle shock absorption during walking, and reducing joint impact [479, 480]. A 1‐year multigroup randomized controlled trial showed that 12 weeks of strength training increased the quadriceps femoris mass and maximal oxygen uptake in patients with KOA, with no significant differences compared with aerobic exercise (stationary cycling) [481]. Strength training was also as effective as Yoga in relieving knee pain [482], confirming its role as a core adjuvant therapy as aerobic exercise for OA [483]. Based on muscle contraction patterns, strength training includes concentric, isometric, and eccentric exercises, all of which enhance knee extensor/flexor strength and relieve pain of patients with KOA [484]. A randomized controlled study evaluated the effects of different strength training modalities on quadriceps structure and function in KOA and found that isometric exercise increased the length and thickness of the muscle bundles on the strengthened side and the length of the muscle bundles on the contralateral side, thereby increasing the strength of the bilateral knee extensor muscles; isokinetic concentric exercise increased the thickness of the bilateral muscles and the length of the muscle bundles on the contralateral side; and isotonic exercises increased the thickness of muscles on both sides [484], suggesting modality‐specific mechanisms for strength enhancement.

Similar to aerobic exercise, strength training of different intensities also has different effects on improving joints. Low‐load isotropic resistance training effectively enhanced the muscle strength of patients with KOA [485]. An 18‐month follow‐up showed that high‐intensity strength training was not superior to low‐intensity strength training in reducing knee pain or knee compression force [486]. On the contrary, 12 weeks of progressive strength training improved maximum knee flexor strength and pain relief more effectively than low‐intensity exercise in patients after ACL reconstruction [487].

Moderate‐intensity resistance training (MIRT) and high‐intensity resistance training (HIRT) improve muscle strength and muscle mass in OA patients without exacerbating the disease process. For OA patients unable to tolerate high intensity, blood flow restriction (BFR) combined with low‐intensity strength training may be a good choice. BFR can provide the metabolic stimulation needed to achieve muscle strength and functional benefits while reducing mechanical load. Low‐intensity resistance training combined with BFR (LIRTBFR) achieved similar effects to MIRT and HIRT in enhancing muscle strength, and is significantly superior to low‐intensity training alone [488]. A recent randomized controlled study has demonstrated that incorporating BFR into traditional exercise programs yielded sustained improvements in pain, symptoms, QoL, and function, lasting at least 3 months postintervention [489]. Preoperative low‐load BFR resistance training (BFR‐RT) improved physical function in patients undergoing TKA [490]. Eight weeks of preoperative BFR‐RT (3 sessions/week) significantly enhanced 1‐repetition maximum (1RM) leg lift strength, 1RM knee extensor strength, and maximal voluntary isometric contraction knee extensor strength at 3‐month follow‐up, though it did not improve preoperative knee ROM or KOOS subscale scores compared with conventional care [491]. BFR prerehabilitation significantly improved muscle function and QoL before TKA surgery.

Strength training is an important component of exercise training for patients with OA, especially those with muscle weakness, decreased lower limb muscle strength, and poor joint stability. From the perspectives of society and healthcare, strength training may be more cost effective than aerobic training for adjuvant KOA treatment [492]. Patients unable to tolerate high‐intensity training, such as elderly patients with severe KOA or those with poor physical condition, are suitable for low‐load isotropic resistance training or low‐intensity strength training combined with BFR. Patients after ACL reconstruction benefit more from progressive high‐intensity strength training. In addition, patients undergoing TKA can benefit from preoperative low‐load BFR resistance training to improve preoperative physical function and muscle strength. For KOA patients with limited exercise tolerance but needing to enhance muscle strength, BFR‐assisted low‐intensity training is an optimal choice. Concentric/isometric/eccentric modalities and BFR‐assisted low‐intensity training offering tailored options for patients with varying disease severity and exercise tolerance, and combining different strength training modalities for OA patients at various stages represents a promising future direction.

#### 4.2.3 Neuromuscular Exercise

Neuromuscular exercise enhances nerve–muscle coordination, strength, joint stability, coordination, and athletic performance, while reducing injury risk [493]. The core goal of neuromuscular exercise is to improve proprioceptive control and achieve joint stability, serving as an effective alternative or supplement to aerobic/strength training for hip and KOA [494]. A randomized, single‐blind, controlled trial found that neuromuscular exercise significantly improved cartilage matrix quality and reduced knee joint load in patients with mild KOA, with efficacy comparable to analgesic/anti‐inflammatory drugs [495]. Compared with pharmacotherapy, it may be superior for long‐term relief of swelling/stiffness and avoiding drug‐related side effects [496]. Combining celecoxib with neuromuscular exercise yielded greater pain and physical function improvements in KOA patients [497]. Multicenter randomized controlled trials found that neuromuscular exercise was comparable to progressive resistance training for hip OA [498, 499]. Hamstring tightness exerts more pressure on the knee joint, reduces its ROM, and leads to compensatory movements, thereby aggravating KOA. For KOA patients with hamstring tightness, both instrument‐assisted soft tissue mobilization and proprioceptive neuromuscular stretching improved hamstring flexibility and relieved pain [500].

Personalized and progressive neuromuscular exercises improved patient‐reported outcomes (PROs) and physical functions, including independent mobility and knee extensor strength, joint ROM, especially in elderly patients with severe primary KOA [501, 502]. Preoperative neuromuscular exercise (8 weeks of supervised training) before hip and knee replacement surgery could effectively improve the postoperative QoL of patients [503], enhance the activation ability and strength of the knee extensor muscles [504]. Combining focal vibration training, neuromuscular exercises also significantly improved balance, knee joint position sense and knee joint function, facilitating faster and more effective post‐TKA recovery and reducing complications/injury risk [505]. Clinically, neuromuscular exercise has a good therapeutic effect on patients with OA. For end‐stage KOA patients who need to undergo TKA, preoperative neuromuscular exercise effectively relieved postoperative pain and increase muscle strength. At present, neuromuscular training mainly focuses on patients after joint replacement surgery, though additional research is needed to validate its efficacy in early‐stage disease.

Neuromuscular exercise is suitable for OA patients with poor nerve–muscle coordination, decreased joint stability, and poor proprioception, including patients with mild to severe KOA. It is particularly suitable for elderly patients with severe primary KOA, patients with hip or KOA who need adjuvant treatment, KOA patients with hamstring tightness, and patients undergoing hip or knee replacement surgery. For end‐stage KOA patients requiring TKA, preoperative neuromuscular exercise can effectively improve postoperative recovery. It is also suitable for KOA patients who need long‐term relief of swelling and stiffness and wish to avoid drug‐related side effects.

#### 4.2.4 Traditional Chinese Exercises

Traditional Chinese exercise therapies, including Baduanjin, Tai Chi, and Wu Qin Xi, feature slow, gentle, and symmetrical movements that enhance lower limb muscle strength and stabilize joint structures.

Baduanjin is a traditional Chinese medical therapy with a long history of medical application in China and is an important part of complementary and alternative medicine. Baduanjin combines deep breathing with slow and gentle movements. It not only regulates breathing and relax the body and mind, but also relieve morning stiffness and reduce the impact of pain on the body, especially alleviating musculoskeletal pain in the elderly [506, 507]. Meta‐analysis of randomized controlled trials showed that Baduanjin significantly improved the total WOMAC score, pain score, stiffness score, and physical function score of patients with KOA, with no adverse events [508]. Combining Baduanjin with strengthening exercises was more effective than single‐modality exercise in relieving pain, improving function, enhancing self‐efficacy, and boosting QoL in community‐dwelling elderly KOA patients [509]. Mechanistic studies using multimodal MRI suggested that Baduanjin alleviates knee pain by reducing resting‐state functional connectivity (rsFC) between the right periaqueductal gray matter (PAG) and medial orbitofrontal cortex, and decreasing rsFC between the left ventral tegmental area (VTA) and medial orbitofrontal cortex, potentially via regulation of the descending opioidergic pathway and reward/motivation system [510]. In addition, patients with KOA exhibited higher rsFC between the left dorsolateral prefrontal cortex (DLPFC) and supplementary motor area (SMA); Baduanjin reduced this rsFC while increasing DLPFC‐anterior cingulate gyrus rsFC, with altered DLPFC–SMA connectivity correlating with serum biomarker levels [511]. These results indicate that Baduanjin exercise is an effective and safe adjunctive therapy that can alleviate pain and function in patients with KOA.

Originating from Chinese martial arts, Tai Chi is a gentle aerobic exercise that relaxes the body and mind. As a nonpharmaceutical intervention, it has gained attention in OA comprehensive management [512, 513], with efficacy comparable to other exercises for pain, mobility, self‐reported function, and stiffness [514]. Tai Chi is recommended by EULAR for the treatment of KOA [84]. Two to five weeks Tai Chi relieved the pain of KOA patients, and the effect is dependent on the duration of exercise [515]. Tai Chi can change the gait and plantar pressure load pattern of KOA patients during walking [516], improve the walking function and posture control of elderly people with KOA and reduce the mechanical stress on the joints [517]. Twenty‐four weeks of Tai Chi improved ankle/knee proprioception and balance on hard/foamed surfaces [518, 519]. After 14 weeks of Tai Chi, the ROM and maximum knee extension torque of the knee joint in elderly patients were reduced, accompanied by a significant increase in the activation of the anterior tibial muscle, indicating that Tai Chi can bring significant clinical and biomechanical benefits to elderly patients with KOA [520]. Tai Chi also improved physical functions of KOA patients, including pain, stiffness, and dynamic balance [521, 522], potentially via enhancing fatty acid catabolism, protein metabolism, and lipid redistribution in postmenopausal women, and strengthening functional/structural connectivity between the medial prefrontal cortex and amygdala [523, 524]. Meta‐analysis compared the effects of different traditional exercises on KOA patients and found that Tai Chi most effective for improving VAS scores, WOMAC pain scores, and WOMAC function scores, while Baduanjin ranked first for WOMAC stiffness scores [525]. Another study confirmed that traditional exercises, including Tai Chi, Baduanjin, Yijinjing and Wuqinxi, outperformed conventional care, with Baduanjin best for stiffness, function, and overall scores, and both Baduanjin and Tai Chi optimal for pain relief [526]. High‐quality RCTs are needed to validate these findings and optimize intervention parameters.

Traditional Chinese exercises are suitable for OA patients with poor exercise tolerance, elderly OA patients, patients with mild to moderate KOA, and community‐dwelling OA patients who need safe and gentle adjuvant therapy. Baduanjin is particularly suitable for elderly OA patients with musculoskeletal pain and morning stiffness, as well as community‐dwelling elderly KOA patients who need to improve self‐efficacy and QoL. Tai Chi is suitable for KOA patients recommended by EULAR for nonpharmaceutical intervention, elderly KOA patients with poor balance and gait, and postmenopausal women with KOA. Both Baduanjin and Tai Chi are optimal for OA patients needing pain relief, while Baduanjin is more suitable for those with obvious stiffness.

#### 4.2.5 Digital Exercise Therapy

Despite its benefits, supervised exercise training is often inaccessible or inconvenient for many OA patients. With the rapid development of internet technology, internet‐based platforms have increasingly delivered health and exercise services, making home‐based remote rehabilitation a widely used OA rehabilitation strategy. Therapists use telecommunication technology to guide patients remotely, enabling comfortable and convenient home exercise and addressing exercise barriers in the OA population. A randomized trial comparing video‐based telemedicine exercise programs found that telemedicine‐delivered exercise improved pain and function in overweight/obese KOA patients [527]. A 12‐week home exercise program using a mobile app and two accelerometers placed above/below the affected knee for movement monitoring showed that sensor‐assisted app‐based training was more effective than control in relieving pain and improving physical function [528]. Meta‐analysis confirmed that digital exercise therapy alleviates OA pain and improves function, serving as a valuable adjuvant to OA rehabilitation [529]. It offers significant convenience for patients requiring long‐term treatment, enabling home‐based rehabilitation. Multiple RCTs on online exercise interventions for hip/KOA are ongoing [530].

Digital exercise therapy is suitable for OA patients who cannot access supervised exercise training, need home‐based remote rehabilitation, or have inconvenience in going out. It is particularly suitable for overweight/obese KOA patients, OA patients requiring long‐term rehabilitation treatment, and patients who need convenient and comfortable exercise intervention. It is also applicable to hip OA or KOA patients who are willing to receive internet‐based exercise guidance and have basic operation ability of digital devices.

Although a large amount of evidence suggests that exercise may improve pain, physical function, and QoL in patients with OA in the short term, the clinical significance of this effect is questionable, especially in the medium and long term [531, 532]. Most trials lack blinding, potentially introducing reporting bias [466]. Future research evaluating exercise therapy is encouraged to include physical activity outcomes and longer follow‐ups to increase the certainty of the evidence. Since individuals with higher levels of pain severity and poorer physical function at baseline benefit more than those with lower levels of pain severity and better physical function at baseline, therapeutic exercises for individuals with higher levels of OA‐related pain and disability at baseline may be valuable [533].

Physical therapy offers a safe, effective, and low‐cost nonpharmaceutical approach for OA management. Tables 4 synthesize and present the most recent clinical trials focused on physical interventions for OA conducted in recent years. Individualized exercise programs tailored to disease severity, comorbidities, and exercise tolerance and integration of digital tools to improve accessibility represent key future directions, while longer‐term RCTs are needed to confirm sustained efficacy.

**TABLE 4:** Exercise, lifestyle, and rehabilitation interventions for osteoarthritis.

Condition	Intervention	ClinicalTrials ID	Enrollment	Key findings	References
Osteoarthritis	Osteoarthritis physical activity care pathway (OA‐PCP)	NCT03780400	60	3‐month OA‐PCP significantly reduces WOMAC pain scores and WOMAC function scores. It has good feasibility and acceptability.	[534, 535]
Osteoarthritis	OA‐PCP, attention control	NCT04533711, 20–2134, 4R33AG056568‐03	240	Compared with attention control, OA‐PCP increases daily patient activity steps. No significant difference is observed in WOMAC function subscale score improvement.	
Osteoarthritis	Pain coping skills training (CST)	NCT02560922, 15–1189, AD‐1408‐19519	248	African American participants rated CST's overall help in managing OA symptoms as 8.0 ± 2.2 (out of 10). CST is acceptable among African Americans with OA.	[536, 537, 538]
Knee osteoarthritis	Mindset intervention, no‐intervention group	NCT05698368, 69227	458	Mindset intervention significantly improves mental state and physical activity levels, but pain reduction is not significant. The mindset group shows improvements in perceived surgical need, self‐imposed physical limitations, and exercise fear.	[539]
Knee osteoarthritis, hip osteoarthritis	Exercise	NCT02609672	24	Compared with the no‐exercise group, the exercise group shows significant improvements in work ability, pain, function, and depressive symptoms.	[540]
Osteoarthritis	Diet and exercise, attention control	NCT04946344, IRB00033618, U01AR068658	16	Compared with attention control, diet & exercise significantly improves WOMAC function scores, SF‐36 quality‐of‐life scores, and 6‐min walking distance.	
Osteoarthritis	Diet and exercise, attention control	NCT02577549, IRB00033618, U01AR068658	823	Patients with Grade III obesity and KOA have significantly higher pain levels, poorer health‐related quality of life (HRQL), and worse gait characteristics than overweight/obesity patients. 18‐month diet and exercise interventions significantly improve knee pain.	[4, 541, 542]
Knee osteoarthritis	High intensity interval training (HIIT)	NCT03281668	29	Participants completed 12 weeks of HIIT, showing good compliance and tolerability. HIIT enhances physical function, reduces knee pain scores, and improves static standing balance, knee extensor/flexor strength, and cardiopulmonary health.	
Knee osteoarthritis	Stepped exercise program, arthritis education	NCT02653768, IIR 14–091	345	Stepped exercise program is a step‐by‐step exercise plan for patients with osteoarthritis (STEP‐KOA). 65% of participants progressed to Step 2, and 35% to Step 3. Compared with the control group, the STEP‐KOA group shows moderate improvements in KOA symptoms and enhanced quality of life.	[543, 544, 545]
Knee osteoarthritis	Exercise	NCT03628508, HSEARS20170406003	40	A 6‐week exercise program changes external knee adduction moment and KOOS pain scores.	
Knee osteoarthritis	Delaware physical exercise and activity (PEAK)	NCT04980300, 1730922	103	The 12‐week Delaware PEAK increases daily patient activity steps, reduces VAS pain scores, and improves KOOS Scores.	[546, 547]
Knee osteoarthritis	Exercise‐based physical therapy	NCT04243096, 5540E	62	Participants underwent 12 weeks of supervised exercise, with wearable sensors used for motion capture and evaluations of strength, balance, gait, and joint movement. Wearable sensors show high test‐retest reliability, enabling remote assessment of motor ability.	[548, 549]
Knee osteoarthritis	MONITOR‐OA (Group Education + Fitbit Flex + PT remote coaching), same intervention with a 2‐month delay	NCT02315664, H14‐01762	61	MONITOR‐OA includes group education, Fitbit Flex (wireless activity tracker), and biweekly online/telephone PT coaching. Immediate MONITOR‐OA improves moderate‐to‐vigorous physical activity, daily steps, daily activities, and quality of life in newly diagnosed KOA patients. A 2‐month delayed intervention shows no such improvements.	[550, 551]
Osteoarthritis	Fit & strong!	NCT04536727, 2013‐1027	28	Fit & strong! increases Physical Activity Scale for the Elderly (PASE) scores.	
Osteoarthritis	Physical activity, behavior intervention, attention control	NCT03226106, F2417‐R	115	Physical activity behavior intervention increases free‐living daily step count but shows no significant improvement in physical function.	
Osteoarthritis,	Positive Activities (PA) Program, Attention Control (AC) Program	NCT02223858, IIR 13–080	360	Both the PA Program and AC Program significantly reduce WOMAC pain and dysfunction scores in veterans with KOA, with no significant difference between groups.	[552]
Knee osteoarthritis	KneeBright Group, standard rehabilitation group	NCT04187092, IRB‐HSR# 21926	38	The KneeBRIGHT system combines electromyography (EMG) biofeedback with video game‐based muscle strengthening. Compared with the standard rehabilitation group, the KneeBright Group shows significant improvements in KOOS (symptoms, pain, quality of life).	
Osteoarthritis	Engage‐PA, treatment‐as‐usual with fitness tracker (TAU+)	NCT04490395	40	Engage‐PA is an intervention that combines personal values with activity rhythms. Compared with TAU+, Engage‐PA better improves arthritis‐related pain and function in the elderly.	[553]
Osteoarthritis	Foot rotation modification, waiting period‐delayed group	NCT04683913, H20‐03086	20	The 6‐week TELEMOD (video/telephone‐based gait modification) has a 75% satisfaction rate with no significant adverse events. Compared with the control group, TELEMOD significantly changes patient foot progression angle; compared with baseline, follow‐up shows significant improvements in pain and knee torque.	[554]
Osteoarthritis	Functional strength integration (FSI)	NCT02920866, O2251‐I 16–0956	95	FSI significantly increases 6‐min walking distance, 30‐s sit‐to‐stand count, hip abductor muscle endurance, and hip adductor torque in patients undergoing total hip arthroplasty for hip OA.	[555]
Knee osteoarthritis	Hybrid training using electrodes and joint motion sensors, isokinetic training with isokinetic dynamometer	NCT02802878, STUDY00003872	42	Hybrid training using electrodes and joint motion sensors relieves pain and improves physical performance, but its effects on muscle strength enhancement, pain reduction, and function improvement are similar to isokinetic training.	[556]
Knee osteoarthritis	Low‐carbohydrate diet (LCD)	NCT04343716, IRB‐300005145, 5P30AG031054‐13	19	A 6‐week LCD significantly improves daily pain, physical function decline, and depressive symptoms in non‐Hispanic Black patients with KOA. Induced pain during timed walking and chair‐standing tasks also decreases.	[557]
Hip osteoarthritis, knee osteoarthritis	Brief interactive decision aid, long video decision aid	NCT02625402, 2013P001794	58	Significant differences exist between the brief interactive decision aid and long video decision aid in improving hip and knee decision quality instruments knowledge subscale scores.	
Hip osteoarthritis, knee osteoarthritis	Interactive decision aid, video decision aid	NCT02729831, 2016P000229	1220	Compared with the video decision aid, the interactive decision aid significantly increases patient knowledge scores (hip/knee decision quality tool), improves EQ‐5D quality‐of‐life scores, and reduces the proportion of patients requiring surgery.	[558]
Knee osteoarthritis	Self‐transcranial direct current stimulation (tDCS)	NCT03747640, HSC‐SN‐18‐0885	30	tDCS (2 mA for 20 min, 15 sessions/week for 3 weeks) reduces numeric rating scale pain scores and WOMAC scores.	
Knee osteoarthritis	Transcranial direct current stimulation (tDCS)	NCT04016272, HSC‐SN‐19‐0469, R15NR018050	123	tDCS (2 mA for 20 min, 15 sessions/week for 3 weeks) reduces pain intensity and improves joint range of motion in elderly knee osteoarthritis patients.	[559, 560, 561]
Knee osteoarthritis	Cranial electrical stimulation (CES)	NCT04016259, HSC‐SN‐19‐0452	30	CES (60 min/day, 5 days/week for 2 weeks) reduces pain and pain sensitivity in knee osteoarthritis patients.	
Knee osteoarthritis	Pulsed electromagnetic field (PEMF)	NCT02696083, RBI.2015.004	19	PEMF reduces synovial marker levels, including BMP‐related proteins, cytokine growth factors, bone remodeling‐related proteins, adhesion/matrix proteins, and MPP proteins.	
Knee osteoarthritis	Whole body vibration (WBV), local muscle vibration	NCT02605876 15–0838	75	WBV alleviates abnormal gait biomechanics after anterior cruciate ligament reconstruction (ACLR) and may reduce post‐ACLR osteoarthritis risk.	[562, 563, 564]

## 5 Interventional Treatment for OA

As a complement to conservative treatment, interventional treatment is designed for patients who fail to respond adequately to conservative measures, particularly those in moderate to severe stages of OA. It encompasses a series of therapeutic measures involving minimal to moderate invasion of the joint or surrounding tissues, aiming to achieve targeted symptom relief, structural improvement, or functional restoration. Consistent with the core therapeutic goals of OA, interventional treatment focuses on relieving pain, preserving joint function, and addressing advanced pathological changes. In the early to moderate stage, intra‐articular joint injections serve as minimally invasive restorative interventions; in the moderate stage, this also includes minimally invasive interventional treatments such as radiofrequency and joint cavity lavage, as well as joint debridement and osteotomy; for patients with severe OA, reconstructive treatment such as TKA is the main intervention. Collectively, interventional treatment provides graded therapeutic solutions to address the unmet needs of patients with progressive OA, ultimately improving long‐term joint function and QoL. Table 5 introduces that clinical trials of interventional therapy are currently underway.

**TABLE 5:** Surgical and injectable advanced interventions for osteoarthritis.

Condition	Intervention	ClinicalTrials ID	Enrollment	Key findings	References
Knee osteoarthritis	Amniotic tissue injection (BioDRestore), Kenalog	NCT02767492 Pro00048698	90	BioDRestore and Kenalog relieve pain and improve function. During 1‐year follow‐up, more patients in the BioDRestore group maintain pain relief and function.	[565]
Osteoarthritis	GID SVF‐2	NCT02726945, GIDOA‐01	39	Intra‐articular autologous stromal vascular fraction (SVF) injection significantly reduces knee osteoarthritis symptoms and pain, with effects lasting at least 12 months.	[566]
Knee osteoarthritis	Euflexxa (1% sodium hyaluronate)	NCT03459365, 17–1668	30	Euflexxa increases intra‐articular hyaluronate concentration, improves VAS pain scores, and has no serious adverse events.	
Knee osteoarthritis	Monovisc	NCT04204265, MON 18‐02		Monovisc is a single‐injection cross‐linked sodium hyaluronate. A single injection relieves knee osteoarthritis pain and increases knee flexibility/mobility.	
Knee osteoarthritis	Calypso Knee System (Implantable Shock Absorber, ISA), high tibial osteotomy (HTO)	NCT03671213 (CP0001), NCT03838978 (CP0002).	81	ISA are superior to HTO in improving patient pain and function. ISA surgery is well tolerated with stable efficacy for at least 24 months.	[567]
Knee osteoarthritis	Geniculate Artery Embolization (GAE), Diagnostic Test: Sham Procedure	17‐2701	21	Compared with the sham procedure, GAE reduces mean WOMAC scores and VAS pain scores.	
Knee osteoarthritis	Verasense	NCT03628378, AAAR6137	130	Verasense is a wireless joint load sensor used for real‐time monitoring of soft tissue balance during knee replacement surgery. Two years after total knee arthroplasty (TKA), sensor‐assisted soft tissue balancing does not provide additional benefits in knee range of motion or clinical outcomes.	[568]
Chronic osteoarthritis	PERSONA Total Knee, Vanguard Total Knee, NexGen Total Knee	NCT03969654, CMU2018‐34K	181	Robotic‐assisted TKA (including PERSONA, NexGen, Vanguard prostheses) is superior to conventional TKA in improving Oxford Knee Scores and postoperative pain, with good patient satisfaction.	
Knee osteoarthritis	Stryker Posterior Cruciate Retaining (PCR) TKA, Stryker Posterior Stabilized (PS)TKA, Zimmer PCR TKA, Zimmer PS Total TKA	NCT04321356, WIRB20180745	100	Stryker Triathlon PCR TKA increases anterior‐posterior (AP) translation of the medial femoral condyle during medial deep knee flexion; Zimmer Persona PS TKA increases medial femoral condyle AP translation during deep knee flexion. Stryker Triathlon PS TKA and Zimmer Persona PS TKA increase medial femoral condyle AP displacement during lateral deep knee flexion; Stryker Triathlon PCR/PS TKA increase external femorotibial axial rotation during deep knee flexion.	
Knee osteoarthritis	Journey II Bi‐Cruciate Stabilized (BCS) TKA, Journey II Cruciate Retaining (CR) TKA, Journey II Bi‐Cruciate Retaining (XR) TKA	NCT04612036, WIRB20203268	60	Journey II Bi‐Cruciate Stabilized TKA most effectively increases medial/lateral AP translations and axial rotation; Journey II Cruciate Retaining TKA most effectively increases maximum flexion.	
Knee osteoarthritis	TSolution One	NCT03017261, 16‐PROTO‐01	115	TSolution One is an active robot‐assisted system designed for TKA. It reduces surgical trauma, optimizes implant positioning, is well‐tolerated in knee osteoarthritis patients, and has no serious adverse events.	[569]
Knee osteoarthritis	KneeAlign 2	NCT02695329	100	The KneeAlign 2 system is a navigation system for knee joint surgery. Using KneeAlign 2 during TKA increases the success rate of surgical alignment.	
Knee osteoarthritis	GKS Prime Flex Mobile knee	NCT05810285, VITALECOQ2020	180	Using vitamin E polyethylene in TKA increases American Knee Association (KSS) scores but has no effect on implant survival.	
Knee osteoarthritis	Embozene MicroSpheres	NCT03491397, 18–000560	40	Embozene is a microsphere composed of a hydrogel inner layer and a Polyzene‐F outer layer. Embozene microspheres reduce blood flow to perijoint tissues, limiting local inflammation and alleviating knee osteoarthritis pain.	
Osteoarthritis	NOLTREX	NCT03897686, IA/PAAG‐SI/OA/2019	144	NOLTREX significantly improves WOMAC total scores, pain scores, and stiffness/function scores in Grade II–III osteoarthritis patients, with efficacy superior to placebo. Two courses is inferior to one course of NOLTREX treatment.	
Osteoarthritis	NOLTREX	NCT06429319, IA/PAAG‐SI/OA/2020	65	Compared with one course of NOLTREX treatment, two courses significantly improve WOMAC total scores, pain scores, and stiffness/function scores.	
Osteoarthritis	Autologous conditioned plasma (ACP)	NCT02713542	90	Intra‐articular ACP injections significantly reduce patient pain with no serious adverse events.	

### 5.1 IAI Therapy

For patients with OA of varying severity in one or more joints and who are ineligible for surgery, IAI of biological agents offers valuable symptom relief and functional improvement. Key agents include corticosteroids, HA, PRP, bone marrow aspirate concentrate (BMAC), and ADSCs and systematic reviews and network meta‐analyses have shown that HA, PRP, and their combination exhibit good safety in KOA treatment, with no increased risk of treatment‐related adverse events [570, 571].

#### 5.1.1 Corticosteroids

Corticosteroids possess potent anti‐inflammatory and immunosuppressive properties, exerting therapeutic effects by reducing vascular permeability, inhibiting the synthesis/secretion of inflammatory mediators, protecting articular cartilage, and regulating cartilage catabolism. Triamcinolone acetonide is a commonly used corticosteroid in OA clinical practice [572]. A randomized controlled trial has confirmed that ultrasound‐guided intra‐articular triamcinolone acetonide injection alleviates pain in patients with hip OA [573]. Meta‐analysis evidence further confirms that both ultrasound‐ and fluoroscopy‐guided IACS injections effectively relieve short‐ and long‐term hip OA pain [574, 575]. while IACS also reduces local pain and improves function in HOA [576]. However, IACS efficacy in KOA is inconsistent [575]. Some studies have found that IACS is only effective in alleviating KOA pain for a short period of time [577], while others found the effect of IAI of glucocorticoids is not superior to that of placebo [578]. leading to its common use in combination with other therapies for KOA [579, 580].

It is worth noting that the use of corticosteroids in the treatment of OA has always been controversial [581, 582]. Although corticosteroids provide temporary pain relief, they pose potential risks of joint damage, including accelerated OA progression, subchondral bone nonunion, osteonecrosis, and rapid joint destruction [583]. Additionally, multiple IACS injections do not offer better pain relief than placebo [584, 585], necessitating strict limitations on injection frequency and duration. Corticosteroids are effective for short‐term pain relief in hip OA and HOA but exhibit inconsistent efficacy in KOA; their potential for long‐term joint damage requires cautious use with strict dosage and frequency constraints.

#### 5.1.2 Hyaluronic Acid

HA is a key component of the chondrocyte ECM, contributing to proteoglycan complex formation and cartilage deformation resistance. Pharmacokinetic time‐effect model simulations show therapeutic effect plateaus of HA at ∼2 months, with superior efficacy over placebo in OA. Patients with younger age, higher weight, higher baseline symptom severity, and lower K–L grade seemed to benefit more from HA treatment [586, 587]. Intra‐articular HA injection has been reported to delay the progression of OA by reducing the production of degrading MMPs by decreasing soluble inflammatory mediators such as IL‐1β, IL‐6, and TNF‐α, or by enhancing the synthesis of endogenous proteoglycans and GAGs [588, 589]. HA also alleviates pain and improves joint function by regulating the rheological properties of synovial fluid, including increasing its viscosity, reducing the coefficient of friction and enhancing its lubricity [590].

The treatment of primary glenohumeral OA (GH‐OA) is challenging, while HA has become a promising nonsurgical treatment method for GH‐OA. Intra‐articular HA injection effectively relieved pain and significantly improved symptoms more effectively than corticosteroids [591]. In addition to IAI, oral administration of FlexPro MD (FP‐MD, a complex of krill oil, AST and low molecular weight HA) significantly improved the WOMAC total score, pain score and joint function score in patients with mild OA after 12 weeks, with good tolerability [592]. Health economy analysis highlight HA's value and economic benefits in KOA management. HA treatment reduces opioid and corticosteroid use as rescue therapies and delays the need for TKA [593]. At present, HA products vary in molecular weight, source, and structure, which impact efficacy. Meta‐analysis indicates that high‐molecular‐weight HA provides better pain relief than low‐molecular‐weight HA [594, 595, 596]. Moreover, avian and cross‐linked products may exhibit more inflammatory events than nonavian and noncross‐linked HAs [594], suggesting that differences in the size, source, and structure of HA can have an impact on clinical outcomes.

#### 5.1.3 Platelet‐Rich Plasma

PRP is an autologous biological agent enriched in platelets obtained via blood centrifugation, containing bioactive molecules that promote tissue repair and regeneration [597, 598]. In sports medicine, PRP injections are typically used for patients with muscle strains, tendinitis or early OA to relieve pain and accelerate recovery [599]. Due to the lack of high‐quality evidence on symptom relief and joint structure improvement, most clinical guidelines do not recommend the use of PRP for OA treatment. Despite limited high‐quality evidence for symptom relief and structural improvement its use in KOA has increased annually [600, 601].

A 1‐year randomized clinical trial compared the short‐term and long‐term efficacy of HA, PRP, plasma rich in growth factors (PRGF), and O_3_ IAIs, and found that O_3_ had the best effect in improving pain, stiffness and function of KOA at the 2‐month follow‐up. HA, PRP, and PRGF outperformed O_3_ at 6 months, and PRP/PRGF were superior to HA/O_3_ at 12 months [602], supporting O_3_ for short‐term use and PRP/PRGF for long‐term management. Bansal et al. optimized the therapeutic dose and concentration of PRP through a new manual preparation method and studied its association with physiological efficacy. Adding a 1‐micron filtration step could increase the platelet recovery rate to 90%, significantly improve the WOMAC score, International Knee Documentation Committee (IKDC) score and the distance of painless walking for 6 min in patients with KOA, and the therapeutic effect could last for more than 1 year. Furthermore, in the PRP formula, an absolute count of 10 billion platelets is crucial for providing a long‐lasting joint protection effect of up to 1 year in moderate KOA [603]. Compared with corticosteroids, the intra‐articular PRP injection was more effective in managing the symptoms of KOA during the 12‐month follow‐up, including alleviating pain, reducing joint stiffness and improving exercise capacity. In addition, three PRP injections, each 1 week apart, seem to be more effective than a single injection [604]. Multiple meta‐analyses show that PRP treatment is more effective than HA for patients with KOA, with leukocyte‐poor PRP superior to leukocyte‐rich PRP [605, 606, 607, 608, 609]. Platelet concentration can also affect the safety and clinical efficacy of PRP injection therapy, and higher platelet concentrations correlate with greater clinical improvement at 12 months [610].

PRP can promote the chondrogenic differentiation of stem cells and facilitate cartilage repair. Zhang et al. incorporated extracellular vesicles derived from PRP (PRP‐EXO) into thermosensitive hydrogels (Gels) to form an (Exo‐Gel) system, which enable 28‐day sustained release of PRP‐EXO, promoting the proliferation, migration and chondrocyte differentiation of mBM‐MSCs and inhibiting chondrocyte degeneration [423]. Exo‐Gel increased the retention rate of PRP‐Exo in joints and inhibited the apoptosis and hypertrophy of chondrocytes, improving subtalar OA [423]. PRP‐Exo encapsulated in thermosensitive gel has a good therapeutic effect on OA, which also provides a new cell‐free therapy method for the treatment of OA [423]. 3D gelatin microspheres encapsulating PRP provided the possibility of adhesion surfaces and 3D cell‐cell contact and promoted chondrogenic differentiation of MSCs [611]. Coculture of mBM‐MSCs with KGN‐PRP gel could promote chondrogenic differentiation and cartilage repair [612], suggesting that there is a “synergistic effect” between PRP and other molecules such as KGN or TGF‐β1 in promoting cartilage regeneration. Another study showed that PRP promoted differentiation of human ADSCs and cartilage regeneration via targeting insulin‐like growth factor 1 receptor and mammalian target of rapamycin (mTOR) signaling [613, 614].

However, clinical evidence remains controversial. Although PRP was also proven to improve the WOMAC and VAS scores of hip OA patients at 6‐month and 12‐month follow‐ups, PRP plus HA treatment showed no advantage over HA alone [615]. A randomized clinical trial conducted in Australia found that IAI of PRP did not significantly improve the symptoms or joint structure of patients with symptomatic mild to moderate KOA at 12 months compared with saline placebo [616]. Another randomized controlled trial conducted in the Netherlands also found no difference in AOA symptom/function improvement compared with placebo [617, 618]. The latest consensus from the European Society for Sports Trauma, Knee Surgery and Arthroscopy, and the International Society for Cartilage Regeneration and Joint Preservation recommends PRP injection for patients ≤ 80 years with KOA (K–L 0–III) after failed conservative/injectable therapy. However, PRP injection is not recommended for K–L Grade IV OA patients or as the first choice of treatment [619]. Cost‐effectiveness analysis confirm intra‐articular PRP injection is a cost‐effective treatment option for patients with mild and moderate KOA compared with growth factor‐enriched plasma (PRGF), HA, or O_3_ [620].

There is an increasing amount of evidence suggesting that PRP can regulate cartilage breakdown, inflammation and bone remodeling, and act on multiple aspects of the pathophysiology of KOA, and most controlled clinical trials and meta‐analyses support the use of PRP to treat KOA [621, 622, 623]. However, the use of PRP in clinical practice remains controversial [600]. There are still significant differences in the use of preparations, treatment regimens, extended follow‐up periods, patient selection, and the evaluation of clinically relevant outcomes.

#### 5.1.4 Bone Marrow Aspirate Concentrate

BMAC is an autologous cell therapy that utilizes the regenerative capabilities of MSCs, cytokines and growth factors to improve cartilage lesions such as cartilage degeneration, defects and cartilage damage in OA [624]. BMAC is prepared by aspirating bone marrow from the ilium, distal femur, pubis, or proximal humerus and centrifuging to concentrate cells [625]. While MSCs constitute a small proportion of BMAC, they are critical for paracrine signaling and recruiting endogenous MSCs [626]; their differentiation potential, immunosuppression, and self‐renewal capacity alleviate pain and improve joint function [627]. MSCs can enhance the potential of damaged cartilage healing through paracrine cell signaling [628]. In addition, allogeneic MSCs may restore the meniscus volume of some patients and promote meniscus regeneration [629]. The growth factors in BMAC include platelet‐derived growth factor, TGF‐β, bone morphogenetic proteins (BMP‐2 and BMP‐7), and IL‐1 receptor antagonists (IL‐1Ra), with IL‐1Ra concentrations significantly higher than in PRP [630].

Centeno et al. studied the impact of cell count in BMAC on therapeutic outcomes and found that both high‐ and low‐dose BMAC injections led to significant improvements in pain and functional results, with higher cell concentrations yielding better pain relief [631], indicating that cell dose may be an important factor influencing the clinical outcome of autologous BMAC treatment for KOA. A randomized controlled trial of patients with KL Grade II–IV KOA showed that BMAC injection reduced the VAS score, with superior 12‐month clinical improvement of patients compared with PRP/HA [632]. In short‐term and medium‐term follow‐ups, BMAC injection reduced pain in patients with hip OA, and improved joint function and overall QoL of patients [633]. Other studies have also shown encouraging results in improving pain and function in patients with OA through BMAC injection [634, 635, 636].

In contrast to these findings, a prospective, randomized, double‐blind clinical trial involving 93 patients found that autologous BMAC injection did not lead to significant changes in the National Knee Documentation Committee (IKDC) score or imaging results 1 year after meniscectomy [637]. Another study involving 25 patients found no difference between BMAC and saline [638]. Systematic reviews have shown that BMAC injection is effective in alleviating pain in patients with KOA during short‐term to medium‐term follow‐ups [639]. However, the clinical superiority of BMAC compared with other biological therapies has not been confirmed [640, 641]. The differences in BMAC preparation methods, injection regimens (single dose versus multiple administrations, intra‐articular versus subchondral), the complexity of subchondral drug administration and patient selection criteria, as well as the issues of economic feasibility and cost effectiveness, all limit its wide application [642]. Large, cautious randomized controlled trials are needed to further clarify the true therapeutic benefits of BMAC for OA.

#### 5.1.5 Ozone

O_3_ injection alleviates OA symptoms by delivering O_3_ to the joint cavity or surrounding tissues, leveraging its anti‐inflammatory and analgesic properties. A double‐blind randomized controlled trial found intra‐articular O_3_ and HA have comparable efficacy in alleviating pain in KOA [643]. This result was also confirmed in the meta‐analysis [644]. O_3_ combined with HA injection is superior to HA alone in the treatment of KOA [645]. A multicenter randomized controlled trial showed that oxygen‐O_3_ therapy was more effective than steroid injections in improving the function and relieving pain in patients with KOA [646]. O_3_ also outperformed low‐intensity laser therapy in reducing joint pain, improving the WOMAC score, and increasing joint ROM [647]. Compared with 0 γ and 10 γ, 30 γ O_3_ injection was more effective in reducing proinflammatory cytokines in synovial fluid and improving the overall antioxidant status [648], while 20 and 40 µg/mL were equally effective in reducing pain and improving functional activity in patients with KOA [649]. These seemingly contradictory results indicate that the importance of O_3_ injection dosage in treating KOA still requires further verification. Recent studies have confirmed that O_3_ injections have a rapid effect. O_3_ injections exhibited rapid short‐term efficacy (superior at 2 months) but loses effectiveness by 6 months [602]; combining it with exercise therapy alleviated pain and improves function/activities of daily living in mild‐to‐moderate KOA over 3–6 months [650]. O_3_ injection provides effective short‐term OA symptom relief, but limited evidence supports long‐term efficacy, necessitating additional studies to confirm its safety and durability.

### 5.2 Minimally Invasive Interventional Treatment for OA

Minimally invasive interventional therapy, characterized by minimal trauma, safe operation, short procedural time, significant efficacy, and rapid postoperative recovery, serves as a critical alternative for OA patients who fail traditional conservative treatment but are ineligible for or unwilling to undergo invasive surgery.

#### 5.2.1 Radiofrequency Ablation

Radiofrequency ablation (RFA) is an image‐guided interventional technique that generates thermal energy via radiofrequency current at the tip of a catheter/needle to precisely ablate abnormal lesions or hyperplastic tissues, thereby restoring tissue/organ function, improving patient QoL, and inhibiting disease progression [651]. It is increasingly being used for chronic OA‐related pain, demonstrating significant efficacy in pain relief and functional recovery [652, 653, 654]. A 12‐month follow‐up data study showed ultrasound‐guided RFA of hip nerve branches significantly reduced pain scores in hip OA patients, and more than 75% of the patients reported more than 50% pain relief during the follow‐up period, 85% of the patients reduced their use of painkillers, and no side effects were reported [655], proving that RFA of the hip nerve branches has good effectiveness and safety in improving chronic hip pain. Similar therapeutic effects have been achieved in OA of the first carpometacarpal joint [656]. In patients with KOA, RFA of knee nerves not only relieved pain and improved joint function, but also preserved static balance control and quadriceps strength, with positive improvements in proprioception [657]. Multiple meta‐analyses of randomized controlled trials have demonstrated that ultrasound‐guided RFA effectively alleviates KOA pain and promotes functional recovery [658, 659, 660]. The improvement of knee joint function in elderly patients has also achieved high patient satisfaction, providing a feasible, safe, and effective minimally invasive option for moderate to severe KOA in the elderly [661].

Compared with other therapies such as NSAIDs and corticosteroid injections, RFA has reported fewer adverse reactions in clinical practice [662, 663]. Compared with IACS and HA injections, RFA‐treated patients showed superior improvements in Oxford Knee Score and WOMAC scores [664]. At 3‐ and 6‐month follow‐ups, RFA achieved better WOMAC score improvements than intra‐articular MSC injection in KOA [665]. However, no consistent efficacy difference exists between RFA and intra‐articular PRP therapy for alleviating the pain of patients with KOA [666, 667].

Advanced RFA modalities further expand its application, and pulsed and cooled radiofrequency have also been more widely used in interventional treatments for patients with chronic pain [668]. Cooled RFA (CRFA) significantly reduced pain in KOA, improved patient function, and reduced analgesic needs, with effects lasting up to 2 years [669, 670]. A study on Japanese patients with KOA found that CRFA could improve the VAS scores at rest and during walking, as well as the average walking speed, and walking stability [671]. Compared with single HA injection, CRFA treatment outperforms in pain relief and overall function [672]. Pulsed RFA not only significantly alleviated pain but also enhanced their physical fitness [673, 674], with therapeutic effects lasting 12 weeks for refractory knee pain [675]. Wu et al. found that patients with KOA responded better to CRFA than traditional/pulsed modes. Bipolar RFA is more effective than monopolar RFA in pain and function improvement [676]. Comparative studies of RFA subtypes showed that radiofrequency thermocoagulation had superior long‐term efficacy to intra‐articular pulsed radiofrequency for KOA [677], and continuous neuroablative radiofrequency achieves more significant pain relief and greater analgesic reduction than pulsed RFA [678]. Multiple randomized controlled trials have shown that RFA is a safe and effective minimally invasive therapy for OA‐related pain, with superior efficacy to conventional injectable therapies in specific scenarios; however, consistent evidence comparing it with PRP is lacking, and further large‐scale trials are required.

#### 5.2.2 Arthroscopy

Arthroscopy is a minimally invasive procedure integrating diagnosis and treatment, involving debris removal from the joint cavity and meniscal repair. It is suitable for moderate OA patients who fail conservative treatment or have limited joint mobility. Among patients aged more than 40 years, hip arthroscopy combined with postoperative physical therapy achieved better treatment outcomes compared with physical therapy alone [679]. Hip arthroscopy significantly improved the PRO score of patients with Tönnis Grade Ⅰ OA [680]. Medium to long‐term follow‐ups confirmed PRO improvements in patients undergoing initial hip arthroscopy [681, 682]. In young patients, hip arthroscopy had a preventive effect on the development and progression of OA [683], with a 12‐year follow‐up study showing 25% of patients had improved Tönnis classification and a 42% reduced relative risk of OA progression [684]. For patients with mild to moderate OA, hip arthroscopic surgery can provide significant long‐term functional benefits and reduce the incidence of total hip arthroplasty (THA) [685]. However, the systematic review found contradictory evidence regarding the postoperative outcomes of patients with OA after hip arthroscopy. The proportion of patients who underwent hip arthroscopy followed by total hip replacement varied from 0 to 70% across different studies [686]. For patients with severe OA, arthroscopy may only provide temporary relief without addressing severe cartilage wear. The severity of OA before the surgery is a key predictor of THA conversion.

Arthroscopy is currently less commonly used in the treatment of KOA due to high‐quality evidence questioning its efficacy for alleviating the symptoms [687]. A secondary analysis of a randomized clinical trial found that knee arthroscopy had no impact on TKA incidence in patients [688]. Gu et al. reported a time‐dependent relationship between knee arthroscopy timing and TKA complications, found shorter interval increased revision surgery and periprosthetic joint infection (PJI) risks, To minimize PJI risks of and revision surgery, an interval of at least 36 weeks should be maintained [689].

The microfragmented adipose tissue (MFAT) rich in MSCs and growth factors has become a promising biomaterial for cartilage repair and inflammation inhibition. The combined treatment of arthroscopy and autologous MFAT injection significantly improved the IKDC score and KOOS score of patients with KOA with no adverse reactions [690]. A 2 years follow‐up confirmed sustained clinical and functional improvements, unaffected by age, BMI, or OA pathological stage [691]. Meta‐analysis showed that MFAT and knee arthroscopy combined have significant efficacy in pain relief and joint function improvement, particularly in patients with early lesions or local cartilage damage [692]. Arthroscopic surgery is effective in relieving pain in the thumb caused by OA of the metacarpophalangeal joint, improving hand dexterity and enhancing daily activities [693].

Arthroscopy is effective for mild‐to‐moderate hip OA and thumb OA, but its value in KOA is limited; combined with MFAT, it shows promise for KOA with local cartilage damage, while severe OA patients may only experience temporary relief.

#### 5.2.3 Articular Irrigation

Ultrasound‐guided articular irrigation involves puncture‐mediated injection of normal saline or other fluids into the joint cavity to flush and remove inflammatory substances, necrotic tissue, or effusions. It is indicated for OA, RA, and posttraumatic joint effusion [694]. Compared with drugs, physical therapy or injection treatment, arthritic irrigation more effectively removes inflammatory mediators, alleviates cartilage and synovial inflammation, and improves functional mobility [695]. A retrospective case study found that articular irrigation significantly relieves KOA‐related pain for at least 6 months, however, for patients with long‐lasting pain, severe bone marrow lesions, and severe cartilage loss, the efficacy of joint lavage is limited [696]. Analysis of synovial fluid before and after irrigation revealed that the pain relief and functional improvement might be related to the reduction of inflammatory markers (IL‐1β, IL‐6, TNF‐α), cartilage‐degrading enzyme MMP‐3, and oxidative stress, with moderate correlations between IL‐6/TNF‐α reduction and VAS/WOMAC score improvements [697]. Articular irrigation enhances the efficacy of subsequent injectable therapies. For instance, O_3_ perfusion combined with irrigation effectively alleviated clinical symptoms, increased the levels of bone metabolism indicators osteocalcin and osteoprotegerin, reduced the levels of RANKL and inflammatory levels, and contributed to the recovery of knee joint function [698].

Articular irrigation alleviates OA inflammation and pain by removing pathogenic substances, with enhanced efficacy when combined with other therapies; however, it is less effective for patients with severe joint damage.

#### 5.2.4 Genicular Artery Embolization

Genicular artery embolization (GAE) is an image‐guided minimally invasive technique that blocks abnormal neovascularization in the affected knee joint, reducing inflammatory factor release and pain signal transmission to alleviate symptoms. Initially used for tumor hemorrhage, it has gained attention in OA treatment due to its precision and minimal invasiveness [699, 700]. Clinical evidence showed sustained efficacy in young patients and those with early mild‐to‐moderate KOA, with significant improvements in KOOS sports, QoL, and global change scores [701, 702]. Patients with severe KOA can also benefit from reduced pain symptoms and functional improvement, and good tolerance [703, 704]. Cost‐effectiveness analysis based on data from randomized clinical trials identified GAE as the most cost‐effective option compared with RFA and corticosteroid treatment [705].

Although GAE has demonstrated effectiveness and safety in some clinical trials, the benefits it offers over placebo remain uncertain [706]. Significant heterogeneity exists across clinical trials in research design, procedural techniques, embolization materials, follow‐up time points, and MRI parameter reporting. Its application in OA is limited by research heterogeneity, inconsistent naming of embolization areas, and short follow‐up durations [706].

GAE shows promising efficacy and cost effectiveness for KOA, particularly in young and mild‐to‐moderate patients; however, uncertainties regarding placebo‐controlled benefits and research heterogeneity hinder its widespread adoption. Future standardized protocols are needed to facilitate meta‐analyses and robust evidence‐based evaluation of GAE's efficacy and safety.

Minimally invasive interventional therapies provide valuable options for OA patients failing conservative treatment, with modality‐specific advantages. RFA excels in pain management, arthroscopy is effective for hip/thumb OA and KOA with local damage, articular irrigation enhances subsequent therapy efficacy, and GAE offers cost‐effective KOA treatment. However, inconsistent evidence, limited data for severe OA, and research heterogeneity highlight the need for standardized protocols and large‐scale trials to optimize clinical application.

### 5.3 Surgical Treatment

Surgical treatment constitutes a critical component of the staged management of OA, primarily indicated for patients with refractory symptoms or advanced joint damage who fail conservative and minimally invasive therapies. The core goals of OA surgery are to correct mechanical abnormalities, relieve pain, restore joint function, and delay or avoid joint replacement.

#### 5.3.1 Osteotomy

Osteotomy is a joint‐preserving surgical procedure for single‐compartment OA, aiming to correct lower limb alignment and redistribute joint loads from the damaged compartment to the relatively intact one. Its most prominent advantage is preserving the native joint anatomy, maintaining proprioception, enabling rapid functional recovery, and significantly delaying OA progression, making it particularly suitable for younger patients with good residual cartilage function.

##### 5.3.1.1 High Tibial Osteotomy

HTO is a limb alignment correction surgery designed to redistribute knee joint loading, preserve joint structure, and improve clinical outcomes. It is primarily indicated for KOA with varus deformity, commonly in medial compartment OA [707]. Clinical studies have found that HTO reduced the load on the medial knee joint and alleviated knee joint inflammation [708], reduced synovial fluid levels of proinflammatory cytokines (IL‐6, IL‐8) and cartilage‐degrading enzymes (MMP‐2, MMP‐3, MMP‐13), and delayed cartilage damage [709]. Mechanistically, HTO corrects lower limb alignment, mitigates synovial inflammation, and promotes macrophage polarization from proinflammatory M1 to anti‐inflammatory M2 phenotypes, improving the intra‐articular microenvironment in KOA [710].

However, the long‐term impact of HTO on the progression of patellofemoral joint OA remains unclear. A retrospective study followed 95 patients who underwent open wedge HTO (OWHTO). During a minimum 7‐year follow‐up, 50.5% of the patients showed progression of patellofemoral OA on imaging, though no significant differences in clinical outcomes were observed between progression and nonprogression groups [711]. The influence of medial OWHTO on the progression of OA is relatively mild, and current evidence is insufficient to draw definitive conclusions regarding clinical implications [712].

To address the uncertain long‐term efficacy of standalone HTO, combination therapies have been explored. Implantation of human umbilical cord blood‐derived MSCs (hUCB‐MSCs) with HTO for medial compartment OA and varus deformity significantly enhanced cartilage regeneration and overall clinical outcomes [713]. The combination of HTO with autologous adipose‐derived MSCs (AD‐MSCs) also alleviated the severity of KOA in patients by delaying the aging of chondrocytes and providing antioxidant effects [713]. HTO is an effective joint‐preserving surgery for young patients with medial compartment KOA and varus deformity, though its long‐term impact on patellofemoral OA is unclear; combination with MSCs may further enhance cartilage regeneration and therapeutic efficacy.

##### 5.3.1.2 Proximal fibular osteotomy

Proximal fibular osteotomy (PFO) is a relatively novel surgical method for treating medial compartment KOA. Similar to HTO, it corrects lower limb alignment to redistribute loads between medial and lateral compartments, reducing medial cartilage wear and pain. The difference is that PFO is based on the “nonuniform settlement” theory. It involves removing a small segment of the proximal fibula, specifically below the fibular head, cutting the fibula and weakening its support on the lateral side of the tibial plateau. This ultimately reduces the lateral gap of the knee joint and compensates for the knee valgus deformity caused by weight‐bearing [714]. Finite element analysis demonstrated that PFO induced significant changes in knee joint peak stress and distribution, shifting stress from the medial to the lateral compartment and repositioning the pressure center to the joint midline [715, 716, 717].

A prospective cohort study demonstrated that within 1 year after undergoing PFO surgery for KOA, the VAS score, American Knee Society Score, medial–lateral knee joint space ratio (ML ratio), and K–L grade were all improved, significantly alleviating the patient's pain and improving joint function [718, 719]. Multiple retrospective studies have confirmed that lateral patellar joint decompression via PFO alleviated pain and improved the function of the knee joint [720]. Most patients achieved a high level of satisfaction during the long‐term follow‐up after the surgery [721]. Meta‐analysis further validated PFO's promising role in medial compartment KOA management [722], especially for medial KOA patients with an upward curvature of the fibula [723, 724].

Despite these positive findings, the evidence base for PFO remains low‐quality, with limited imaging studies and a lack of diverse patient populations. Higher‐quality clinical evidence is needed to confirm its efficacy and generalizability.

##### 5.3.1.3 Distal Femoral Osteotomy

Distal femoral osteotomy (DFO) is a surgical procedure for correcting knee joint deformities or OA by adjusting the distal femoral bone morphology to restore lower limb alignment, relieve pain, and delay joint replacement. At present, there are relatively few relevant literatures on DFO. Limited evidence suggests that DFO is effective in treating patients with lateral compartment OA and varus knee, and the incidence of complications is relatively low [725]. About 1 year after DFO surgery, patients with OA can resume low‐intensity exercise [726]. DFO shows promise for lateral compartment KOA with valgus deformity, though limited clinical data highlight the need for additional studies to confirm its long‐term efficacy.

#### 5.3.2 Joint Replacement Surgery

Joint replacement surgery is the ultimate step in OA staged treatment, indicated for patients with severe joint damage, refractory symptoms, and impaired daily living activities despite conservative and conventional surgical interventions. Technological advancements (e.g., navigation, robot‐assisted surgery) have improved implant positioning and alignment accuracy, contributing to excellent long‐term outcomes. Approximately 90% of patients with hip arthritis have their artificial joints functioning normally 15 years after the surgery, while 80% of patients with KOA can use an artificial total knee joint prosthesis for more than 25 years [727].

##### 5.3.2.1 Total Hip Arthroplasty

THA is the most effective treatment for end‐stage hip OA, significantly improving the QoL of patients, alleviating pain, and restoring the reduced range of joint movement [728]. A multicenter, randomized, controlled trial compared the effects of THA and resistance training in patients aged more than 50 years with severe hip OA and surgical indications and found that THA significantly reduced hip pain and improved function at 6 months compared with resistance training [71]. One year after THA, the physical function and lower limb muscle strength of the patients have significantly improved, with 83.7% reporting satisfaction [729, 730]. The pelvic inclination angle (the angle formed by the vertical line from the midpoint of the sacral plate to the line connecting the centers of the two acetabula) might be an important factor affecting postoperative satisfaction [731]. A randomized controlled trial compared the effects of the short‐axis, proximal porous‐coated Tri‐Lock bone‐preserving stem and the conventional full‐coated Corail prosthesis on the bone volume of the proximal femur after THA. It was found that the Tri‐Lock bone‐preserving stem and Corail prosthesis yield similar changes in bone metabolism markers, PRO measures, and adverse event rates, suggesting bone‐preserving prostheses do not necessarily outperform traditional designs [732]. Early mobilization after THA is crucial for a rapid recovery, but it is often limited by pain. preoperative and postoperative administration of an oral enzyme combination (bromelain, trypsin, rutin) reduces CRP levels and pain, facilitating post‐THA recovery [733]. During the early rehabilitation stage of THA, both the conventional physical therapy (CPT) program and task‐oriented exercise (TOE) reduced pain and improved the function of the operated leg as well as its static and dynamic balance. Compared with CPT, TOE showed a better improvement effect on hip joint function [734]. Six weeks of preoperative teriparatide administration promoted bone formation after THA [735].

##### 5.3.2.2 Total Knee Arthroplasty

TKA involves resecting damaged bone tissue and implanting artificial prostheses to replace severely compromised knee joint components, restoring function and relieving pain. It is primarily indicated for advanced OA, RA, and traumatic arthritis, with proven clinical efficacy [736, 737]. More research focuses on optimizing TKA outcomes. A 48‐month follow‐up study revealed that the portable handheld accelerometer‐based navigation in TKA had comparable alignment accuracy and functional outcomes to the large console, image‐free computer‐assisted navigation, and could shorten the operation time [738]. Compared with the traditional instrument‐based TKA based on fixtures, the image‐free handheld robot‐assisted TKA achieved better alignment of the limbs [739]. personalized TKA based on preoperative imaging reduced postoperative patellar inclination and improved clinical outcomes regardless of preoperative phenotype [740].

Prosthesis design advancements also enhance outcomes. Compared with the standard symmetrical cruciate retaining (CR) bearing design surgery, the surgery based on the medial congruent anatomic bearing design more closely mimic natural knee kinematics and improve movement stability [741]. AI, such as ChatGPT, is increasingly integrated into TKA to enhance patient satisfaction, highlighting intelligence and personalization as future development directions [742]. It is worth noting that more than one fifth of the patients did not experience an improvement in their physical functions after undergoing TKA, which may be related to the higher preoperative BMI and poorer physical function [743]. Allergic reactions to implant materials may affect the therapeutic effect, and patients with increased inflammatory responses have worse functional outcomes [744].

Osteotomy is suitable for younger patients with single‐compartment OA, while joint replacement is the ultimate option for end‐stage disease. Technological innovations such as robot‐assisted surgery, personalized prostheses, and combination therapies improve outcomes, though low‐quality evidence for PFO and preoperative patient factors remain challenges to address in future research.

## 6 Conclusion and and Future Perspectives

### 6.1 Current Challenges and Limitations in OA Management

As a heterogeneous and progressive musculoskeletal disorder, OA imposes a growing global health burden, driven by aging populations, rising obesity rates, and increased prevalence of joint trauma. Over the past few decades, considerable progress has been made in the clinical management of OA, yet reliable tools for early and preclinical diagnosis remain scarce, and most approved interventions remain palliative and symptom‐oriented. Current treatments can effectively relieve pain and reduce inflammation but fail to reverse established cartilage degeneration or halt disease progression, and the therapeutic response varies dramatically among individuals. Meanwhile, most of the existing biomarkers lack sufficient sensitivity and specificity for early OA screening, and few biomarkers have been fully validated in large‐scale longitudinal cohorts and standardized clinical protocols. The pathological mechanisms underlying OA heterogeneity, including subtype‐specific features, site‐specific differences, remain incompletely understood. In addition, most emerging therapies such as stem cell preparations, extracellular vesicles, and nanomaterials are still at the preclinical or early clinical stage, lacking high‐level evidence from Phase III randomized controlled trials to support clinical application. These unmet clinical challenges severely restrict efforts to prevent or delay OA progression, highlighting the urgent demand for improved early diagnostic technologies and novel disease‐modifying OA therapeutic drugs.

### 6.2 The Paradigm Shift Toward Precision Medicine

A core direction of future OA research lies in establishing biomarker‐driven patient stratification, representing a pivotal step toward advancing precision and preventive OA care. Ideal biomarkers should be noninvasive, economical, and widely applicable, enabling early detection before obvious structural damage, accurate risk stratification and prognosis prediction, and objective monitoring of therapeutic efficacy. Multiomics strategies encompassing genomics, transcriptomics, proteomics, and metabolomics support systematic dissection of OA pathogenesis at the molecular level and accelerate the discovery of sensitive and specific biomarkers for early diagnosis and prognosis. Reliable imaging indicators and molecular biomarkers will assist clinicians in stratifying high‐risk patients for early intervention, avoid overtreatment in patients with stable mild OA, and realize refined patient stratification. While this biomarker‐guided precision care paradigm is still in its early stages, it holds significant promise for developing more effective and targeted interventions that address OA's heterogeneity, ultimately transforming OA management from reactive symptom control to proactive, personalized disease prevention.

### 6.3 Technological Innovations and Emerging Therapeutic Frontiers

The landscape of OA research is rapidly evolving driven by cutting‐edge technological innovations. Advanced imaging modalities including MRI, ultrasound elastography, and optical coherence tomography enable early detection of subtle structural damage prior to overt clinical symptoms. Complemented by AI and machine learning algorithms, these tools greatly enhance diagnostic precision, risk stratification, progression monitoring, and treatment response evaluation, laying a solid foundation for precision OA management. Meanwhile, the therapeutic paradigm of OA is shifting from symptomatic relief toward structure‐modifying and regenerative solutions. Emerging interventions including MSC therapy, tissue engineering, and gene therapy demonstrate encouraging potential to repair damaged cartilage, suppress pathological inflammation, and restore joint function. Moreover, innovative intraarticular delivery systems including NPs and hydrogel based sustained release platforms improve local bioavailability in the joint microenvironment while reducing systemic side effects. Together, these innovations are moving OA treatment beyond passive symptom control toward active, mechanism‐driven disease modification.

### 6.4 Summary and Outlook

In summary, OA research is entering a transformative era empowered by technological innovation and deepening mechanistic insights, steadily advancing toward a more precise and individualized care paradigm. To address the aforementioned research limitations, including insufficiently validated biomarkers, unclear heterogeneity mechanisms, and inadequate clinical translation of emerging therapies, future research should prioritize elucidating the complex molecular mechanisms of OA, identifying novel therapeutic targets, and promoting the clinical translation of robust biomarkers. Interdisciplinary collaboration and effective translation of technological innovations into clinical practice will further drive this progress. Collectively, these advances will enable early diagnosis, personalized intervention, and effective prevention of OA, thereby revolutionizing its clinical management, substantially reducing the global health burden, and improving long‐term outcomes for patients worldwide.

## Author Contributions

All authors have made substantial contributions to the conception and design of the review, acquisition of data, analysis and interpretation of data, drafting the article, or revising it critically. Qin Ru contributed to the collection, collation, and analysis of literature related to OA diagnostic biomarkers and treatment, participated in drafting the corresponding sections of the manuscript, and revised the manuscript critically. Dongliang Yuan participated in the systematic collation of OA epidemiology and assisted in revising the related sections of the manuscript. Shide Jiang contributed to the collection and analysis of literature on OA comorbidities and their impacts. Xiaoxuan Zhao and Xu Liu assisted in the collation of imaging diagnostic markers and fluid‐based biomarkers related data. Yusheng Li, Shuguang Liu, and Yuxiang Wu conceptualized and designed the overall framework of the review, guided the literature collection and data analysis, and supervised the entire research and manuscript revision process. All authors have read and approved the final manuscript.

## Funding

This work was supported by the National Key R&D Programof China (No. 2023YFC3603400), Natural Science Foundation of China (No. 82372500, 82072494, 82072506, 92268115), Science and Technology Innovation Program of Hunan Province (No. 2021RC3025), National Clinical Research Centerfor Geriatric Disorders (Xiangya Hospital, No. 2021KF02), National High Level Hospital Clinical Research Funding (2022‐NHLHCRF‐YGJE‐05), and Basic Research Project of Jianghan University (No. 2025JCYJ07).

## Ethics Statement

The authors have nothing to report.

## Conflicts of Interest

The authors declare no conflicts of interest.

## Data Availability

The authors have nothing to report.
